# Associations between type 1 diabetes and pulmonary tuberculosis: a bidirectional mendelian randomization study

**DOI:** 10.1186/s13098-024-01296-x

**Published:** 2024-03-05

**Authors:** Yijia Jiang, Wenhua Zhang, Maoying Wei, Dan Yin, Yiting Tang, Weiyu Jia, Churan Wang, Jingyi Guo, Aijing Li, Yanbing Gong

**Affiliations:** Dongzhimen Hospital, Beijing University of Chinese Medicine, 100700 Beijing, China

**Keywords:** Type 1 diabetes, Pulmonary tuberculosis, Causal effects, Mendelian randomization

## Abstract

**Background:**

Type 1 diabetes mellitus (T1DM) has been associated with higher pulmonary tuberculosis (PTB) risk in observational studies. However, the causal relationship between them remains unclear. This study aimed to assess the causal effect between T1DM and PTB using bidirectional Mendelian randomization (MR) analysis.

**Methods:**

Single nucleotide polymorphisms (SNPs) of T1DM and PTB were extracted from the public genetic variation summary database. In addition, GWAS data were collected to explore the causal relationship between PTB and relevant clinical traits of T1DM, including glycemic traits, lipids, and obesity. The inverse variance weighting method (IVW), weighted median method, and MR‒Egger regression were used to evaluate the causal relationship. To ensure the stability of the results, sensitivity analyses assess the robustness of the results by estimating heterogeneity and pleiotropy.

**Results:**

IVW showed that T1DM increased the risk of PTB (OR = 1.07, 95% CI: 1.03–1.12, *P* < 0.001), which was similar to the results of MR‒Egger and weighted median analyses. Moreover, we found that high-density lipoprotein cholesterol (HDL-C; OR = 1.28, 95% CI: 1.03–1.59, *P* = 0.026) was associated with PTB. There was no evidence of an effect of glycemic traits, remaining lipid markers, or obesity on the risk of PTB. In the reverse MR analysis, no causal relationships were detected for PTB on T1DM and its relevant clinical traits.

**Conclusion:**

This study supported that T1DM and HDL-C were risk factors for PTB. This implies the effective role of treating T1DM and managing HDL-C in reducing the risk of PTB, which provides an essential basis for the prevention and comanagement of concurrent T1DM and PTB in clinical practice.

**Supplementary Information:**

The online version contains supplementary material available at 10.1186/s13098-024-01296-x.

## Introduction

Type 1 diabetes mellitus (T1DM) is a chronic autoimmune disease characterized by absolute insulin deficiency and hyperglycemia [1]. It usually occurs in childhood and adolescence, accounting for approximately 5–10% of all diabetes cases [2, 3]. There is a growing trend in the prevalence of T1DM globally, with an increase of approximately 2–3% per year. It is predicted that 13.5–17.4 million people worldwide will have T1DM by 2040 (60–107% higher than in 2021), leading to a heavy burden on families and economies [4, 5]. Pulmonary tuberculosis (PTB) is a chronic infectious disease caused by *Mycobacterium tuberculosis*(Mtb) [6]. According to the World Health Organization (WHO), approximately 10.6 million people suffered from TB in 2021, an increase of 4.5% compared to 2020 [7, 8]. As the complication rates of diabetes and tuberculosis increase, it is necessary to further elucidate the association to better reduce the burden of disease.

Several recent studies have suggested that T1DM is a primary risk factor for PTB. For example, a hospital-based cross-sectional study reported that the prevalence of Mtb infection in children and adolescents with T1DM was 29.8% (95% CI 24.2–35.4) [9]. Another case‒control study demonstrated that patients with diabetes had 2.66 times the risk of PTB compared to the general population [10]. A systematic evaluation of 13 observational studies identified a statistically significant association between diabetes and latent tuberculosis infection [11]. However, the results of observational studies may be affected by reverse causality and confounding factors, as well as the fact that most of the studies did not specify diabetes phenotypes. Given the differences between T1DM and T2DM in terms of etiology, pathogenesis, and underlying genetic factors, direct evidence of a causal relationship between T1DM and PTB is still currently lacking.

T1DM is frequently accompanied by disorders of glucose and lipid metabolism and obesity [12, 13]. Some studies have concluded that diabetic patients are susceptible to PTB infection, which is related to disturbed glucose and lipid metabolism in diabetic patients [14]. A cohort study indicated that an estimated 7.5% of TB occurrences were attributed to poor glycemic control [15]. Besides, it has been observed that serum high-density lipoprotein cholesterol (HDL-C), low-density lipoprotein cholesterol (LDL-C), and total cholesterol (TC) concentrations were lower in patients with PTB [16]. A systematic evaluation identified a consistent log-linear relationship between BMI and tuberculosis incidence, with a decrease in TB incidence of approximately 14% per unit increase in body mass index (BMI) [17]. Nevertheless, there are few studies and mostly observational trials. Further research is necessary to support a causal relationship between glucose, lipids, obesity, and PTB.

Mendelian randomization (MR) utilizes genetic variants as instrumental variables (IVs) to elucidate the causal relationship between exposure and outcome [18]. Due to the random assignment of genetic variants during meiosis, MR is effective in reducing biases caused by confounders and reverse causality [19]. Compared with prospective experiments, MR analysis reveals causal associations in a time-saving and cost-effective manner [20].

In the present study, we performed the first bidirectional two-sample MR analysis to assess the causal effects between T1DM and PTB.

## Methods

### Study design

**Fig. 1:**
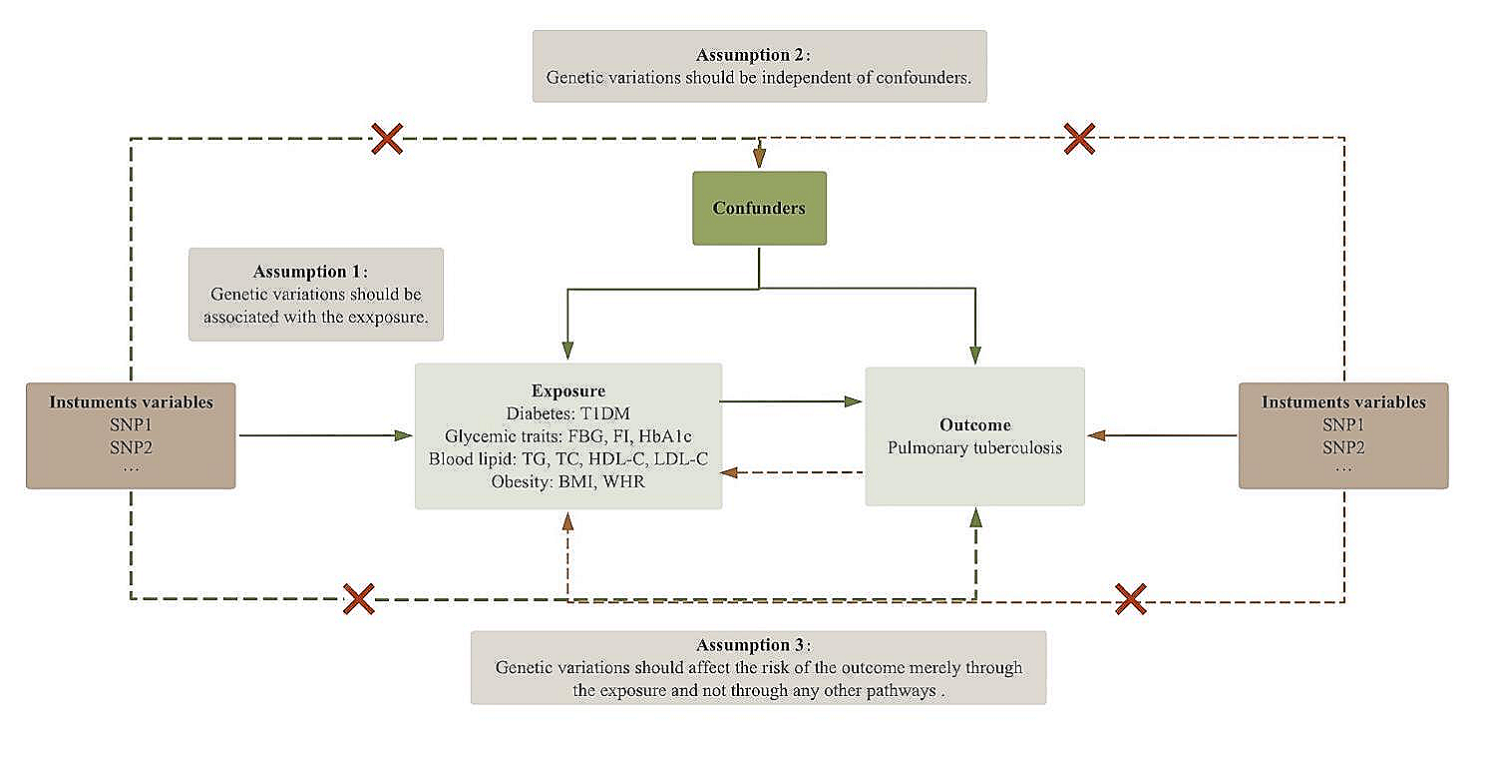
Overview of the bidirectional MR analysis. SNPs, single-nucleotide polymorphisms; T1DM, type 1 diabetes; FBG, fasting blood glucose; FI, fasting insulin; HbA1c, glycosylated hemoglobin; TG, triglycerides; TC, total cholesterol; HDL-C, high-density lipoprotein cholesterol; LDL-C, low-density lipoprotein cholesterol; BMI, body mass index; WHR, waist-to-hip ratio

A brief description of the bidirectional MR analysis is demonstrated in Fig. 1. The MR analysis was performed to explore potential causal relationships between T1DM and PTB. In addition, the relevant clinical traits of T1DM, including glycemic traits, blood lipids, and obesity, were also investigated for potential causality with PTB to explore the underlying genetic mechanisms between T1DM and PTB. There are three core assumptions of study design: first, the genetic variations used as IVs should be robustly associated with the exposure; second, the genetic variations should be independent of confounders; and third, the genetic variations should affect the risk of the outcome merely through the exposure and not through any other pathways.

### Data sources

Table 1 displays the characteristics of the results from the Genome-Wide Association Study (GWAS).

#### T1DM

The genetic instruments from the latest GWAS meta-analysis for T1DM were based on 520,580 individuals (18,942 T1DM cases and 501,638 controls) of European ancestry from the European Bioinformatics Institute (EBI) database [21].

#### Hyperglycemia

Summary statistics on glycemic traits [fasting blood glucose (FBG), glycosylated hemoglobin (HbA1c)] were extracted from the Meta-Analyses of Glucose and Insulin-related Traits Consortium (MAGIC), including 133,010 and 46,368 nondiabetic individuals of European ancestry [22, 23]. For fasting insulin (FI), extracted from the EBI database, the data consisted of 151,013 individuals of European ancestry [24].

#### Blood lipid

Data from the Global Lipids Genetic Consortium (GLGC) were used to identify genetic loci for blood lipids [HDL-C, LDL-C, TC, triglycerides (TG)], including 188,578 individuals of European ancestry and 7,898 individuals of non-European ancestry [25].

#### Obesity

Results from the Genetic Investigation of Anthropometric Traits consortium (GIANT) were used to identify genetic instruments for BMI and waist-to-hip (WHR), including 322,154 and 210,088 individuals of European ancestry [26, 27].

#### PTB

We extracted genetic variation data for respiratory tuberculosis from the FinnGen Consortium. The summary-level statistics are from a large study involving 377,277 individuals (2,432 cases and 374,845 controls); all participants were of European ancestry [28].

**Table 1:** The characteristics of the GWAS

Trait	Ancestry	Sample Size	Sex	Consortium
T1DM	European	520,580	Female and male	PMID: 34,012,112
FBG	European	133,010	Female and male	MAGIC
HbA1c	European	46,368	Female and male	MAGIC
FI	European	151,013	Female and male	PMID: 34,059,833
LDL-C	Mixed	173,082	Female and male	GLGC
HDL-C	Mixed	187,167	Female and male	GLGC
TG	Mixed	177,861	Female and male	GLGC
TC	Mixed	187,365	Female and male	GLGC
BMI	European	322,154	Female and male	GIANT
WHR	European	210,088	Female and male	GIANT
Respiratory tuberculosis	European	377,278	Female and male	FinnGen

T1DM, type 1 diabetes; FBG, fasting blood glucose; HbA1c, glycosylated hemoglobin; FI, fasting insulin; LDL-C, low-density lipoprotein cholesterol; HDL-C, high-density lipoprotein cholesterol; TG, triglycerides; TC, total cholesterol; BMI, body mass index; WHR, waist-to-hip ratio

### IV selection

Based on the three core assumptions, the IVs for MR studies were chosen: (1) single nucleotide polymorphisms (SNPs) were robustly associated with the exposure (*p* < 5e-8). Since no SNP were selected as genetic instruments for PTB that reached genome-wide significance, we adopted a less stringent threshold (*P* < 1e–5) to identify more SNPs for PTB [29]; (2) the independent SNPs were selected after excluding linkage disequilibrium (kb = 10,000, *r*^*2*^ < 0.001); (3) the F statistic was used to assess the strength of the IVs, which is calculated as ($\frac{{R}^{2}}{1-{R}^{2}}$)×($\frac{N-K-1}{K}$), where R^2^ is the degree of exposure explained by the instrument, n represents the sample size, and k is the number of SNPs. With an F statistic > 10, IVs can be identified to minimize potential weak instrumental bias [30]. (4) To avoid potential pleiotropic effects caused by confounders, we assessed the confounders influencing the incidence of PTB from previous meta-analysis studies, including smoking [31], alcohol consumption [32], anemia [33], and HIV [34]. Potentially confounding SNPs were removed via the PhenoScanner website (http://www.phenoscanner.medschl.cam.ac.uk/), as presented in Table S1.

### Statistical analysis

Several analytical methods, including inverse variance-weighted (IVW), MR‒Egger, and weighted median, were used in the MR analyses [35]. Among them, IVW was used as the primary statistical method, which provides the most accurate causal estimates assuming that all SNPs are valid instrumental variables. MR‒Egger analysis can generate estimates if significant horizontal pleiotropy is detected [36]. The weighted median method can provide accurate causal estimates when at least 50% of the weights come from valid instrumental variables [37]. The Q statistics (Cochran’s Q for IVW and Rücker’s Q for MR‒Egger) were used to assess heterogeneity in the estimates of SNPs, with p values less than 0.05 indicating the presence of heterogeneity [38]. The MR‒Egger regression intercept examination was used to estimate possible horizontal pleiotropy, and a deviation from zero indicated directional pleiotropy [36, 39]. The MR-pleiotropy residual sum and outlier (MR-PRESSO) was used to detect outlying SNPs and provide estimates after removing the outliers [40]. Statistical analysis was performed using the R packages “TwoSampleMR” and “MRPRESSO” in R software (version 4.0.2).

## Results

### Bidirectional causal effect between T1DM and PTB

**Fig. 2:**
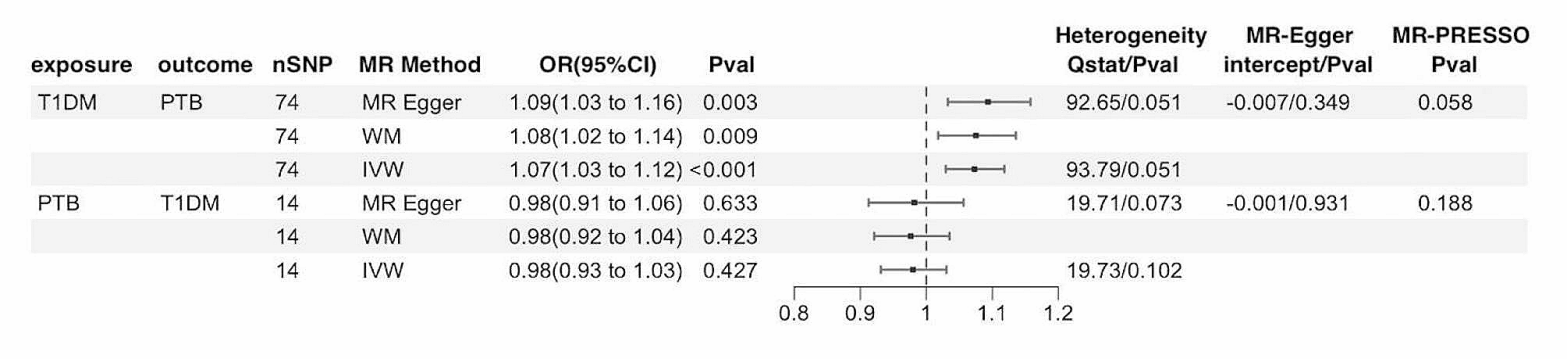
MR results for the bidirectional causal effect between T1DM and PTB. T1DM, type 1 diabetes; FBG, fasting blood glucose; FI, fasting insulin; HbA1c, glycosylated hemoglobin; SNPs, single-nucleotide polymorphisms; MR, Mendelian randomization; CI, confidence interval; OR, odds ratio; IVW, inverse variance-weighted; WM, weighted median; P value for heterogeneity based on Cochran’s Q statistic for IVW and Rücker’s Q for MR‒Egger

Figure 2 demonstrates the MR estimates between T1DM and PTB. Using 74 associated SNPs as IVs, IVW analysis showed that T1DM was correlated with the risk of PTB (OR = 1.07, 95% CI: 1.03–1.12, *P* < 0.001), with one SNP removed as an outlier (rs1794269). The results of other MR methods were consistent with IVW analysis (Fig. 2), and no heterogeneity or directional pleiotropy was detected (Table S2, Figure S1 in the Supplement). There was no evidence of reverse causation effect of PTB on T1DM (OR = 1.19, 95% CI: 0.76–1.86, *P* = 0.442). MR-PRESSO showed horizontal pleiotropy (*P* < 0.001), with four SNPs identified as outliers (rs185377955, rs2395516, rs6708458, rs72836185). When the outliers were removed, there was no significant change in the reverse causality of PTB on T1DM (OR = 0.98, 95% CI: 0.93–1.03, *P* = 0.427), which was consistent across the MR‒Egger and weighted median analyses (Fig. 2). The Cochran’s Q test and MR‒Egger regression intercept for the relationship did not reveal any evidence of directional pleiotropy or heterogeneity (Table S3, Figure S2 in the Supplement).

### Causal effect of metabolic factors and obesity on PTB

**Fig. 3:**
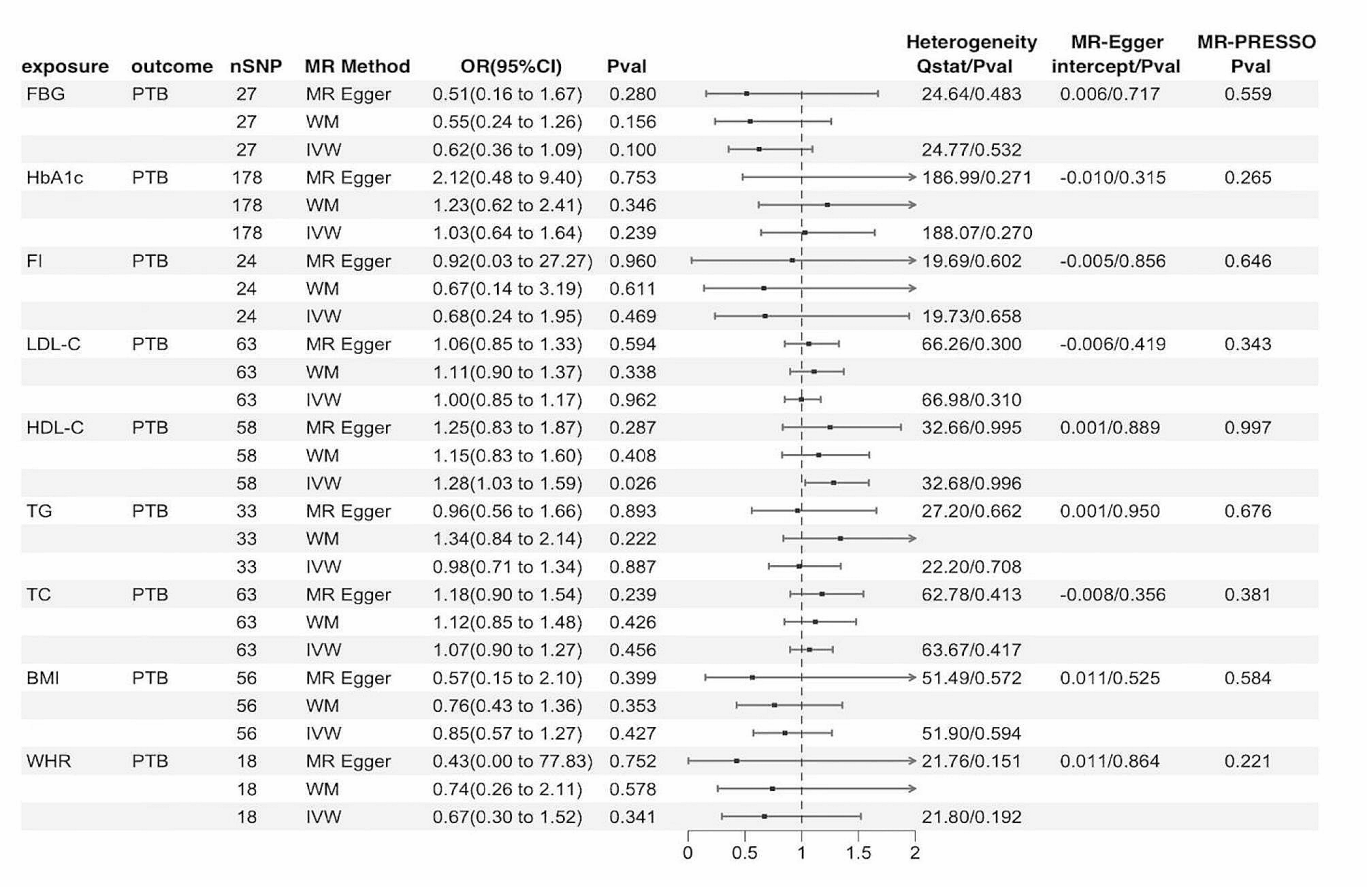
MR results for the causal effect of metabolic factors and obesity on PTB. PTB, pulmonary tuberculosis; FBG, fasting blood glucose; HbA1c, glycosylated hemoglobin; FI, fasting insulin; LDL-C, low-density lipoprotein cholesterol; HDL-C, high-density lipoprotein cholesterol; TG, triglycerides; TC, total cholesterol; BMI, body mass index; WHR, waist-to-hip ratio; SNPs, single-nucleotide polymorphisms; MR, Mendelian randomization; CI, confidence internal; OR, odds ratio; IVW, inverse variance-weighted; WM, weighted median; P value for heterogeneity based on Cochran’s Q statistic for IVW, and Rücker’s Q for MR‒Egger

Figure 3 demonstrates the MR estimates of metabolic factors and obesity on PTB. Genetic predisposition to HDL-C was associated with an increased risk of PTB (OR = 1.28, 95% CI: 1.03–1.59, *P* = 0.026; Fig. 3, Table S8, Figure S7 in the Supplement). Evidence that other metabolic factors and obesity had any effect on PTB was not identified by IVW, which was similar to the results of MR‒Egger and weighted median analyses (Fig. 3). There was no evidence of any directional pleiotropy or heterogeneity detected by Cochran’s Q test and MR‒Egger regression intercepts (Table S4-12, Figure S3-11 in the Supplement).

### Causal effect of PTB on metabolic factors and obesity

**Fig. 4:**
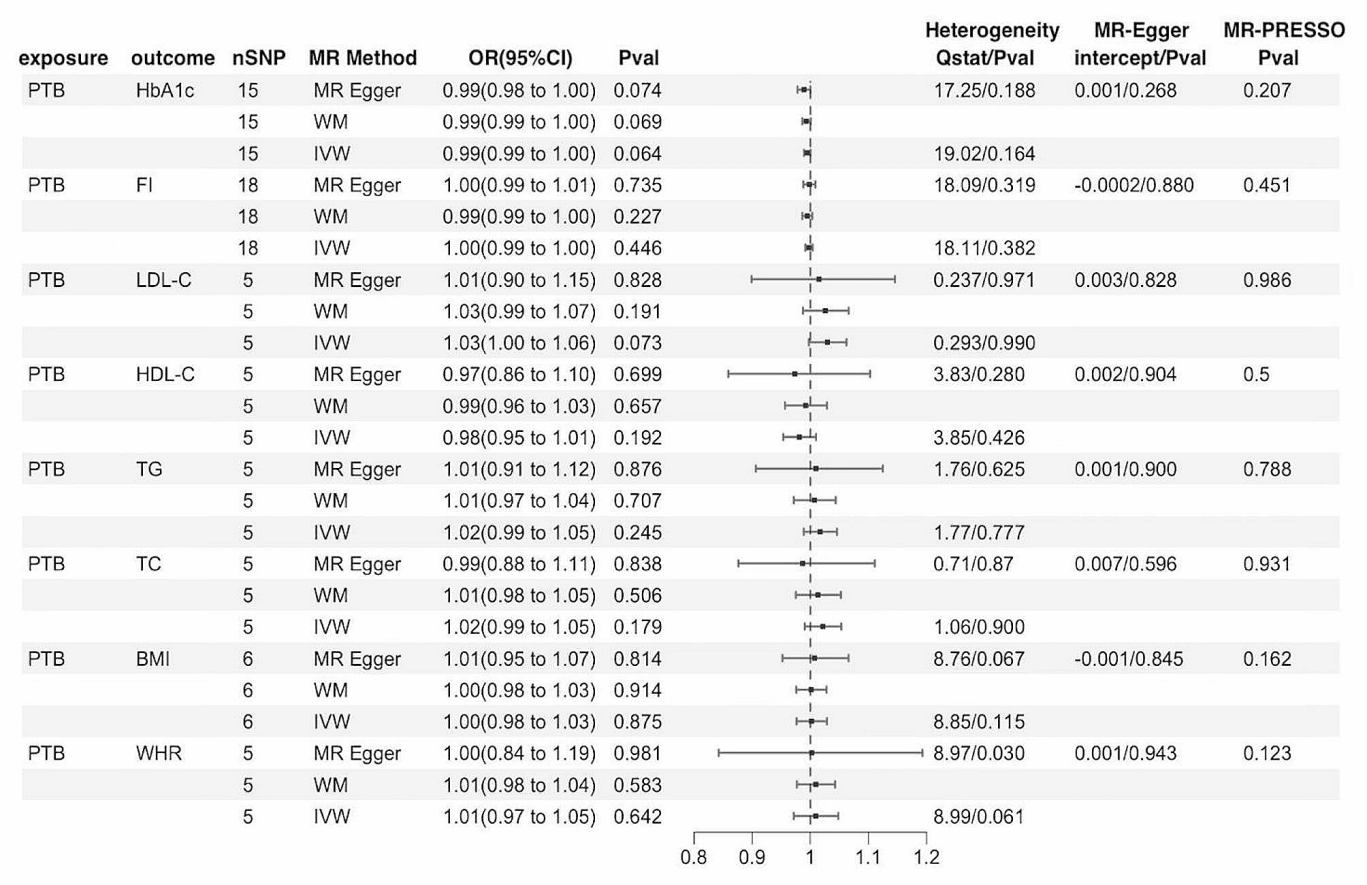
MR results for the causal effect of PTB on metabolic factors and obesity. PTB, pulmonary tuberculosis; FBG, fasting blood glucose; HbA1c, glycosylated hemoglobin; FI, fasting insulin; LDL-C, low-density lipoprotein cholesterol; HDL-C, high-density lipoprotein cholesterol; TG, triglycerides; TC, total cholesterol; BMI, body mass index; WHR, waist-to-hip ratio; SNPs, single-nucleotide polymorphisms; MR, Mendelian randomization; CI, confidence interval; OR, odds ratio; IVW, inverse variance-weighted; WM, weighted median; P value for heterogeneity based on Cochran’s Q statistic for IVW and Rücker’s Q for MR‒Egger

Figure 4 demonstrates the MR estimates of PTB on metabolic factors and obesity. To assess reverse causal effects, we extracted 20 SNPs strongly and independently associated with PTB at a significance of *P* <1e − 5. No eligible SNPs were identified between PTB and FBG. There was no statistically significant genetic risk association of PTB for metabolic factors and obesity in any analyses (Fig. 4).MR-PRESSO global test and the MR-Egger intercept test showed no evidence for horizontal pleiotropy and Cochran Q tests did not detect heterogeneity (Fig. 4, Table S13-20, Figure S12-19 in the Supplement).

## Discussion

Based on large-scale GWAS data from MR analyses, our study provided strong evidence that genetic susceptibility to T1DM was associated with an increased risk of PTB, although reverse causality was insufficiently supported. We discovered HDL-C was correlated with the risk of PTB. There was no evidence of an association between other relevant clinical traits of T1DM and PTB.

Our study corroborated that the genetic predisposition to T1DM was associated with an increased risk of PTB, which is consistent with previous studies. Animal studies have indicated an increased susceptibility to Mtb infection in T1DM rats [41]. Several previous studies have revealed significant heterogeneity in diabetes populations with PTB concerning age, sex, ethnicity, and socioeconomic status, among others [42]. Nevertheless, it seemed that T1DM remained correlated with PTB after adjusting for potential confounders. A cross-sectional study of children and youth with T1DM indicated that the prevalence of latent tuberculosis was 14.9%, with females slightly higher than males (*P* > 0.05), and that the duration of T1DM and age at diagnosis had no significant effect (*P* > 0.05) [43]. Another population-based cohort study showed that patients with T1DM were at an increased risk of tuberculosis, which was higher in men than in women (4.62 vs. 3.59), and in adults than in children (4.06 vs. 3.37), but not significantly [44]. Nonetheless, these studies were not immune to other confounding factors, and our inability to stratify by gender, age, and other factors was a potential limitation of this study. Moreover, it has been demonstrated that impaired Interleukin-1beta (IL-1β), Interleukin-6 (IL-6), and Interferon gamma (IFN-γ) production in patients with T1DM may lead to increased susceptibility to tuberculosis [45, 46]. Thus, immune dysfunction may have a significant role in tuberculosis susceptibility. As alveolar macrophages perform a cardinal function in Mtb infection and replication, IFN-γ determines macrophage activation [47, 48]. IL-1β induces eicosanoids to promote bacterial control and limits type 1 IFN-γ production, which reduces the effect of macrophages and increases tuberculosis susceptibility in patients with T1DM [49]. Regarding the opposite direction, our MR analyses revealed no evidence to support a causal effect of PTB on T1DM. Despite the studies that have reported a greater risk of diabetes in patients with PTB, evidence is scarce [50]. Banyai has proposed from animal experiments that Mtb infection can promote necrosis and atrophy of the pancreas to affect diabetes [51]. Given that T2DM comprises 95% of diabetes, patients in a majority of studies appeared to be T2DM, with the TB-T1DM association currently understudied. Moreover, the results may be attributed to transient hyperglycemia as a result of febrile manifestations of PTB [52]. A prospective cohort study indicated that PTB could promote transient glycemia, without conclusively demonstrating the promotion of chronic glycemic abnormalities [53].

The study also explored the correlation between PTB and relevant metabolic characteristics of T1DM. Our purpose was to confirm the influencing factors further by investigating the causal association between glycemic traits and PTB. However, we did not identify a causal correlation between PTB and FBG, HbA1c, and FI, which was different from some previous observational studies [15, 54]. The difference may be explained as follows: (1) The results obtained from observational studies may be affected by bias, such as confounding factors or reverse causality. (2) Different studies have reached inconsistent conclusions. For example, the cohort study by Pin-Hui Lee et al. indicated that poor glycemic control had a significantly higher hazard of tuberculosis [15]. Conversely, the opposite conclusion was reported in a Danish population-based case‒control study, which did not demonstrate a significant correlation between glycemic traits and tuberculosis [55].

In addition, we used several mediators associated with lipid metabolism, including HDL-C, LDL-C, TG, and TC. We found that elevated HDL-C levels increased the risk of tuberculosis. Lipid metabolism, as one of the essential metabolic pathways, can serve as a secondary source of nutrients for PTB infection, favoring growth and multiplication against Mtb. Mtb induces macrophage differentiation into lipid-loaded foam cells and acquires a dormant-like phenotype [56]. Mtb infection forms granulomas whose core consists of infected macrophages. Progression of granuloma infection is frequently accompanied by dysregulation of lipid metabolism [57]. It has been suggested that HDL-C enhances Mtb infection in macrophages [58]. Interestingly, HDL-C plays a dual role in the prevention and regulation of PTB infection. It has been demonstrated that HDL levels are reduced after infection with tuberculosis [16]. This is probably due to the capacity of HDL to inhibit the production of tumor necrosis factor-alpha (TNF-α), which is critical in the immune defense against TB [58]. Therefore, further research is still necessary to elucidate the role of HDL-C in different states of TB, such as uninfected, initial infection, asymptomatic state, and active disease.

However, a causal relationship between obesity and tuberculosis was not identified, which differed from several previous studies. A systematic review showed a negative correlation between BMI and the incidence of tuberculosis [59]. However, results from observational studies may be subject to confounders and reverse causality. For instance, it has been demonstrated that there was a significant correlation between BMI and anemia in patients with PTB, while anaemia as a risk factor for PTB was excluded as a confounder in this study [60, 61]. Consequently, the MR analysis draws more robust and substantiated conclusions without these issues.

To our knowledge, the study is the first to reveal a causal association between T1DM and PTB using bidirectional MR analysis. Nevertheless, our study has some limitations as well. First, most of the statistics in GWAS were from individuals of European ancestry, which may have raised concerns about the generalizability of the findings to other populations. Ideally, we would repeat this association analysis in large GWAS data from regions with high PTB prevalence (e.g., Africa and South Asia). However, large populations with relevant genomic data are not yet available for further study. Second, despite our efforts to minimize pleiotropy, it was unlikely that all instances of pleiotropy would be eliminated in an MR analysis, which could have biased our results. Third, a potential limitation of the study was the inability to stratify the analyses based on gender, age, and duration of T1DM, among other significant variables.

## Conclusion

In conclusion, our bidirectional MR study provided strong evidence of the causal relationship between T1DM, HDL-C, and PTB, while the reverse direction indicated no causal associations. Thus, prevention strategies for PTB should include treatment of T1DM and control of HDL-C levels, providing an essential basis for prevention and comanagement of concurrent T1DM and PTB in clinical practice.

### Electronic supplementary material

Below is the link to the electronic supplementary material.

Supplementary Material 1

## Data Availability

The original contributions presented in the study are included in the article/Supplementary Material. Further inquiries can be directed to the corresponding author.

## Abbreviations

T1DM: Type 1 diabetes mellitus
PTB: Pulmonary tuberculosis
Mtb: Mycobacterium tuberculosis
FBG: Fasting blood glucose
FI: Fasting insulin
HbA1c: Glycosylated hemoglobin
TG: Triglycerides
TC: Total cholesterol
LDL-C: Low-density lipoprotein cholesterol
HDL-C: High-density lipoprotein cholesterol
BMI: Body mass index
WHR: Waist-to-hip ratio
MR: Mendelian randomization
SNPs: Single nucleotide polymorphisms
IVW: Inverse variance weighting method
WM: Weighted median
MR-PRESSO: MR-pleiotropy residual sum and outlier
IVs: Instrumental variables
OR: Odds ratio
CI: Confidence interval
