# Supplementary material for: Associations between type 1 diabetes and pulmonary tuberculosis: a bidirectional mendelian randomization study
Source: Diabetol Metab Syndr. 2024 Mar 5;16:60. doi: 10.1186/s13098-024-01296-x (PMC10913601; doi:10.1186/s13098-024-01296-x)
Supplement: Supplementary file 1 — Supplementary Material 1 [file 13098_2024_1296_MOESM1_ESM.pdf]

Table S1. Excluded SNPs of Potential confounders.

Exposures	Exclude SNPs	Traits
T1DM	rs4490209	Hemoglobin concentration; Mean corpuscular hemoglobin; Mean corpuscular volume
	rs12464462	Mean corpuscular hemoglobin; Mean corpuscular volume
	rs2429557	Mean corpuscular hemoglobin concentration
	rs9385401	Hemoglobin concentration
	rs3802214	Mean corpuscular hemoglobin
	rs1701704	Alcohol intake frequency
	rs3184504	Hemoglobin Hb; Ever smoked; Smoking status: previous;Past tobacco smoking;
	rs35327136	Hemoglobin concentration; Alcohol intake frequency
	rs601338	Alcohol intake frequency; Alcohol intake versus 10 years previously
FBG	rs1260326	Hemoglobin concentration; Mean corpuscular hemoglobin concentration; Alcohol consumption; Alcohol consumption in current drinkers; Alcohol intake frequency; Alcohol intake versus 10 years previously
	rs11715915	Mean corpuscular hemoglobin; Mean corpuscular volume
	rs174576	Hemoglobin concentration; Mean corpuscular volume
HAb1c	rs695238	Alcohol intake frequency; Alcohol intake versus 10 years previously; Exposure to tobacco smoke at home
	rs1991431	Mean corpuscular hemoglobin
	rs6902771	Alcohol dependence; Alcohol dependence; Alcoholism
	rs3734537	Hemoglobin concentration; Mean corpuscular hemoglobin
	rs3757230	Mean corpuscular hemoglobin
	rs9379084	Hemoglobin concentration
	rs1490384	Hemoglobin concentration
	rs28846812	Hemoglobin concentration
	rs71276225	Hemoglobin concentration
	rs12677543	Mean corpuscular hemoglobin
	rs28446901	Hemoglobin concentration
	rs7870193	Hemoglobin concentration
	rs3138499	Pack years adult smoking as proportion of life span exposed to smoking; Pack years of smoking preview only
	rs12413039	Nicotine dependence smoking smoking cessation
	rs7124681	Alcohol intake frequency
	rs588713	Alcohol usually taken with meals
	rs7952436	Mean corpuscular hemoglobin
	rs7968682	Alcohol intake frequency
	rs1701704	Alcohol intake frequency
	rs1046276	Mean corpuscular hemoglobin
	rs3790076	Past tobacco smoking
	rs11645303	Hemoglobin concentration; Mean corpuscular hemoglobin concentration
	rs78378222	Mean corpuscular hemoglobin
	rs73984689	Hemoglobin concentration; Mean corpuscular hemoglobin; Alcohol intake

		frequency
	rs1680331	Alcohol intake frequency
	rs12721051	Mean corpuscular hemoglobin concentration
	rs72976986	Mean corpuscular hemoglobin
	rs3761123	Current tobacco smoking; Smoking status: current
	rs6088619	Difficulty not smoking for 1 day
	rs45577732	Current tobacco smoking; Pack years adult smoking as proportion of life span exposed to smoking; Pack years of smoking preview only; Smoking status: current
	rs11909473	Hemoglobin concentration
	rs41281623	Mean corpuscular hemoglobin
FI	rs13389219	Hemoglobin concentration; Mean corpuscular hemoglobin concentration
	rs1260326	Hemoglobin concentration; Mean corpuscular hemoglobin concentration; Alcohol consumption; Alcohol consumption in current drinkers; Alcohol intake frequency; Alcohol intake versus 10 years previously
	rs35000407	Mean corpuscular hemoglobin concentration
	rs62271373	Alcohol intake frequency
	rs10865959	Alcohol intake frequency; Alcohol intake versus 10 years previously
	rs3775380	Hemoglobin concentration
	rs9884482	Alcohol intake frequency
	rs459193	Hemoglobin concentration
	rs1474696	Hemoglobin concentration
	rs116141873	Mean corpuscular hemoglobin
	rs6905288	Hemoglobin concentration
	rs75179845	Hemoglobin concentration; Mean corpuscular hemoglobin; Mean corpuscular hemoglobin concentration
	rs1351394	Alcohol intake frequency
LDL-C	rs10903129	Mean corpuscular hemoglobin concentration; Nicotine dependence smoking ever smokers versus never smokers
	rs10195252	Hemoglobin concentration
	rs17404153	Mean corpuscular hemoglobin
	rs9875338	Mean corpuscular hemoglobin concentration
	rs3757354	Mean corpuscular hemoglobin
	rs1408272	Hemoglobin concentration;Mean corpuscular hemoglobin; Mean corpuscular hemoglobin concentration; Hemoglobin Hb; Mean cell hemoglobin MCH; Mean cell hemoglobin MCH Mean cell volume Hemoglobin Hgb Mean cell
	rs13206249	Mean corpuscular hemoglobin
	rs9987289	Hemoglobin concentration
	rs2954029	Mean corpuscular hemoglobin
	rs579459	Hemoglobin concentration; Hemoglobin Hb
	rs174583	Hemoglobin concentration
	rs964184	Mean corpuscular hemoglobin concentration
	rs3184504	Hemoglobin concentration; Ever smoked; Past tobacco smoking; Smoking status:

		previous
	rs2886232	Hemoglobin concentration
	rs676388	Alcohol intake frequency; Alcohol intake versus 10 years previously
	rs6065311	Hemoglobin concentration
	rs1800961	Hemoglobin concentration
HDL-C	rs4660293	Mean corpuscular hemoglobin; Alcohol intake versus 10 years previously
	rs1047891	Mean corpuscular hemoglobin
	rs7607980	Hemoglobin concentration
	rs2013208	Alcohol intake frequency; Alcohol intake versus 10 years previously
	rs3822072	Hemoglobin concentration
	rs13107325	Hemoglobin concentration; Alcohol intake frequency
	rs998584	Hemoglobin concentration
	rs1936800	Hemoglobin concentration
	rs3861397	Mean corpuscular hemoglobin
	rs1980493	Hemoglobin concentration
	rs17145738	Alcohol intake frequency
	rs4240624	Hemoglobin concentration
	rs2293889	Hemoglobin concentration
	rs10808546	Mean corpuscular hemoglobin
	rs10761771	Alcohol usually taken with meals
	rs970548	Mean corpuscular hemoglobin
	rs964184	Mean corpuscular hemoglobin concentration
	rs102275	Hemoglobin concentration
	rs499974	Hemoglobin concentration
	rs12801636	Mean corpuscular hemoglobin
	rs11065987	Hemoglobin concentration; Ever smoked; Past tobacco smoking; Smoking status: previous
	rs10468017	Hemoglobin Hb
	rs492571	Hemoglobin concentration; Mean corpuscular hemoglobin
	rs16942887	Hemoglobin concentration
	rs1877031	Mean corpuscular hemoglobin
	rs4969178	Hemoglobin Hb
	rs6567160	Alcohol intake frequency
	rs4465830	Mean corpuscular hemoglobin
	rs181360	Mean corpuscular hemoglobin
TG	rs17513135	Alcohol intake versus 10 years previously; Mean corpuscular hemoglobin
	rs1321257	Hemoglobin concentration
	rs1260326	Hemoglobin concentration; Alcohol consumption; Alcohol intake frequency; Alcohol intake versus 10 years previously
	rs13389219	Hemoglobin concentration; Mean corpuscular hemoglobin concentration
	rs442177	Hemoglobin concentration
	rs9686661	Hemoglobin concentration; Mean corpuscular hemoglobin concentration
	rs634869	Mean corpuscular hemoglobin

	rs2239520	Hemoglobin concentration; Mean corpuscular hemoglobin concentration
	rs998584	Hemoglobin concentration
	rs719726	Hemoglobin concentration
	rs2247056	Mean corpuscular hemoglobin; HIV 1 control; HIV 1 control viral load at set point
	rs11974409	Alcohol intake frequency
	rs12678919	Mean corpuscular hemoglobin concentration
	rs2954022	Mean corpuscular hemoglobin
	rs1832007	Hemoglobin concentration
	rs10761762	Alcohol usually taken with meals
	rs7350481	Mean corpuscular hemoglobin concentration
	rs588136	Hemoglobin concentration
	rs16948098	Hemoglobin concentration
	rs749671	Mean corpuscular hemoglobin; Alcohol intake frequency
	rs439401	Mean corpuscular hemoglobin concentration
	rs4810479	Mean corpuscular hemoglobin
TC	rs11802413	Mean corpuscular hemoglobin concentration; Nicotine dependence smoking ever smokers versus never smokers
	rs2287623	Mean corpuscular hemoglobin concentration
	rs780093	Hemoglobin concentration; Alcohol intake frequency; Alcohol intake versus 10 years previously
	rs7616006	Mean corpuscular hemoglobin concentration
	rs3757354	Mean corpuscular hemoglobin
	rs9272775	Hemoglobin concentration
	rs1800562	Hemoglobin concentration; Mean corpuscular hemoglobin
	rs9391858	HIV 1 control; HIV 1 disease progression
	rs9376090	Hemoglobin concentration; Mean corpuscular hemoglobin
	rs9987289	Hemoglobin concentration
	rs2954029	Mean corpuscular hemoglobin
	rs579459	Hemoglobin concentration
	rs10900221	Mean corpuscular hemoglobin
	rs1535	Hemoglobin concentration
	rs4752805	Hemoglobin concentration
	rs964184	Mean corpuscular hemoglobin concentration
	rs3184504	Hemoglobin concentration; Ever smoked; Past tobacco smoking; Smoking status: previous
	rs2244608	Hemoglobin concentration
	rs10468017	Hemoglobin Hb
	rs2886232	Hemoglobin concentration
	rs1800961	Hemoglobin concentration
	rs2235367	Hemoglobin concentration
	rs181360	Mean corpuscular hemoglobin
BMI	rs3101336	Alcohol intake versus 10 years previously

	rs6656785	Past tobacco smoking
	rs13021737	Past tobacco smoking
	rs13078960	Ever smoked
	rs13107325	Hemoglobin concentration; Alcohol intake frequency
	rs3817334	Alcohol intake frequency
	rs11030104	Ever smoked
	rs3888190	Mean corpuscular hemoglobin; Alcohol intake frequency
	rs4889606	Mean corpuscular hemoglobin; Alcohol intake frequency
	rs1558902	Alcohol intake frequency
	rs1808579	Alcohol intake frequency
	rs6567160	Alcohol intake frequency
WHR	rs1128249	Hemoglobin concentration
	rs2972164	Mean corpuscular hemoglobin concentration
	rs17451107	Mean corpuscular hemoglobin concentration
	rs459193	Hemoglobin concentration
	rs9491696	Hemoglobin concentration
	rs998584	Hemoglobin concentration
	rs17109256	Alcohol intake frequency
	rs1121980	Alcohol intake frequency
	rs11663816	Alcohol intake frequency
	rs3786897	Mean corpuscular hemoglobin

T1DM, type 1 diabetes; FBG, fasting blood glucose; HbA1c, glycosylated hemoglobin; FI, fasting insulin; LDL-C, low-density lipoprotein cholesterol; HDL-C, high-density lipoprotein cholesterol; TG, triglycerides; TC, total cholesterol; BMI, body mass index; WHR, waist-to-hip ratio.

Table S1. Published associations of T1DM on PTB

SNP	Exposure	EA	OA	GX	GX(SE)	pval	F	GY	GY(SE)	pval
rs10224046	T1DM	G	T	0.086	0.015	2.71E-08	31	0.026	0.035	0.460
rs10275896	T1DM	C	T	-0.121	0.017	2.60E-13	53	0.084	0.043	0.050
rs1050979	T1DM	G	A	0.106	0.014	5.65E-14	56	-0.012	0.034	0.710
rs10751776	T1DM	C	A	0.078	0.014	2.67E-08	31	0.007	0.034	0.845
rs10801128	T1DM	G	A	0.096	0.016	8.98E-10	38	0.041	0.039	0.288
rs10844597	T1DM	A	G	-0.090	0.014	1.20E-10	41	-0.047	0.034	0.159
rs11203203	T1DM	A	G	0.144	0.014	1.81E-23	100	-0.024	0.037	0.517
rs112733823	T1DM	T	C	0.383	0.020	7.42E-81	363	0.065	0.041	0.112
rs114378220	T1DM	T	C	0.178	0.030	5.11E-09	34	-0.133	0.072	0.067
rs12128789	T1DM	C	T	0.127	0.022	3.73E-09	35	0.070	0.053	0.182
rs12257077	T1DM	T	C	0.231	0.037	3.91E-10	39	-0.029	0.128	0.823
rs12644686	T1DM	G	C	-0.108	0.019	2.44E-08	31	0.069	0.039	0.076
rs12742756	T1DM	G	A	-0.083	0.015	3.54E-08	30	0.036	0.034	0.284
rs12927355	T1DM	T	C	-0.204	0.015	4.41E-41	180	0.055	0.037	0.133
rs13147049	T1DM	G	A	-0.110	0.015	8.92E-14	56	-0.016	0.034	0.632
rs13259300	T1DM	C	A	-0.092	0.015	3.28E-10	39	-0.031	0.034	0.368
rs1350275	T1DM	G	T	-0.094	0.015	8.86E-10	38	-0.014	0.035	0.693
rs1574285	T1DM	T	G	-0.127	0.014	4.27E-19	80	-0.061	0.034	0.073
rs1611236	T1DM	A	G	-0.260	0.017	1.83E-50	223	-0.063	0.040	0.118
rs17106304	T1DM	G	C	0.115	0.015	6.83E-15	61	-0.019	0.035	0.593
rs17323934	T1DM	G	C	-0.130	0.017	1.26E-14	59	0.091	0.043	0.036
rs17623914	T1DM	C	T	-0.135	0.023	7.97E-09	33	-0.073	0.065	0.263
rs1881146	T1DM	T	A	-0.095	0.017	4.57E-08	30	0.012	0.038	0.751
rs1947178	T1DM	G	A	-0.103	0.017	1.67E-09	36	-0.094	0.047	0.047
rs202535	T1DM	A	C	-0.141	0.018	1.79E-14	59	0.029	0.041	0.480
rs2111485	T1DM	G	A	0.128	0.014	1.05E-18	78	0.002	0.034	0.957
rs2188962	T1DM	T	C	0.079	0.014	1.73E-08	32	-0.064	0.036	0.078
rs229527	T1DM	A	C	0.104	0.014	1.82E-13	54	-0.011	0.034	0.740
rs231972	T1DM	C	A	0.171	0.021	5.12E-16	66	0.037	0.059	0.527
rs238265	T1DM	G	T	-0.091	0.015	2.08E-09	36	-0.017	0.036	0.636
rs238873	T1DM	G	A	0.793	0.053	4.30E-50	221	-0.160	0.134	0.233
rs2493411	T1DM	C	T	0.127	0.022	1.28E-08	32	0.010	0.045	0.826
rs2543537	T1DM	T	C	-0.083	0.014	5.59E-09	34	0.024	0.034	0.471
rs2611211	T1DM	T	C	-0.144	0.019	1.39E-14	59	-0.031	0.039	0.431
rs28752526	T1DM	G	A	0.630	0.016	1.00E-200	1570	0.121	0.036	0.001
rs3024493	T1DM	A	C	-0.164	0.020	7.26E-17	70	0.087	0.046	0.061
rs3087243	T1DM	A	G	-0.199	0.014	1.16E-44	197	-0.028	0.036	0.440
rs3135348	T1DM	G	A	1.048	0.017	1.00E-200	3610	0.075	0.034	0.026
rs34536443	T1DM	C	G	-0.385	0.039	1.47E-23	100	-0.106	0.098	0.280

rs34593439	T1DM	A	G	-0.218	0.024	1.54E-19	82	0.011	0.050	0.829
rs41295159	T1DM	G	C	-0.700	0.090	9.11E-15	60	0.142	0.410	0.729
rs4548024	T1DM	C	T	-0.096	0.017	9.95E-09	33	-0.064	0.041	0.117
rs4820827	T1DM	C	T	-0.130	0.014	1.01E-19	83	0.021	0.036	0.566
rs55893453	T1DM	G	A	0.095	0.017	4.63E-08	30	-0.016	0.046	0.728
rs55993634	T1DM	G	C	0.219	0.024	2.29E-19	81	0.015	0.060	0.803
rs56994090	T1DM	C	T	-0.134	0.015	3.60E-20	85	-0.036	0.034	0.283
rs57209021	T1DM	T	C	0.101	0.018	3.73E-08	30	-0.037	0.041	0.366
rs574384	T1DM	A	C	-0.134	0.024	2.20E-08	31	-0.052	0.051	0.303
rs607703	T1DM	T	C	0.092	0.014	1.17E-10	42	-0.022	0.034	0.522
rs61759532	T1DM	T	C	0.118	0.019	1.91E-10	41	0.076	0.043	0.078
rs61839660	T1DM	T	C	-0.357	0.026	5.25E-43	189	0.133	0.085	0.116
rs6434435	T1DM	A	G	-0.123	0.019	1.23E-10	41	0.073	0.055	0.183
rs663743	T1DM	A	G	-0.100	0.015	3.50E-11	44	0.035	0.035	0.318
rs6679677	T1DM	A	C	0.642	0.021	1.00E-200	939	0.045	0.048	0.348
rs689	T1DM	T	A	0.712	0.019	1.00E-200	1477	0.043	0.041	0.297
rs6908236	T1DM	C	A	0.166	0.014	6.31E-32	138	0.051	0.034	0.130
rs6908626	T1DM	T	G	0.203	0.019	6.14E-28	120	-0.119	0.053	0.024
rs7068821	T1DM	T	G	-0.165	0.016	5.07E-24	102	-0.048	0.040	0.228
rs7130222	T1DM	G	T	-0.092	0.016	1.30E-08	32	-0.021	0.038	0.575
rs722988	T1DM	C	T	0.083	0.014	9.78E-09	33	-0.014	0.036	0.706
rs7237497	T1DM	C	T	-0.220	0.019	2.71E-32	140	-0.015	0.048	0.754
rs7301381	T1DM	C	T	-0.094	0.014	5.25E-11	43	-0.090	0.034	0.008
rs73432769	T1DM	T	C	0.335	0.046	2.34E-13	54	0.124	0.136	0.360
rs7511678	T1DM	A	G	0.094	0.017	3.09E-08	31	-0.020	0.036	0.575
rs7668577	T1DM	C	A	0.094	0.015	7.26E-10	38	0.028	0.036	0.444
rs7752257	T1DM	G	T	-0.680	0.017	1.00E-200	1678	-0.055	0.039	0.160
rs7776597	T1DM	G	A	0.244	0.036	1.82E-11	45	0.023	0.109	0.834
rs7795896	T1DM	T	C	-0.135	0.016	1.58E-16	68	-0.007	0.035	0.852
rs78325861	T1DM	G	C	-0.282	0.042	2.31E-11	45	0.150	0.092	0.104
rs8046043	T1DM	C	G	-0.085	0.015	2.49E-08	31	-0.009	0.036	0.805
rs855330	T1DM	C	T	0.111	0.017	4.89E-11	43	0.002	0.035	0.965
rs9260802	T1DM	G	A	-0.356	0.042	1.35E-17	73	0.011	0.088	0.898
rs9468618	T1DM	T	C	-0.272	0.026	5.10E-25	107	-0.040	0.073	0.579
rs9517712	T1DM	C	T	-0.102	0.016	1.06E-10	42	0.001	0.037	0.982

T1DM: type 1 diabetes. EA: effect allele. OA: other allele. EAF: EA frequency. GX: beta-exposure. GX(SE): standard error of GX. GY: beta-outcome. GY(SE): standard error of GY.

Table S3. Published associations of PTB on T1DM

SNP	Exposure	EA	OA	GX	GX(SE)	pval	F	GY	GY(SE)	pval
rs10847937	PTB	C	A	0.176	0.038	3.20E-06	22	0.015	0.018	0.389
rs117050744	PTB	C	T	-0.444	0.098	5.58E-06	21	-0.002	0.085	0.977
rs117620244	PTB	T	C	0.361	0.070	2.49E-07	27	0.004	0.058	0.949
rs118082956	PTB	G	A	0.379	0.085	7.58E-06	20	0.155	0.063	0.014
rs11926030	PTB	G	T	0.417	0.094	8.66E-06	20	-0.092	0.060	0.125
rs13434036	PTB	T	G	-0.446	0.097	4.02E-06	21	0.020	0.039	0.607
rs145653024	PTB	T	C	0.802	0.161	6.46E-07	25	-0.009	0.075	0.901
rs2027204	PTB	A	G	0.159	0.034	3.05E-06	22	0.015	0.016	0.349
rs62201868	PTB	A	G	-2.155	0.487	9.75E-06	20	0.060	0.062	0.338
rs6586520	PTB	T	C	0.155	0.034	4.32E-06	21	-0.018	0.014	0.198
rs72738736	PTB	T	G	0.157	0.035	8.09E-06	20	0.008	0.015	0.590
rs73579425	PTB	T	C	0.418	0.094	9.40E-06	20	0.039	0.061	0.521
rs75686985	PTB	A	C	-0.160	0.035	5.03E-06	21	0.041	0.016	0.008
rs79513808	PTB	T	C	0.331	0.073	5.51E-06	21	0.008	0.031	0.793

PTB: pulmonary tuberculosis. EA: effect allele. OA: other allele. EAF: EA frequency. GX: beta-exposure. GX(SE): standard error of GX. GY: beta-outcome. GY(SE): standard error of GY.

Table S4. Published associations of FBG on PTB

SNP	Exposure	EA	OA	GX	GX(SE)	pval	F	GY	GY(SE)	pval
rs10811661	FBG	C	T	-0.024	0.003	5.65E-18	73	0.014	0.048	0.770
rs10814916	FBG	C	A	0.016	0.002	2.26E-13	53	0.080	0.034	0.018
rs11195502	FBG	T	C	-0.032	0.004	1.97E-18	75	0.056	0.059	0.343
rs11558471	FBG	G	A	-0.029	0.002	7.80E-37	159	0.018	0.035	0.613
rs11603334	FBG	A	G	-0.019	0.003	1.12E-11	46	-0.026	0.040	0.513
rs11607883	FBG	A	G	-0.021	0.002	6.32E-24	100	0.049	0.034	0.143
rs11619319	FBG	G	A	0.020	0.002	1.33E-15	69	0.011	0.039	0.768
rs11708067	FBG	G	A	-0.023	0.003	1.30E-18	78	-0.039	0.045	0.382
rs1280	FBG	C	T	-0.026	0.003	8.56E-18	70	0.062	0.050	0.214
rs12888855	FBG	A	C	-0.016	0.003	5.04E-10	41	0.016	0.043	0.715
rs16913693	FBG	G	T	-0.043	0.007	3.51E-11	42	0.125	0.110	0.256
rs17168486	FBG	T	C	0.031	0.003	3.17E-28	123	-0.037	0.042	0.382
rs2191349	FBG	T	G	0.029	0.002	1.28E-42	191	-0.018	0.034	0.603
rs3829109	FBG	A	G	-0.017	0.003	1.13E-10	40	0.022	0.035	0.539
rs4502156	FBG	C	T	-0.022	0.002	1.38E-25	110	0.085	0.034	0.013
rs4869272	FBG	T	C	0.018	0.002	1.02E-15	67	-0.013	0.036	0.710
rs560887	FBG	C	T	0.071	0.003	1.40E-178	807	-0.043	0.037	0.240
rs6072275	FBG	A	G	0.016	0.003	1.66E-08	33	-0.008	0.039	0.845
rs6113722	FBG	A	G	-0.035	0.005	2.49E-11	44	-0.022	0.089	0.808
rs6943153	FBG	C	T	-0.015	0.002	1.63E-12	46	0.023	0.034	0.505
rs6975024	FBG	C	T	0.061	0.003	2.88E-99	442	0.040	0.055	0.466
rs749067	FBG	C	T	-0.017	0.002	6.12E-15	60	-0.010	0.036	0.783
rs7651090	FBG	G	A	0.013	0.002	1.75E-08	32	0.006	0.036	0.879
rs7903146	FBG	T	C	0.022	0.002	2.71E-20	84	-0.020	0.042	0.633
rs882020	FBG	T	C	0.021	0.003	3.04E-12	49	-0.056	0.045	0.212
rs9368222	FBG	A	C	0.014	0.002	1.00E-09	37	0.034	0.036	0.341
rs983309	FBG	G	T	-0.026	0.003	6.29E-15	62	-0.051	0.045	0.260

FBG: fasting blood glucose. EA: effect allele. OA: other allele. EAF: EA frequency. GX: beta-exposure. GX(SE): standard error of GX. GY: beta-outcome. GY(SE): standard error of GY.

Table S5. Published associations of HAb1c on PTB

SNP	Exposure	EA	OA	GX	GX(SE)	pval	F	GY	GY(SE)	pval
rs10037493	HAb1c	T	C	0.017	0.001	9.30E-31	133	0.059	0.034	0.084
rs10141786	HAb1c	G	A	-0.011	0.002	1.10E-12	51	0.046	0.035	0.191
rs1026477	HAb1c	A	G	-0.008	0.001	1.70E-08	32	-0.025	0.034	0.461
rs10283100	HAb1c	G	A	0.024	0.003	3.00E-13	53	-0.104	0.065	0.108
rs10455823	HAb1c	G	A	-0.023	0.004	1.60E-10	41	0.073	0.137	0.597
rs1046934	HAb1c	C	A	0.010	0.002	4.50E-10	39	-0.001	0.034	0.968
rs10475282	HAb1c	A	G	-0.010	0.002	2.10E-09	36	0.005	0.044	0.909
rs10476063	HAb1c	A	G	-0.012	0.002	6.10E-15	61	0.009	0.035	0.792
rs10490551	HAb1c	A	T	0.012	0.002	4.50E-13	52	-0.034	0.037	0.366
rs10771750	HAb1c	C	A	0.010	0.001	5.10E-11	43	-0.024	0.034	0.486
rs10848835	HAb1c	C	G	0.016	0.003	5.90E-11	43	0.002	0.067	0.980
rs10888385	HAb1c	C	T	-0.012	0.002	1.90E-16	68	-0.022	0.034	0.508
rs10978435	HAb1c	C	T	-0.009	0.002	4.80E-08	30	0.032	0.036	0.361
rs11049381	HAb1c	A	G	0.011	0.002	3.80E-14	57	-0.046	0.034	0.170
rs11085744	HAb1c	T	C	-0.012	0.002	3.30E-15	62	0.020	0.034	0.565
rs11118683	HAb1c	T	C	0.009	0.002	5.80E-09	34	-0.025	0.034	0.462
rs11170395	HAb1c	G	A	0.015	0.003	1.40E-08	32	0.015	0.055	0.793
rs112723784	HAb1c	C	T	0.009	0.002	3.10E-08	31	0.047	0.039	0.220
rs113252144	HAb1c	T	C	0.021	0.003	5.90E-14	56	0.100	0.063	0.115
rs113898003	HAb1c	C	T	-0.018	0.002	8.40E-28	119	-0.004	0.039	0.921
rs115152027	HAb1c	G	C	-0.019	0.004	3.40E-08	30	-0.069	0.101	0.496
rs11621587	HAb1c	C	G	0.017	0.002	6.10E-19	79	0.021	0.049	0.669
rs11645016	HAb1c	T	C	-0.009	0.002	2.50E-09	36	-0.027	0.035	0.431
rs11651123	HAb1c	A	G	-0.013	0.001	2.70E-18	76	0.020	0.034	0.568
rs11664776	HAb1c	C	T	-0.008	0.002	2.60E-08	31	-0.019	0.035	0.591
rs11707746	HAb1c	C	T	-0.011	0.002	1.40E-10	41	-0.051	0.039	0.188
rs11732618	HAb1c	A	G	0.009	0.002	2.40E-09	36	-0.016	0.037	0.670
rs11733064	HAb1c	G	C	-0.010	0.002	4.80E-10	39	0.061	0.038	0.108
rs117372259	HAb1c	G	A	0.025	0.004	2.90E-08	31	-0.070	0.121	0.564
rs11765149	HAb1c	C	G	-0.016	0.003	1.40E-08	32	-0.022	0.052	0.674
rs11938781	HAb1c	C	T	-0.027	0.002	2.40E-41	181	-0.019	0.055	0.730
rs11967850	HAb1c	T	C	-0.011	0.002	6.20E-09	34	0.024	0.049	0.622
rs11984666	HAb1c	A	C	-0.016	0.002	3.30E-18	76	0.040	0.045	0.368
rs12036042	HAb1c	C	T	0.012	0.002	6.40E-11	43	-0.107	0.041	0.010
rs12563643	HAb1c	G	C	0.015	0.002	6.60E-16	65	-0.065	0.042	0.124
rs12571363	HAb1c	A	C	-0.018	0.002	2.70E-13	53	-0.096	0.072	0.184
rs12631704	HAb1c	G	A	-0.014	0.002	1.00E-15	64	-0.015	0.042	0.717
rs12684650	HAb1c	T	C	-0.015	0.002	1.00E-20	87	-0.106	0.036	0.003
rs12698403	HAb1c	A	G	-0.013	0.002	3.50E-17	71	-0.021	0.034	0.527

rs12894780	HAb1c	C	T	-0.017	0.002	4.40E-14	57	0.136	0.051	0.007
rs13044144	HAb1c	C	G	0.014	0.002	1.90E-17	72	-0.022	0.040	0.576
rs13044825	HAb1c	C	G	0.012	0.002	4.80E-13	52	0.043	0.034	0.206
rs13073544	HAb1c	C	G	-0.009	0.002	4.70E-08	30	-0.018	0.045	0.696
rs13101469	HAb1c	T	A	-0.014	0.002	2.60E-14	58	-0.059	0.045	0.190
rs13229167	HAb1c	A	G	-0.013	0.002	1.70E-14	59	-0.033	0.039	0.401
rs13245206	HAb1c	A	G	-0.009	0.002	4.60E-09	34	-0.013	0.034	0.709
rs13417268	HAb1c	G	C	0.011	0.002	1.50E-09	37	-0.018	0.039	0.644
rs140988776	HAb1c	T	C	-0.021	0.003	5.20E-13	52	-0.014	0.085	0.874
rs147110934	HAb1c	T	G	-0.039	0.005	1.10E-15	64	0.222	0.223	0.320
rs1474647	HAb1c	T	C	-0.012	0.001	5.10E-15	61	0.028	0.034	0.405
rs1516890	HAb1c	A	G	-0.009	0.002	3.60E-10	39	0.017	0.034	0.606
rs1530555	HAb1c	T	C	0.011	0.002	1.70E-13	54	0.013	0.034	0.715
rs1555211	HAb1c	T	C	0.010	0.002	4.80E-09	34	-0.038	0.038	0.322
rs16854110	HAb1c	G	C	-0.019	0.002	4.00E-19	80	0.010	0.042	0.809
rs17099139	HAb1c	G	C	-0.010	0.002	7.90E-10	38	-0.038	0.038	0.316
rs17400325	HAb1c	C	T	0.026	0.004	8.20E-12	47	-0.039	0.122	0.747
rs1776877	HAb1c	A	G	-0.022	0.002	4.60E-29	125	-0.097	0.048	0.042
rs1786812	HAb1c	C	G	0.011	0.002	1.30E-10	41	-0.026	0.038	0.481
rs17884312	HAb1c	T	C	0.012	0.002	5.10E-10	39	-0.004	0.046	0.930
rs183587141	HAb1c	T	C	-0.042	0.006	2.70E-12	49	-0.011	0.184	0.952
rs1867264	HAb1c	T	A	-0.010	0.002	2.70E-10	40	0.017	0.036	0.634
rs1892525	HAb1c	C	G	-0.009	0.002	6.20E-09	34	0.038	0.040	0.340
rs1909122	HAb1c	T	C	-0.014	0.002	6.60E-19	79	0.075	0.035	0.032
rs2079147	HAb1c	G	A	-0.010	0.002	3.30E-11	44	-0.010	0.034	0.772
rs2157323	HAb1c	C	T	0.009	0.002	2.10E-08	31	0.080	0.038	0.034
rs2167734	HAb1c	A	G	0.010	0.002	8.90E-10	38	-0.008	0.040	0.847
rs2219320	HAb1c	C	T	-0.014	0.002	1.30E-15	64	0.027	0.042	0.521
rs2236610	HAb1c	G	C	-0.011	0.002	1.50E-08	32	-0.004	0.045	0.923
rs225646	HAb1c	A	G	0.013	0.002	5.00E-14	57	-0.026	0.043	0.538
rs2294214	HAb1c	C	A	-0.014	0.002	1.40E-18	77	-0.031	0.039	0.422
rs2364232	HAb1c	C	A	0.016	0.002	1.40E-21	91	0.048	0.038	0.203
rs2412771	HAb1c	C	T	0.011	0.002	4.50E-13	52	0.009	0.034	0.781
rs2428551	HAb1c	A	T	0.011	0.002	6.00E-12	47	-0.023	0.039	0.546
rs2526625	HAb1c	C	A	-0.015	0.002	4.60E-18	75	-0.085	0.040	0.033
rs2544535	HAb1c	C	T	0.012	0.001	4.00E-16	66	-0.017	0.034	0.613
rs2579762	HAb1c	C	A	-0.014	0.001	9.50E-20	83	-0.041	0.034	0.221
rs2647242	HAb1c	G	A	-0.011	0.002	1.40E-09	37	-0.033	0.044	0.447
rs2648717	HAb1c	C	G	0.014	0.002	1.10E-12	51	-0.133	0.048	0.006
rs2678408	HAb1c	C	T	-0.012	0.001	1.80E-15	63	-0.004	0.034	0.897
rs2812208	HAb1c	C	G	0.068	0.005	1.20E-38	169	0.074	0.115	0.522

rs28469297	HAb1c	C	A	0.013	0.002	2.20E-08	31	0.022	0.050	0.657
rs2854152	HAb1c	G	A	0.017	0.002	1.30E-26	114	-0.011	0.035	0.755
rs28708703	HAb1c	G	C	0.013	0.002	7.10E-10	38	-0.035	0.050	0.481
rs2881019	HAb1c	C	T	-0.012	0.002	4.50E-14	57	-0.095	0.035	0.006
rs2885697	HAb1c	T	G	-0.015	0.002	2.20E-20	86	0.011	0.035	0.766
rs2974389	HAb1c	G	A	-0.011	0.002	2.70E-14	58	-0.012	0.034	0.720
rs335429	HAb1c	C	T	0.008	0.001	3.40E-08	30	-0.036	0.034	0.283
rs34587839	HAb1c	A	G	-0.016	0.002	5.00E-14	57	0.029	0.046	0.526
rs35506	HAb1c	A	T	0.010	0.002	4.10E-09	35	-0.028	0.039	0.478
rs35945722	HAb1c	A	G	0.016	0.002	1.80E-16	68	0.032	0.045	0.482
rs36226649	HAb1c	C	T	0.024	0.003	1.80E-15	63	0.040	0.077	0.601
rs3791679	HAb1c	G	A	-0.026	0.002	4.10E-47	208	0.004	0.040	0.926
rs3858603	HAb1c	A	G	-0.008	0.001	1.40E-08	32	-0.018	0.034	0.582
rs41311445	HAb1c	C	A	-0.017	0.003	5.20E-11	43	-0.008	0.049	0.871
rs425102	HAb1c	G	T	-0.012	0.002	4.60E-12	48	-0.024	0.043	0.581
rs4284505	HAb1c	G	A	-0.010	0.002	1.70E-10	41	-0.089	0.033	0.008
rs4320932	HAb1c	C	T	-0.013	0.002	3.10E-12	49	0.015	0.044	0.733
rs4584512	HAb1c	C	G	-0.009	0.002	3.70E-08	30	0.013	0.037	0.718
rs4813418	HAb1c	G	A	-0.009	0.002	5.60E-09	34	0.051	0.037	0.169
rs4836528	HAb1c	C	T	-0.013	0.002	1.10E-17	73	0.026	0.034	0.443
rs4894559	HAb1c	G	A	-0.009	0.002	2.10E-08	31	0.021	0.037	0.566
rs4909034	HAb1c	G	C	-0.010	0.002	8.90E-11	42	0.083	0.035	0.018
rs4909296	HAb1c	C	T	-0.010	0.001	4.10E-11	44	0.003	0.034	0.928
rs4988321	HAb1c	A	G	-0.026	0.003	3.10E-14	58	-0.049	0.137	0.719
rs513953	HAb1c	G	A	0.015	0.002	3.60E-18	76	0.014	0.039	0.721
rs520461	HAb1c	G	A	-0.012	0.002	4.60E-11	43	-0.083	0.039	0.033
rs55750792	HAb1c	A	G	0.011	0.001	4.90E-13	52	-0.008	0.034	0.814
rs55914134	HAb1c	G	A	-0.009	0.002	1.00E-08	33	0.032	0.036	0.366
rs56308303	HAb1c	T	C	0.009	0.002	3.20E-10	40	-0.022	0.034	0.509
rs56332326	HAb1c	C	T	-0.014	0.002	4.30E-10	39	0.016	0.042	0.697
rs56795193	HAb1c	C	T	0.010	0.002	2.80E-08	31	-0.014	0.040	0.727
rs59748147	HAb1c	C	T	0.020	0.003	7.10E-14	56	0.084	0.050	0.092
rs5992136	HAb1c	C	G	0.012	0.002	4.50E-13	52	-0.006	0.041	0.890
rs61465425	HAb1c	T	C	0.011	0.002	1.10E-08	33	-0.049	0.050	0.331
rs61846088	HAb1c	T	C	0.010	0.002	3.60E-08	30	0.046	0.042	0.272
rs61966248	HAb1c	C	T	-0.011	0.002	8.60E-09	33	0.020	0.048	0.677
rs62088172	HAb1c	T	C	0.009	0.002	3.10E-09	35	-0.012	0.034	0.719
rs62126408	HAb1c	C	T	0.017	0.002	2.70E-16	67	0.084	0.056	0.135
rs62275653	HAb1c	T	C	-0.024	0.003	2.70E-12	49	0.102	0.061	0.093
rs62437411	HAb1c	A	G	0.011	0.002	3.40E-12	48	0.019	0.035	0.580
rs62621197	HAb1c	T	C	-0.050	0.004	6.70E-34	147	-0.033	0.119	0.783

rs644570	HAb1c	A	G	0.009	0.002	2.00E-09	36	0.017	0.035	0.638
rs663378	HAb1c	A	G	-0.012	0.002	1.90E-09	36	-0.013	0.046	0.782
rs66678205	HAb1c	AT	A	-0.009	0.002	3.40E-08	30	0.000	0.034	0.992
rs6671370	HAb1c	A	G	0.016	0.002	8.90E-23	96	-0.004	0.036	0.920
rs6729308	HAb1c	A	G	-0.013	0.002	3.50E-18	76	0.008	0.034	0.815
rs67326273	HAb1c	G	A	0.011	0.002	4.30E-09	34	0.001	0.053	0.977
rs6762578	HAb1c	A	G	0.014	0.002	2.80E-14	58	-0.010	0.045	0.821
rs6823809	HAb1c	C	T	-0.023	0.002	1.10E-45	201	-0.022	0.038	0.560
rs6859171	HAb1c	T	C	0.012	0.002	8.40E-11	42	-0.002	0.040	0.963
rs6869268	HAb1c	C	T	-0.009	0.002	6.10E-09	34	0.003	0.036	0.938
rs7029491	HAb1c	G	C	0.011	0.002	1.10E-11	46	-0.021	0.037	0.581
rs7068966	HAb1c	T	C	0.017	0.001	3.70E-31	135	0.023	0.034	0.500
rs7075773	HAb1c	T	C	-0.012	0.002	5.70E-15	61	-0.002	0.034	0.944
rs7086293	HAb1c	C	T	-0.008	0.001	3.40E-08	30	0.006	0.034	0.869
rs71427097	HAb1c	T	C	-0.025	0.004	4.50E-08	30	0.020	0.098	0.835
rs723588	HAb1c	C	T	0.016	0.002	1.00E-14	60	0.010	0.045	0.833
rs72643433	HAb1c	A	G	-0.013	0.002	3.50E-13	53	0.010	0.042	0.808
rs72666766	HAb1c	G	A	-0.011	0.002	4.10E-08	30	0.119	0.057	0.037
rs72755233	HAb1c	A	G	-0.024	0.002	5.80E-25	106	0.050	0.053	0.349
rs72907878	HAb1c	A	G	-0.020	0.002	7.50E-21	88	-0.071	0.051	0.169
rs73048979	HAb1c	T	C	0.028	0.005	1.40E-09	37	-0.051	0.208	0.808
rs73175572	HAb1c	G	A	0.020	0.002	1.30E-17	73	-0.091	0.052	0.081
rs7326822	HAb1c	G	A	0.009	0.002	1.40E-08	32	0.050	0.034	0.139
rs735830	HAb1c	A	G	-0.013	0.002	1.10E-12	51	0.013	0.044	0.769
rs7359156	HAb1c	G	A	-0.012	0.002	6.10E-11	43	0.016	0.040	0.682
rs741593	HAb1c	G	T	-0.010	0.002	1.40E-09	37	0.049	0.036	0.172
rs7506068	HAb1c	G	C	0.009	0.002	3.00E-09	35	0.061	0.035	0.078
rs75216331	HAb1c	A	G	-0.011	0.002	2.90E-10	40	-0.036	0.045	0.430
rs75301758	HAb1c	T	C	-0.011	0.002	5.10E-09	34	-0.038	0.039	0.330
rs7546750	HAb1c	A	G	0.010	0.002	1.50E-10	41	-0.021	0.035	0.548
rs7558918	HAb1c	G	T	0.014	0.001	4.10E-20	84	-0.034	0.034	0.311
rs7582299	HAb1c	C	T	-0.010	0.002	4.00E-08	30	-0.020	0.037	0.583
rs7663740	HAb1c	T	G	0.024	0.001	2.40E-60	269	0.052	0.035	0.131
rs769675	HAb1c	C	T	0.009	0.002	2.20E-08	31	0.010	0.036	0.783
rs76993203	HAb1c	A	G	-0.014	0.001	5.60E-20	84	0.030	0.034	0.385
rs77156651	HAb1c	A	G	-0.020	0.003	8.20E-10	38	-0.088	0.094	0.351
rs7753558	HAb1c	A	C	0.010	0.002	9.50E-10	37	0.008	0.036	0.826
rs78304176	HAb1c	T	G	-0.020	0.003	3.10E-11	44	-0.018	0.084	0.826
rs7854962	HAb1c	G	C	-0.015	0.002	1.20E-15	64	0.027	0.043	0.527
rs78808004	HAb1c	G	A	0.015	0.003	2.00E-09	36	0.038	0.074	0.613
rs79409628	HAb1c	T	G	0.020	0.003	2.70E-14	58	-0.138	0.063	0.030

rs7954392	HAb1c	A	C	0.014	0.003	3.60E-08	30	0.024	0.069	0.725
rs8037367	HAb1c	C	T	0.011	0.002	1.40E-10	41	0.002	0.044	0.966
rs8069700	HAb1c	A	G	-0.010	0.002	3.20E-08	31	-0.051	0.039	0.192
rs820077	HAb1c	A	G	0.021	0.002	2.00E-29	127	-0.082	0.062	0.186
rs841216	HAb1c	G	A	-0.009	0.002	1.20E-08	33	-0.019	0.034	0.577
rs879394	HAb1c	T	G	-0.016	0.002	9.30E-19	78	-0.016	0.038	0.674
rs910969	HAb1c	G	A	0.009	0.002	1.40E-08	32	-0.026	0.036	0.481
rs9271420	HAb1c	G	A	-0.014	0.002	4.70E-19	80	0.045	0.034	0.183
rs9295	HAb1c	A	G	0.011	0.002	4.60E-11	43	-0.093	0.037	0.011
rs9310389	HAb1c	T	C	0.009	0.002	7.00E-10	38	-0.036	0.034	0.289
rs9321170	HAb1c	A	G	0.011	0.002	3.30E-11	44	-0.019	0.035	0.581
rs9672558	HAb1c	C	T	-0.014	0.002	4.00E-11	44	-0.031	0.065	0.629
rs9701805	HAb1c	C	G	0.019	0.003	1.80E-13	54	0.007	0.072	0.918
rs980896	HAb1c	A	G	0.010	0.001	1.40E-11	46	0.013	0.034	0.698
rs9912553	HAb1c	G	C	0.016	0.002	2.90E-22	94	0.004	0.039	0.918
rs9970286	HAb1c	A	G	0.009	0.002	8.80E-09	33	0.041	0.035	0.244

HAb1c: glycosylated hemoglobin . EA: effect allele. OA: other allele. EAF: EA frequency. GX: beta-exposure. GX(SE): standard error of GX. GY: beta-outcome. GY(SE): standard error of GY.

Table S6.Published associations of FI on PTB

SNP	Exposure	EA	OA	GX	GX(SE)	pval	F	GY	GY(SE)	pval
rs10050393	FI	C	T	-0.009	0.002	4.84E-08	22	0.021	0.034	0.533
rs11708067	FI	G	A	0.014	0.002	1.30E-09	34	-0.039	0.045	0.382
rs11727676	FI	C	T	0.020	0.004	2.90E-08	27	-0.102	0.060	0.090
rs118164457	FI	C	T	0.035	0.006	3.86E-10	37	0.004	0.148	0.981
rs1206760	FI	A	G	-0.011	0.002	8.82E-10	35	0.033	0.034	0.325
rs12454712	FI	C	T	-0.014	0.003	1.78E-09	32	0.019	0.034	0.564
rs13258890	FI	C	T	-0.013	0.003	2.77E-08	26	-0.044	0.041	0.287
rs17036126	FI	T	C	0.021	0.003	1.28E-10	49	0.011	0.057	0.850
rs17331151	FI	T	C	-0.016	0.003	1.52E-08	27	-0.060	0.059	0.306
rs2108349	FI	A	G	-0.012	0.002	1.13E-08	33	0.023	0.034	0.495
rs2780215	FI	G	A	-0.039	0.006	1.06E-09	39	0.146	0.100	0.144
rs2845885	FI	T	C	-0.020	0.004	1.18E-08	27	-0.016	0.063	0.797
rs2943646	FI	G	A	0.025	0.002	8.47E-39	173	0.034	0.035	0.328
rs4865796	FI	A	G	0.017	0.002	7.33E-17	68	-0.058	0.036	0.103
rs5017305	FI	T	A	-0.014	0.003	1.07E-08	28	0.006	0.045	0.903
rs6487237	FI	A	C	0.015	0.003	4.68E-09	35	-0.034	0.043	0.430
rs6855363	FI	C	T	-0.013	0.002	4.04E-08	39	-0.038	0.039	0.334
rs7012814	FI	A	G	-0.022	0.002	8.34E-30	133	0.017	0.034	0.610
rs7133378	FI	A	G	-0.013	0.002	6.00E-11	40	-0.055	0.037	0.144
rs73013411	FI	A	C	-0.018	0.003	2.08E-08	32	-0.023	0.061	0.708
rs731839	FI	A	G	-0.012	0.002	3.86E-11	41	-0.007	0.035	0.853
rs7903146	FI	T	C	-0.012	0.002	1.24E-09	31	-0.020	0.042	0.633
rs860598	FI	A	G	0.018	0.003	6.88E-12	50	0.024	0.041	0.548
rs972283	FI	G	A	0.011	0.002	1.09E-08	31	-0.044	0.034	0.193

FI: fasting insulin. EA: effect allele. OA: other allele. EAF: EA frequency. GX: beta-exposure. GX(SE): standard error of GX. GY: beta-outcome. GY(SE): standard error of GY.

Table S7.Published associations of LDL-C on PTB

SNP	Exposure	EA	OA	GX	GX(SE)	pval	F	GY	GY(SE)	pval
rs10490626	LDL-C	A	G	-0.051	0.007	1.70E-12	54	0.124	0.065	0.056
rs10832962	LDL-C	T	C	0.032	0.004	6.62E-14	64	0.043	0.035	0.217
rs10893499	LDL-C	A	G	0.052	0.005	3.86E-21	97	0.056	0.043	0.188
rs10947332	LDL-C	A	G	0.050	0.006	6.97E-18	81	-0.182	0.044	0.000
rs112201728	LDL-C	T	C	0.068	0.010	8.51E-10	42	-0.100	0.072	0.163
rs11563251	LDL-C	T	C	0.035	0.006	4.50E-08	31	-0.006	0.057	0.909
rs11591147	LDL-C	T	G	-0.497	0.018	8.57E-143	762	-0.057	0.091	0.532
rs1169288	LDL-C	C	A	0.038	0.004	6.45E-21	88	-0.009	0.035	0.790
rs12066643	LDL-C	T	C	-0.039	0.006	1.06E-08	37	-0.022	0.050	0.662
rs1250229	LDL-C	C	T	0.024	0.004	3.13E-08	33	0.051	0.042	0.220
rs12721109	LDL-C	A	G	-0.446	0.018	2.99E-122	595	-0.137	0.123	0.264
rs12748152	LDL-C	T	C	0.050	0.007	3.21E-12	57	0.022	0.063	0.725
rs12916	LDL-C	C	T	0.073	0.004	7.79E-78	372	-0.031	0.034	0.355
rs13277801	LDL-C	T	C	-0.034	0.004	3.99E-17	79	-0.022	0.035	0.522
rs1367117	LDL-C	A	G	0.119	0.004	9.48E-183	879	-0.002	0.037	0.957
rs1564348	LDL-C	C	T	0.048	0.005	2.76E-21	93	-0.070	0.048	0.147
rs16831243	LDL-C	T	C	0.038	0.006	9.06E-12	47	0.051	0.047	0.278
rs16891156	LDL-C	C	A	0.097	0.017	8.23E-09	32	0.031	0.112	0.786
rs1801689	LDL-C	C	A	0.103	0.014	9.81E-12	55	-0.076	0.172	0.659
rs1883025	LDL-C	T	C	-0.030	0.004	6.14E-11	45	-0.076	0.042	0.072
rs2000999	LDL-C	A	G	0.065	0.005	4.22E-41	200	0.007	0.042	0.873
rs2030746	LDL-C	T	C	0.021	0.004	8.60E-09	32	0.053	0.035	0.130
rs2073547	LDL-C	G	A	0.049	0.005	1.92E-21	98	-0.017	0.036	0.631
rs2228603	LDL-C	T	C	-0.104	0.007	4.43E-44	209	0.077	0.067	0.252
rs2315065	LDL-C	A	C	0.110	0.016	5.23E-12	49	0.084	0.069	0.228
rs2328223	LDL-C	C	A	0.030	0.005	5.63E-09	36	-0.031	0.042	0.461
rs2390536	LDL-C	A	G	0.022	0.004	2.04E-08	34	0.033	0.035	0.341
rs2419604	LDL-C	G	A	-0.030	0.004	7.49E-14	57	-0.038	0.036	0.288
rs247616	LDL-C	T	C	-0.055	0.004	2.57E-37	178	0.038	0.037	0.308
rs2495495	LDL-C	C	T	-0.034	0.006	3.52E-08	34	0.033	0.047	0.488
rs2587534	LDL-C	A	G	0.039	0.004	8.06E-25	112	-0.013	0.034	0.699
rs2642438	LDL-C	G	A	0.035	0.004	7.32E-16	70	-0.035	0.038	0.350
rs267733	LDL-C	G	A	-0.033	0.005	5.29E-09	39	-0.046	0.048	0.338
rs2710642	LDL-C	A	G	0.024	0.004	6.09E-09	40	-0.045	0.035	0.194
rs2737252	LDL-C	A	G	-0.031	0.004	7.04E-14	59	0.028	0.036	0.449
rs2738459	LDL-C	C	A	-0.053	0.006	2.26E-19	84	-0.005	0.034	0.873
rs2965157	LDL-C	C	T	-0.189	0.011	7.29E-62	284	0.066	0.085	0.440
rs314253	LDL-C	C	T	-0.024	0.004	3.44E-10	41	-0.031	0.034	0.374
rs364585	LDL-C	G	A	0.025	0.004	4.28E-10	43	0.003	0.035	0.933

rs3780181	LDL-C	G	A	-0.045	0.007	1.76E-09	36	-0.049	0.074	0.511
rs4253776	LDL-C	G	A	0.031	0.006	3.35E-08	28	-0.044	0.061	0.475
rs4530754	LDL-C	A	G	0.028	0.004	3.58E-12	58	-0.014	0.034	0.680
rs4722551	LDL-C	C	T	0.039	0.005	3.95E-14	64	-0.013	0.041	0.761
rs4942486	LDL-C	C	T	-0.024	0.004	2.26E-11	43	0.024	0.034	0.472
rs4970712	LDL-C	C	A	0.034	0.004	2.46E-13	59	0.000	0.040	0.998
rs4970834	LDL-C	T	C	-0.150	0.005	1.00E-200	1023	0.025	0.042	0.561
rs5763662	LDL-C	T	C	0.077	0.012	1.19E-08	40	0.052	0.093	0.579
rs6016373	LDL-C	G	A	-0.035	0.004	7.95E-19	89	-0.040	0.035	0.260
rs6504872	LDL-C	T	C	0.027	0.004	3.48E-13	55	0.028	0.034	0.401
rs6511720	LDL-C	T	G	-0.221	0.006	1.00E-200	1311	-0.019	0.056	0.732
rs6544713	LDL-C	C	T	-0.081	0.004	4.84E-83	386	0.017	0.041	0.675
rs6693893	LDL-C	C	T	-0.077	0.013	2.88E-08	34	-0.060	0.077	0.432
rs6709904	LDL-C	G	A	-0.055	0.009	4.58E-10	42	0.006	0.056	0.917
rs6818397	LDL-C	G	T	-0.022	0.004	1.68E-08	31	-0.049	0.038	0.193
rs6882076	LDL-C	C	T	0.046	0.004	3.31E-31	144	-0.012	0.036	0.737
rs6909746	LDL-C	T	C	-0.026	0.004	7.86E-11	51	0.045	0.034	0.187
rs7254892	LDL-C	A	G	-0.485	0.012	1.00E-200	1663	-0.089	0.113	0.429
rs72902576	LDL-C	G	T	-0.093	0.013	9.58E-12	49	0.035	0.079	0.656
rs7551981	LDL-C	T	G	0.047	0.004	1.36E-33	154	-0.038	0.034	0.259
rs75687619	LDL-C	T	G	0.174	0.016	8.05E-24	116	0.090	0.112	0.420
rs7640978	LDL-C	T	C	-0.039	0.007	9.84E-09	32	0.024	0.061	0.692
rs7832643	LDL-C	T	G	0.034	0.004	2.67E-17	80	-0.024	0.034	0.478
rs8017377	LDL-C	A	G	0.030	0.004	2.52E-15	64	-0.049	0.035	0.157

LDL-C, low-density lipoprotein cholesterol. EA: effect allele. OA: other allele. EAF: EA frequency. GX: beta-exposure. GX(SE): standard error of GX. GY: beta-outcome. GY(SE): standard error of GY.

Table S8.Published associations of HDL-C and TB

SNP	Exposure	EA	OA	GX	GX(SE)	pval	F	GY	GY(SE)	pval
rs10019888	HDL-C	G	A	-0.027	0.005	4.90E-08	34	0.071	0.049	0.153
rs10087900	HDL-C	A	G	-0.023	0.004	2.17E-09	41	0.007	0.035	0.833
rs103294	HDL-C	T	C	0.052	0.004	3.99E-30	141	0.035	0.037	0.337
rs11045163	HDL-C	G	A	0.022	0.004	3.20E-09	38	0.028	0.035	0.411
rs11765979	HDL-C	C	A	0.041	0.005	3.11E-17	74	0.049	0.034	0.146
rs11789603	HDL-C	T	C	0.060	0.006	3.69E-21	100	-0.048	0.064	0.456
rs12133576	HDL-C	G	A	-0.024	0.004	6.15E-11	48	-0.066	0.035	0.062
rs12145743	HDL-C	G	T	0.020	0.004	1.80E-08	32	0.015	0.035	0.672
rs12412743	HDL-C	T	C	-0.029	0.005	1.31E-09	42	-0.028	0.046	0.542
rs12740374	HDL-C	T	G	0.034	0.004	1.69E-15	70	0.028	0.041	0.501
rs12748152	HDL-C	T	C	-0.051	0.006	9.74E-16	67	0.022	0.063	0.725
rs13076253	HDL-C	C	A	-0.028	0.005	4.96E-09	35	-0.036	0.043	0.410
rs13099479	HDL-C	A	G	0.036	0.006	1.82E-08	34	-0.067	0.062	0.286
rs13702	HDL-C	C	T	0.106	0.004	1.28E-160	775	0.034	0.038	0.370
rs1515110	HDL-C	T	G	-0.032	0.004	8.04E-18	85	0.028	0.035	0.417
rs1689797	HDL-C	A	C	-0.036	0.004	2.85E-21	99	-0.011	0.037	0.771
rs16965220	HDL-C	A	C	0.022	0.004	7.91E-09	35	0.009	0.037	0.814
rs17173637	HDL-C	C	T	-0.036	0.006	1.90E-08	41	-0.038	0.048	0.426
rs1866956	HDL-C	T	C	0.022	0.004	7.96E-10	34	-0.023	0.035	0.510
rs1883025	HDL-C	T	C	-0.070	0.004	1.50E-65	290	-0.076	0.042	0.072
rs205262	HDL-C	G	A	-0.028	0.004	3.88E-13	53	0.049	0.038	0.191
rs2066714	HDL-C	C	T	0.045	0.007	7.26E-10	41	0.036	0.058	0.535
rs2075650	HDL-C	G	A	-0.055	0.005	9.72E-26	118	0.010	0.045	0.817
rs2241210	HDL-C	G	A	0.033	0.004	2.49E-20	90	0.017	0.034	0.607
rs2241770	HDL-C	C	T	-0.099	0.006	6.78E-60	301	-0.101	0.054	0.064
rs2250802	HDL-C	A	G	-0.034	0.004	2.02E-17	80	-0.042	0.036	0.247
rs2278236	HDL-C	A	G	0.033	0.004	3.18E-18	89	0.009	0.034	0.801
rs2290547	HDL-C	A	G	-0.030	0.005	3.69E-09	42	0.002	0.040	0.960
rs2454722	HDL-C	G	A	0.035	0.004	3.31E-14	64	0.009	0.046	0.840
rs2602836	HDL-C	G	A	-0.019	0.003	4.96E-08	32	0.012	0.034	0.717
rs2606736	HDL-C	T	C	-0.025	0.004	4.80E-08	33	0.013	0.034	0.707
rs2642438	HDL-C	G	A	0.030	0.004	7.78E-14	60	-0.035	0.038	0.350
rs2925979	HDL-C	C	T	0.035	0.004	1.32E-19	90	0.056	0.036	0.119
rs3741414	HDL-C	T	C	0.030	0.004	6.10E-14	55	0.022	0.040	0.588
rs3847502	HDL-C	A	C	0.048	0.004	3.31E-38	178	0.036	0.034	0.297
rs4142995	HDL-C	T	G	-0.026	0.004	9.36E-12	51	-0.004	0.037	0.920
rs4148005	HDL-C	G	T	-0.028	0.004	5.74E-14	62	-0.005	0.037	0.888
rs424346	HDL-C	T	C	0.068	0.011	4.84E-08	36	-0.018	0.090	0.842
rs4379922	HDL-C	C	T	0.025	0.004	9.56E-12	47	0.037	0.035	0.285

rs4650994	HDL-C	A	G	-0.021	0.003	6.70E-09	38	-0.001	0.034	0.988
rs4846914	HDL-C	A	G	0.048	0.003	3.51E-41	198	0.000	0.034	0.999
rs4917014	HDL-C	G	T	0.022	0.004	1.03E-08	38	0.011	0.036	0.769
rs4939883	HDL-C	C	T	0.080	0.005	1.80E-66	315	-0.003	0.044	0.951
rs4983559	HDL-C	A	G	-0.020	0.004	9.57E-09	30	0.006	0.035	0.866
rs6031587	HDL-C	T	C	-0.049	0.007	1.92E-09	43	0.036	0.065	0.578
rs633695	HDL-C	G	A	0.089	0.005	7.82E-58	269	0.029	0.037	0.429
rs6450176	HDL-C	A	G	-0.025	0.004	6.87E-10	42	-0.001	0.039	0.971
rs676210	HDL-C	A	G	0.066	0.004	2.34E-54	272	-0.031	0.038	0.415
rs6805251	HDL-C	C	T	-0.020	0.004	1.33E-08	33	-0.044	0.034	0.200
rs686030	HDL-C	A	C	0.055	0.005	4.29E-27	126	-0.042	0.052	0.423
rs687339	HDL-C	T	C	-0.032	0.004	7.11E-13	57	-0.039	0.047	0.404
rs702485	HDL-C	G	A	0.024	0.003	6.45E-12	51	-0.010	0.034	0.769
rs7306660	HDL-C	A	G	-0.035	0.004	3.34E-19	92	-0.006	0.036	0.873
rs731839	HDL-C	A	G	0.022	0.004	3.44E-09	35	-0.007	0.035	0.853
rs737337	HDL-C	C	T	-0.057	0.006	4.56E-17	86	-0.037	0.059	0.532
rs838876	HDL-C	G	A	-0.049	0.004	7.32E-33	160	0.000	0.034	0.989
rs9457931	HDL-C	G	A	-0.055	0.007	7.30E-13	57	0.001	0.053	0.985
rs9989419	HDL-C	G	A	0.147	0.004	1.00E-200	1674	0.019	0.035	0.589

HDL-C, high-density lipoprotein cholesterol. EA: effect allele. OA: other allele. EAF: EA frequency. GX: beta-exposure. GX(SE): standard error of GX. GY: beta-outcome. GY(SE): standard error of GY.

Table S9.Published associations of TG on PTB

SNP	Exposure	EA	OA	GX	GX(SE)	pval	F	GY	GY(SE)	pval
rs10401969	TG	C	T	-0.121	0.007	9.70E-70	347	0.080	0.069	0.250
rs10440120	TG	A	C	-0.031	0.004	5.34E-11	48	0.011	0.042	0.784
rs10501321	TG	C	T	-0.022	0.004	1.41E-08	38	0.035	0.034	0.303
rs11057408	TG	T	G	-0.026	0.004	2.05E-12	54	-0.059	0.037	0.106
rs11613352	TG	T	C	-0.028	0.004	9.40E-14	52	0.023	0.040	0.558
rs12280753	TG	T	C	0.193	0.006	1.22E-179	910	0.079	0.065	0.227
rs12676857	TG	C	T	0.033	0.005	7.29E-12	52	0.050	0.043	0.250
rs12748152	TG	T	C	0.037	0.006	1.10E-09	40	0.022	0.063	0.725
rs174535	TG	C	T	0.047	0.003	1.73E-41	191	0.015	0.034	0.663
rs2043085	TG	C	T	-0.033	0.003	7.81E-20	92	0.036	0.034	0.291
rs2068888	TG	A	G	-0.024	0.003	1.68E-11	50	0.047	0.034	0.163
rs2250802	TG	A	G	0.023	0.004	1.21E-10	39	-0.042	0.036	0.247
rs247616	TG	T	C	-0.039	0.004	1.12E-25	113	0.038	0.037	0.308
rs2665357	TG	C	A	0.021	0.003	8.33E-10	41	-0.029	0.034	0.392
rs287621	TG	C	T	-0.022	0.004	7.67E-09	36	-0.012	0.037	0.736
rs2972146	TG	T	G	0.028	0.003	2.97E-15	68	0.034	0.035	0.329
rs3198697	TG	T	C	-0.020	0.003	2.21E-08	34	-0.039	0.034	0.256
rs3760627	TG	C	T	0.019	0.003	5.29E-09	31	0.001	0.034	0.971
rs3761445	TG	A	G	0.023	0.003	8.06E-12	47	0.016	0.034	0.637
rs38855	TG	G	A	-0.019	0.003	2.11E-08	32	-0.040	0.034	0.236
rs4587594	TG	A	G	-0.069	0.004	3.50E-82	393	0.023	0.038	0.545
rs4719841	TG	G	A	0.023	0.003	8.86E-11	47	0.004	0.035	0.913
rs6029143	TG	T	C	-0.039	0.007	4.93E-08	30	0.022	0.092	0.810
rs645040	TG	T	G	0.029	0.004	1.83E-12	54	-0.039	0.047	0.408
rs676210	TG	A	G	-0.073	0.004	3.28E-71	353	-0.031	0.038	0.415
rs6831256	TG	G	A	0.026	0.004	1.60E-12	54	0.024	0.035	0.489
rs6882076	TG	C	T	0.029	0.004	1.51E-15	67	-0.012	0.036	0.737
rs6995541	TG	G	A	0.027	0.004	1.34E-12	51	-0.004	0.038	0.910
rs7248104	TG	A	G	-0.022	0.003	5.04E-10	43	0.030	0.034	0.374
rs731839	TG	A	G	-0.022	0.004	2.65E-09	39	-0.007	0.035	0.853
rs8077889	TG	C	A	0.025	0.004	9.88E-09	36	0.072	0.041	0.079
rs948690	TG	C	T	-0.031	0.005	6.57E-09	35	-0.004	0.040	0.929

TG, triglycerides. EA: effect allele. OA: other allele. EAF: EA frequency. GX: beta-exposure. GX(SE): standard error of GX. GY: beta-outcome. GY(SE): standard error of GY.

Table S10.Published associations of TC on PTB

SNP	Exxposure	EA	OA	GX	GX(SE)	pval	F	GY	GY(SE)	pval
rs10088180	TC	G	A	-0.023	0.004	6.02E-10	32	0.040	0.036	0.258
rs10773003	TC	A	G	0.037	0.006	4.08E-09	40	-0.022	0.053	0.682
rs10832962	TC	T	C	0.032	0.004	1.54E-14	65	0.043	0.035	0.217
rs10904908	TC	G	A	0.025	0.004	2.60E-11	48	-0.015	0.034	0.659
rs11153594	TC	T	C	-0.029	0.004	1.27E-14	65	0.045	0.034	0.187
rs112201728	TC	T	C	0.058	0.010	1.20E-08	34	-0.100	0.072	0.163
rs11220462	TC	A	G	0.047	0.006	5.49E-15	67	0.058	0.043	0.179
rs11563251	TC	T	C	0.037	0.006	1.27E-09	39	-0.006	0.057	0.909
rs11591147	TC	T	G	-0.334	0.017	8.83E-86	373	-0.057	0.091	0.532
rs11694172	TC	G	A	0.028	0.004	1.95E-09	46	0.025	0.045	0.584
rs11753995	TC	A	G	0.049	0.005	1.84E-23	104	-0.076	0.048	0.116
rs11789603	TC	T	C	0.043	0.006	1.44E-11	47	-0.048	0.064	0.456
rs12412743	TC	T	C	-0.030	0.005	6.98E-10	40	-0.028	0.046	0.542
rs12670798	TC	C	T	0.036	0.004	9.48E-17	79	0.030	0.039	0.444
rs12916	TC	C	T	0.068	0.004	4.55E-74	361	-0.031	0.034	0.355
rs13315871	TC	A	G	-0.036	0.006	3.48E-08	34	-0.120	0.060	0.045
rs138777	TC	G	A	-0.021	0.004	4.74E-08	33	0.008	0.034	0.822
rs17526895	TC	G	A	-0.042	0.007	5.78E-09	39	0.124	0.065	0.055
rs1883025	TC	T	C	-0.067	0.004	5.75E-53	255	-0.076	0.042	0.072
rs1997243	TC	G	A	0.033	0.005	2.72E-10	44	-0.077	0.051	0.129
rs2000999	TC	A	G	0.062	0.004	6.80E-41	197	0.007	0.042	0.873
rs2030746	TC	T	C	0.020	0.004	3.60E-08	29	0.053	0.035	0.130
rs2066714	TC	C	T	0.044	0.008	1.14E-08	34	0.036	0.058	0.535
rs2073547	TC	G	A	0.046	0.005	3.83E-21	94	-0.017	0.036	0.631
rs2156552	TC	T	A	0.057	0.005	1.25E-31	147	-0.003	0.044	0.940
rs2228603	TC	T	C	-0.122	0.007	1.05E-62	311	0.077	0.067	0.252
rs2255141	TC	G	A	-0.031	0.004	6.51E-16	65	-0.040	0.036	0.265
rs2277862	TC	T	C	-0.035	0.005	5.26E-11	45	0.079	0.059	0.177
rs2315065	TC	A	C	0.110	0.016	1.10E-11	49	0.084	0.069	0.228
rs247616	TC	T	C	0.050	0.004	4.47E-32	156	0.038	0.037	0.308
rs2642438	TC	G	A	0.037	0.004	1.28E-18	86	-0.035	0.038	0.350
rs2737252	TC	A	G	-0.033	0.004	1.63E-16	72	0.028	0.036	0.449
rs2738459	TC	C	A	-0.039	0.006	2.11E-11	46	-0.005	0.034	0.873
rs281393	TC	T	C	-0.032	0.006	4.26E-08	34	-0.046	0.034	0.184
rs2814982	TC	T	C	-0.044	0.006	3.68E-15	60	0.105	0.046	0.023
rs314253	TC	C	T	-0.023	0.004	2.81E-10	40	-0.031	0.034	0.374
rs3780181	TC	G	A	-0.044	0.007	6.67E-10	39	-0.049	0.074	0.511
rs386003	TC	T	G	0.034	0.006	4.52E-08	35	0.030	0.036	0.405
rs4253772	TC	T	C	0.032	0.006	9.85E-09	31	-0.042	0.062	0.503

rs4530754	TC	A	G	0.023	0.004	1.68E-09	42	-0.014	0.034	0.680
rs4883201	TC	G	A	-0.035	0.006	1.74E-09	39	0.045	0.052	0.385
rs4988235	TC	A	G	-0.031	0.004	3.97E-14	59	-0.022	0.035	0.523
rs515135	TC	C	T	0.124	0.005	6.38E-151	724	-0.019	0.043	0.661
rs558971	TC	G	A	0.040	0.004	7.02E-28	122	-0.011	0.034	0.750
rs581080	TC	C	G	0.038	0.005	1.02E-13	64	-0.059	0.048	0.218
rs6016373	TC	G	A	-0.032	0.004	1.00E-17	79	-0.040	0.035	0.260
rs633695	TC	G	A	0.043	0.006	1.05E-14	56	0.029	0.037	0.429
rs646776	TC	T	C	0.127	0.004	4.78E-187	917	-0.027	0.041	0.513
rs6504872	TC	T	C	0.025	0.004	6.99E-12	51	0.028	0.034	0.401
rs6511720	TC	T	G	-0.185	0.006	1.00E-200	984	-0.019	0.056	0.732
rs6544713	TC	C	T	-0.077	0.004	1.69E-81	373	0.017	0.041	0.675
rs6573778	TC	C	T	-0.026	0.004	2.96E-11	45	0.023	0.034	0.506
rs6603981	TC	T	C	0.035	0.004	7.85E-15	67	0.001	0.040	0.989
rs6709904	TC	G	A	-0.055	0.008	8.39E-10	43	0.006	0.056	0.917
rs6818397	TC	G	T	-0.025	0.004	9.51E-11	42	-0.049	0.038	0.193
rs6882076	TC	C	T	0.051	0.004	5.35E-41	189	-0.012	0.036	0.737
rs7412	TC	T	C	-0.374	0.010	1.00E-200	1514	-0.149	0.074	0.044
rs7551981	TC	T	G	0.036	0.004	7.50E-22	94	-0.038	0.034	0.259
rs75687619	TC	T	G	0.159	0.015	3.61E-22	108	0.090	0.112	0.420
rs7640978	TC	T	C	-0.038	0.007	1.66E-08	32	0.024	0.061	0.692
rs7832643	TC	T	G	0.029	0.004	3.12E-13	61	-0.024	0.034	0.478
rs8103315	TC	A	C	0.042	0.006	5.94E-15	59	-0.034	0.043	0.433
rs9306897	TC	C	T	-0.049	0.004	7.51E-37	174	-0.014	0.035	0.686

TC, total cholesterol. EA: effect allele. OA: other allele. EAF: EA frequency. GX: beta-exposure. GX(SE): standard error of GX. GY: beta-outcome. GY(SE): standard error of GY.

Table S11.Published associations of BMI on PTB

SNP	Exposure	EA	OA	GX	GX(SE)	pval	F	GY	GY(SE)	pval
rs1000940	BMI	G	A	0.019	0.003	1.28E-08	32	-0.042	0.034	0.220
rs10132280	BMI	A	C	-0.023	0.003	1.14E-11	46	0.026	0.035	0.462
rs1016287	BMI	C	T	-0.023	0.003	2.25E-11	45	-0.034	0.039	0.381
rs10182181	BMI	G	A	0.031	0.003	8.78E-24	98	-0.013	0.034	0.700
rs10733682	BMI	G	A	-0.017	0.003	1.83E-08	32	-0.002	0.034	0.964
rs10938397	BMI	G	A	0.040	0.003	3.20E-38	168	0.020	0.034	0.550
rs10968576	BMI	G	A	0.025	0.003	6.61E-14	57	0.000	0.035	0.994
rs11057405	BMI	A	G	-0.031	0.006	2.02E-08	31	0.005	0.058	0.927
rs11165643	BMI	T	C	0.022	0.003	2.07E-12	49	-0.041	0.034	0.237
rs1167827	BMI	G	A	0.020	0.003	6.33E-10	37	-0.029	0.034	0.393
rs11727676	BMI	C	T	-0.036	0.006	2.55E-08	31	-0.102	0.060	0.090
rs12286929	BMI	G	A	0.022	0.003	1.31E-12	49	-0.039	0.034	0.253
rs12429545	BMI	A	G	0.033	0.005	1.09E-12	51	-0.007	0.049	0.887
rs12940622	BMI	A	G	-0.018	0.003	2.49E-09	34	0.031	0.035	0.381
rs12986742	BMI	C	T	0.021	0.004	1.01E-08	33	-0.021	0.034	0.537
rs13191362	BMI	G	A	-0.028	0.005	7.34E-09	33	0.015	0.070	0.831
rs1516725	BMI	C	T	0.045	0.005	1.89E-22	96	-0.027	0.056	0.633
rs1528435	BMI	T	C	0.018	0.003	1.20E-08	33	-0.003	0.035	0.934
rs16851483	BMI	T	G	0.048	0.008	3.55E-10	39	0.052	0.078	0.503
rs16951275	BMI	C	T	-0.031	0.004	1.91E-17	71	0.065	0.046	0.153
rs17001654	BMI	G	C	0.031	0.005	7.76E-09	33	-0.049	0.053	0.360
rs17024393	BMI	C	T	0.066	0.009	7.03E-14	56	-0.168	0.074	0.023
rs17094222	BMI	C	T	0.025	0.004	5.94E-11	43	-0.035	0.039	0.377
rs17405819	BMI	C	T	-0.022	0.003	2.07E-11	46	0.022	0.037	0.552
rs17724992	BMI	G	A	-0.019	0.004	3.41E-08	31	-0.054	0.040	0.181
rs1928295	BMI	C	T	-0.019	0.003	7.91E-10	37	0.011	0.034	0.749
rs2033529	BMI	G	A	0.019	0.003	1.39E-08	33	0.038	0.036	0.292
rs2033732	BMI	C	T	0.019	0.004	4.89E-08	30	0.028	0.039	0.479
rs205262	BMI	G	A	0.022	0.004	1.75E-10	40	0.049	0.038	0.191
rs2112347	BMI	G	T	-0.026	0.003	6.19E-17	71	-0.040	0.034	0.237
rs2121279	BMI	T	C	0.025	0.004	2.31E-08	31	0.008	0.040	0.836
rs2176598	BMI	C	T	-0.020	0.004	2.97E-08	30	0.048	0.038	0.208
rs2207139	BMI	G	A	0.045	0.004	4.13E-29	125	-0.040	0.042	0.333
rs2245368	BMI	T	C	-0.032	0.006	3.19E-08	31	0.004	0.041	0.918
rs2287019	BMI	T	C	-0.036	0.004	4.58E-18	73	-0.072	0.040	0.072
rs2365389	BMI	T	C	-0.020	0.003	1.63E-10	42	0.003	0.034	0.930
rs2820292	BMI	C	A	0.020	0.003	1.83E-10	40	0.014	0.034	0.674
rs29941	BMI	G	A	0.018	0.003	2.41E-08	30	-0.019	0.034	0.586
rs3736485	BMI	G	A	-0.018	0.003	7.41E-09	32	0.034	0.034	0.318

rs3849570	BMI	A	C	0.019	0.003	2.60E-08	31	0.028	0.034	0.416
rs4256980	BMI	G	C	0.021	0.003	2.90E-11	45	-0.070	0.035	0.047
rs4740619	BMI	C	T	-0.018	0.003	4.56E-09	33	-0.060	0.034	0.077
rs543874	BMI	G	A	0.048	0.004	2.62E-35	153	-0.013	0.044	0.766
rs6477694	BMI	T	C	-0.017	0.003	2.67E-08	32	-0.060	0.034	0.084
rs657452	BMI	G	A	-0.023	0.003	5.48E-13	54	-0.011	0.034	0.755
rs6804842	BMI	G	A	0.019	0.003	2.48E-09	36	-0.031	0.034	0.352
rs7138803	BMI	A	G	0.032	0.003	8.15E-24	103	-0.021	0.035	0.546
rs7141420	BMI	T	C	0.024	0.003	1.23E-14	57	0.027	0.034	0.432
rs758747	BMI	T	C	0.023	0.004	7.47E-10	37	0.031	0.042	0.455
rs7599312	BMI	A	G	-0.022	0.003	1.17E-10	42	0.020	0.037	0.578
rs7899106	BMI	G	A	0.040	0.007	2.96E-08	31	-0.074	0.082	0.366
rs7903146	BMI	T	C	-0.023	0.003	1.11E-11	47	-0.020	0.042	0.633
rs879620	BMI	T	C	0.024	0.004	1.06E-09	37	-0.018	0.034	0.592
rs9400239	BMI	C	T	0.019	0.003	1.61E-08	32	0.014	0.035	0.681
rs9579083	BMI	C	G	0.030	0.005	3.46E-10	39	0.049	0.040	0.221
rs9926784	BMI	C	T	-0.027	0.004	1.85E-10	40	0.050	0.045	0.259

BMI: body mass index. EA: effect allele. OA: other allele. EAF: EA frequency. GX: beta-exposure. GX(SE): standard error of GX. GY: beta-outcome. GY(SE): standard error of GY.

Table S12.Published associations of WHR on PTB

SNP	Exposure	EA	OA	GX	GX(SE)	pval	F	GY	GX(SE)	pval
rs1011731	WHR	A	G	-0.019	0.003	1.10E-08	33	-0.041	0.034	0.224
rs10245353	WHR	A	C	0.027	0.004	1.60E-10	41	-0.022	0.038	0.563
rs10783615	WHR	A	G	-0.035	0.005	7.00E-13	51	0.020	0.045	0.657
rs11048470	WHR	T	G	0.025	0.004	6.30E-12	46	0.010	0.036	0.774
rs1294421	WHR	G	T	0.025	0.003	6.90E-14	54	-0.016	0.034	0.645
rs1316952	WHR	C	T	-0.028	0.005	7.30E-09	33	0.024	0.056	0.669
rs1440372	WHR	C	T	0.021	0.004	7.60E-09	32	-0.014	0.039	0.713
rs1563355	WHR	C	T	0.031	0.004	1.70E-12	50	0.001	0.036	0.973
rs1569135	WHR	G	A	-0.024	0.003	1.00E-12	53	0.102	0.034	0.002
rs16996700	WHR	C	T	-0.021	0.004	1.60E-08	32	-0.004	0.040	0.916
rs2179129	WHR	G	A	-0.021	0.003	1.20E-09	38	-0.030	0.035	0.387
rs2287019	WHR	T	C	-0.026	0.005	4.30E-09	33	-0.072	0.040	0.072
rs2765539	WHR	T	C	0.027	0.004	1.10E-12	50	0.003	0.047	0.944
rs4640244	WHR	G	A	0.021	0.004	3.10E-08	32	0.027	0.034	0.436
rs4715213	WHR	T	C	0.024	0.004	4.00E-08	30	-0.042	0.042	0.315
rs4929927	WHR	G	A	0.020	0.003	7.60E-09	35	-0.069	0.035	0.051
rs929641	WHR	G	A	-0.020	0.003	4.20E-09	37	-0.003	0.035	0.922
rs9860730	WHR	G	A	-0.023	0.004	2.80E-10	41	0.048	0.037	0.192

WHR: waist-to-hip ratio. EA: effect allele. OA: other allele. EAF: EA frequency. GX: beta-exposure. GX(SE): standard error of GX. GY: beta-outcome. GY(SE): standard error of GY.

Table S13. Published associations of PTB on HbA1c

SNP	Exposure	EA	OA	GX	GX(SE)	pval	F	GY	GY(SE)	pval
rs10847937	PTB	C	A	0.176	0.038	3.20E-06	22	0.003	0.002	0.130
rs117050744	PTB	C	T	-0.444	0.098	5.58E-06	21	0.011	0.013	0.400
rs117620244	PTB	T	C	0.361	0.070	2.49E-07	27	-0.007	0.007	0.280
rs118082956	PTB	G	A	0.379	0.085	7.58E-06	20	0.000	0.007	0.970
rs11926030	PTB	G	T	0.417	0.094	8.66E-06	20	-0.016	0.006	0.013
rs13434036	PTB	T	G	-0.446	0.097	4.02E-06	21	0.001	0.004	0.790
rs145653024	PTB	T	C	0.802	0.161	6.46E-07	25	-0.016	0.008	0.042
rs2027204	PTB	A	G	0.159	0.034	3.05E-06	22	0.000	0.002	0.880
rs2395516	PTB	C	T	0.176	0.034	2.20E-07	27	-0.001	0.002	0.430
rs6120438	PTB	A	G	-0.149	0.034	8.88E-06	20	0.002	0.002	0.280
rs6586520	PTB	T	C	0.155	0.034	4.32E-06	21	-0.001	0.002	0.350
rs6708458	PTB	G	A	0.272	0.058	2.29E-06	22	0.003	0.002	0.120
rs72836185	PTB	C	T	-0.730	0.164	8.41E-06	20	0.005	0.004	0.140
rs73579425	PTB	T	C	0.418	0.094	9.40E-06	20	-0.007	0.005	0.190
rs75686985	PTB	A	C	-0.160	0.035	5.03E-06	21	0.001	0.002	0.720

PTB: pulmonary tuberculosis. EA: effect allele. OA: other allele. EAF: EA frequency. GX: beta-exposure. GX(SE): standard error of GX. GY: beta-outcome. GY(SE): standard error of GY.

Table S14. Published associations of PTB on FI

SNP	Exposure	EA	OA	GX	GX(SE)	pval	F	GY	GY(SE)	pval
rs10847937	PTB	C	A	0.176	0.038	3.20E-06	22	0.002	0.003	0.810
rs117050744	PTB	C	T	-0.444	0.098	5.58E-06	21	0.009	0.011	0.230
rs117620244	PTB	T	C	0.361	0.070	2.49E-07	27	-0.006	0.009	0.706
rs118082956	PTB	G	A	0.379	0.085	7.58E-06	20	-0.008	0.009	0.502
rs11926030	PTB	G	T	0.417	0.094	8.66E-06	20	0.013	0.011	0.296
rs13434036	PTB	T	G	-0.446	0.097	4.02E-06	21	-0.003	0.006	0.359
rs145653024	PTB	T	C	0.802	0.161	6.46E-07	25	0.071	0.029	0.020
rs2027204	PTB	A	G	0.159	0.034	3.05E-06	22	-0.003	0.002	0.110
rs2395516	PTB	C	T	0.176	0.034	2.20E-07	27	0.002	0.003	0.734
rs6120438	PTB	A	G	-0.149	0.034	8.88E-06	20	0.001	0.002	0.657
rs62201868	PTB	A	G	-2.155	0.487	9.75E-06	20	0.018	0.012	0.206
rs6586520	PTB	T	C	0.155	0.034	4.32E-06	21	-0.002	0.002	0.616
rs6708458	PTB	G	A	0.272	0.058	2.29E-06	22	0.003	0.003	0.310
rs72738736	PTB	T	G	0.157	0.035	8.09E-06	20	-0.001	0.002	0.292
rs72836185	PTB	C	T	-0.730	0.164	8.41E-06	20	0.000	0.006	0.653
rs73579425	PTB	T	C	0.418	0.094	9.40E-06	20	0.012	0.011	0.233
rs75686985	PTB	A	C	-0.160	0.035	5.03E-06	21	0.007	0.008	0.349
rs79513808	PTB	T	C	0.331	0.073	5.51E-06	21	0.001	0.005	0.627

PTB: pulmonary tuberculosis. EA: effect allele. OA: other allele. EAF: EA frequency. GX: beta-exposure. GX(SE): standard error of GX. GY: beta-outcome. GY(SE): standard error of GY.

Table S15. Published associations of PTB on LDL-C

SNP	Exposure	EA	OA	GX	GX(SE)	pval	F	GY	GY(SE)	pval
rs10847937	PTB	C	A	0.176	0.038	3.20E-06	22	0.008	0.006	0.226
rs13434036	PTB	T	G	-0.446	0.097	4.02E-06	21	-0.012	0.028	0.924
rs2027204	PTB	A	G	0.159	0.034	3.05E-06	22	0.004	0.005	0.681
rs6708458	PTB	G	A	0.272	0.058	2.29E-06	22	0.006	0.008	0.542
rs72738736	PTB	T	G	0.157	0.035	8.09E-06	20	0.005	0.006	0.363

PTB: pulmonary tuberculosis. EA: effect allele. OA: other allele. EAF: EA frequency. GX: beta-exposure. GX(SE): standard error of GX. GY: beta-outcome. GY(SE): standard error of GY.

Table S16. Published associations of PTB on HDL-C

SNP	Exposure	EA	OA	GX	GX(SE)	pval	F	GY	GY(SE)	pval
rs10847937	PTB	C	A	0.176	0.038	3.20E-06	22	0.002	0.006	0.917
rs13434036	PTB	T	G	-0.446	0.097	4.02E-06	21	0.039	0.025	0.262
rs2027204	PTB	A	G	0.159	0.034	3.05E-06	22	-0.002	0.005	0.853
rs6708458	PTB	G	A	0.272	0.058	2.29E-06	22	-0.001	0.007	0.637
rs72738736	PTB	T	G	0.157	0.035	8.09E-06	20	-0.008	0.005	0.150

PTB: pulmonary tuberculosis. EA: effect allele. OA: other allele. EAF: EA frequency. GX: beta-exposure. GX(SE): standard error of GX. GY: beta-outcome. GY(SE): standard error of GY.

Table S17. Published associations of PTB on TG

SNP	Exposure	EA	OA	GX	GX(SE)	pval	F	GY	GY(SE)	pval
rs10847937	PTB	C	A	0.176	0.038	3.20E-06	22	0.003	0.006	0.676
rs13434036	PTB	T	G	-0.446	0.097	4.02E-06	21	-0.025	0.025	0.420
rs2027204	PTB	A	G	0.159	0.034	3.05E-06	22	0.000	0.005	0.917
rs6708458	PTB	G	A	0.272	0.058	2.29E-06	22	0.000	0.007	0.688
rs72738736	PTB	T	G	0.157	0.035	8.09E-06	20	0.007	0.005	0.368

PTB: pulmonary tuberculosis. EA: effect allele. OA: other allele. EAF: EA frequency. GX: beta-exposure. GX(SE): standard error of GX. GY: beta-outcome. GY(SE): standard error of GY.

Table S18. Published associations of PTB on TC

SNP	Exposure	EA	OA	GX	GX(SE)	pval	F	GY	GY(SE)	pval
rs10847937	PTB	C	A	0.176	0.038	3.20E-06	22	0.009	0.006	0.190
rs13434036	PTB	T	G	-0.446	0.097	4.02E-06	21	0.004	0.027	0.548
rs2027204	PTB	A	G	0.159	0.034	3.05E-06	22	0.002	0.005	0.790
rs6708458	PTB	G	A	0.272	0.058	2.29E-06	22	0.003	0.008	0.860
rs72738736	PTB	T	G	0.157	0.035	8.09E-06	20	0.004	0.005	0.488

PTB: pulmonary tuberculosis. EA: effect allele. OA: other allele. EAF: EA frequency. GX: beta-exposure. GX(SE): standard error of GX. GY: beta-outcome. GY(SE): standard error of GY.

Table S19. Published associations of PTB on BMI

SNP	Exposure	EA	OA	GX	GX(SE)	pval	F	GY	GY(SE)	pval
rs10847937	PTB	C	A	0.176	0.038	3.20E-06	22	0.000	0.004	0.928
rs13434036	PTB	T	G	-0.446	0.097	4.02E-06	21	-0.033	0.022	0.131
rs2027204	PTB	A	G	0.159	0.034	3.05E-06	22	0.007	0.004	0.080
rs62201868	PTB	A	G	-2.155	0.487	9.75E-06	20	-0.004	0.042	0.927
rs6708458	PTB	G	A	0.272	0.058	2.29E-06	22	-0.002	0.006	0.739
rs72738736	PTB	T	G	0.157	0.035	8.09E-06	20	-0.007	0.004	0.064

PTB: pulmonary tuberculosis. EA: effect allele. OA: other allele. EAF: EA frequency. GX: beta-exposure. GX(SE): standard error of GX. GY: beta-outcome. GY(SE): standard error of GY.

Table S20. Published associations of PTB on WHR

SNP	Exposure	EA	OA	GX	GX(SE)	pval	F	GY	GY(SE)	pval
rs10847937	PTB	C	A	0.176	0.038	3.20E-06	22	0.005	0.005	0.270
rs13434036	PTB	T	G	-0.446	0.097	4.02E-06	21	-0.056	0.024	0.020
rs2027204	PTB	A	G	0.159	0.034	3.05E-06	22	0.004	0.004	0.340
rs6708458	PTB	G	A	0.272	0.058	2.29E-06	22	-0.009	0.006	0.170
rs72738736	PTB	T	G	0.157	0.035	8.09E-06	20	-0.001	0.004	0.890

PTB: pulmonary tuberculosis. EA: effect allele. OA: other allele. EAF: EA frequency. GX: beta-exposure. GX(SE): standard error of GX. GY: beta-outcome. GY(SE): standard error of GY.

Figure S1. T1DM-associated SNPs with risk of PTB

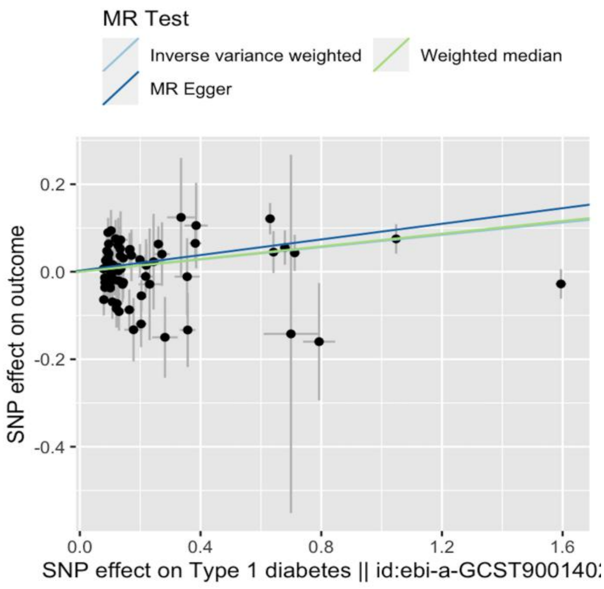

A. Scatter plot of causal effect of T1DM on PTB

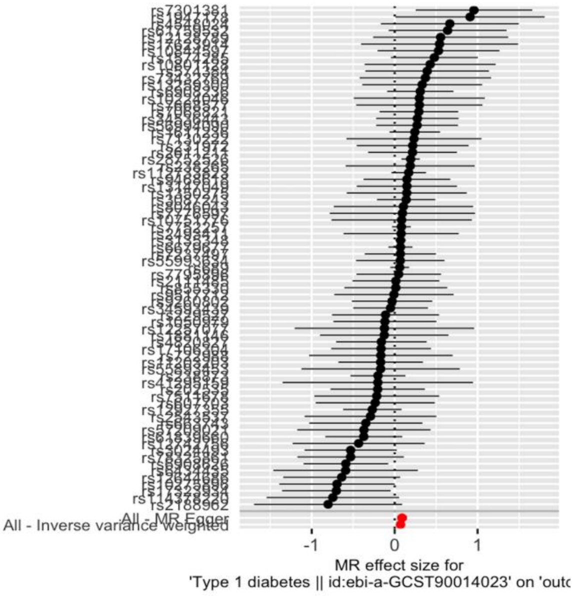

B. Forest plot of causal effect of T1DM on PTB

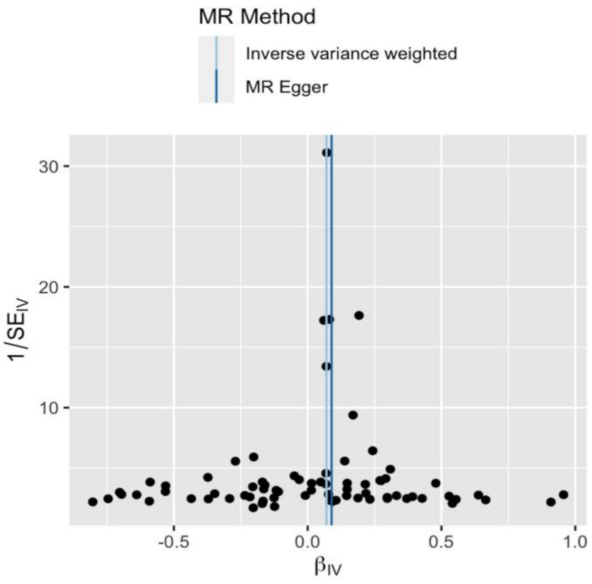

C. Funnel plot of causal effect of T1DM on PTB

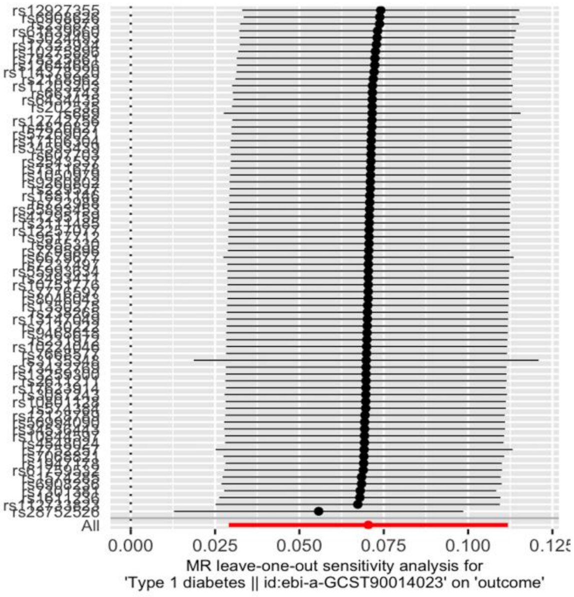

D. Leave-one-out analysis of T1DM on PTB

Figure S2. PTB-associated SNPs with risk of T1DM

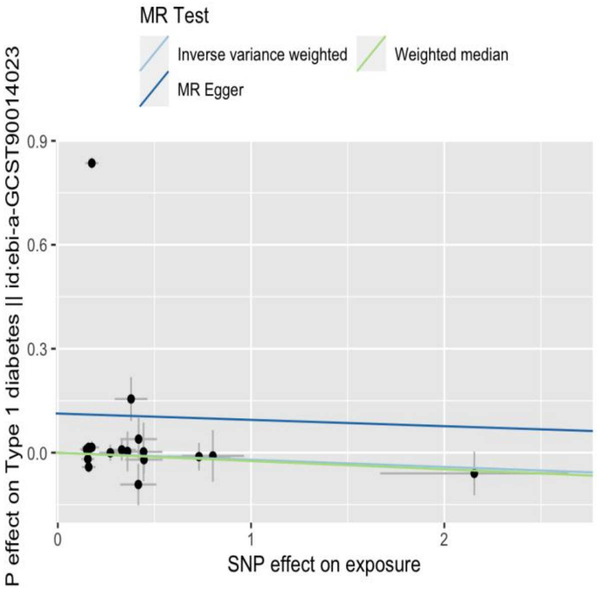

A. Scatter plot of causal effect of PTB on T1DM

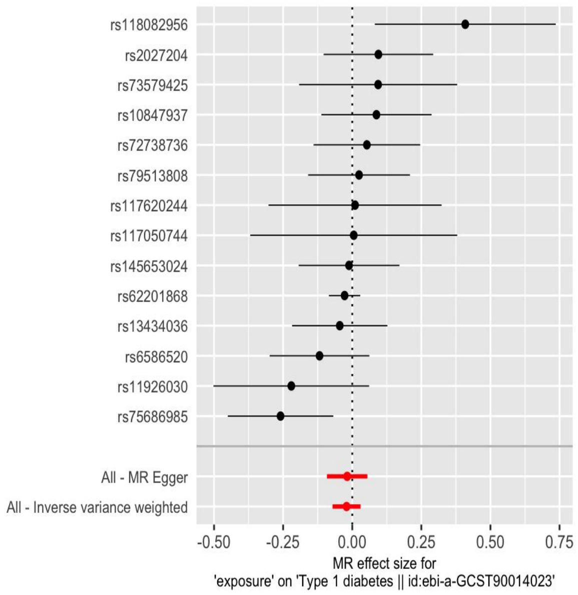

B. Forest plot of causal effect of PTB on T1DM

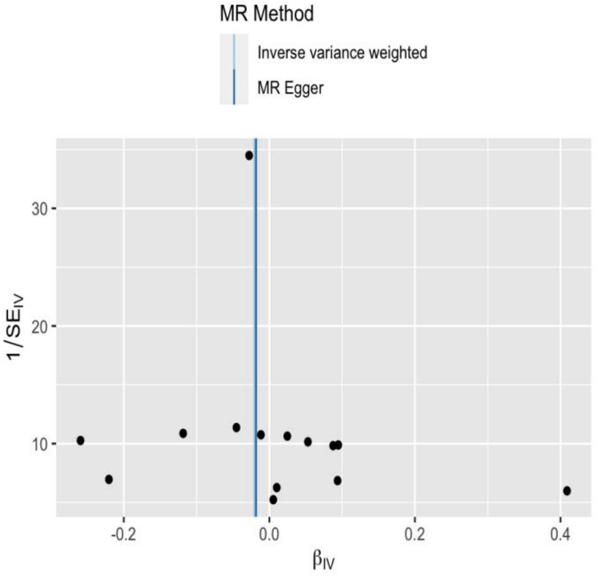

C. Funnel plot of causal effect of PTB on T1DM

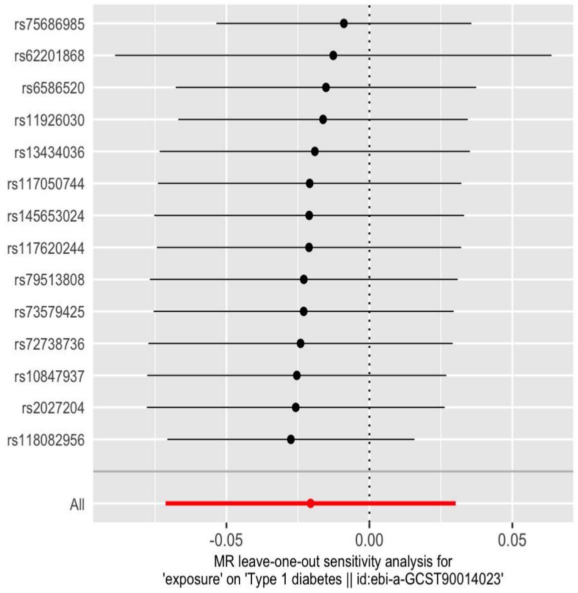

D. Leave-one-out analysis of PTB on T1DM

Figure S3. FBG-associated SNPs with risk of PTB

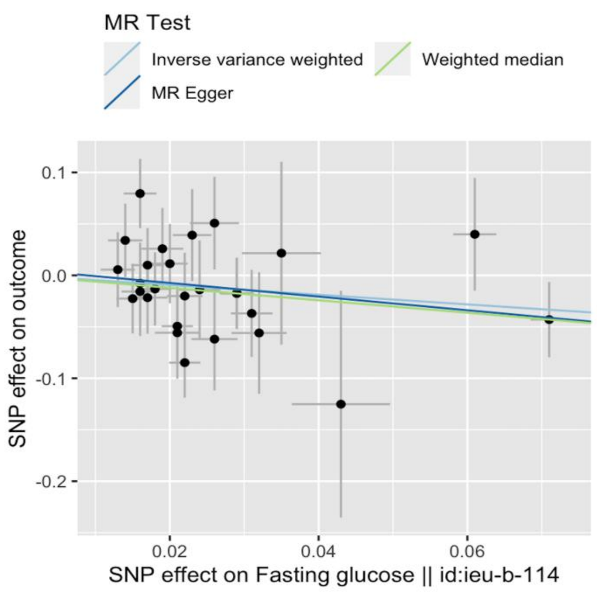

A. Scatter plot of causal effect of FBG on PTB

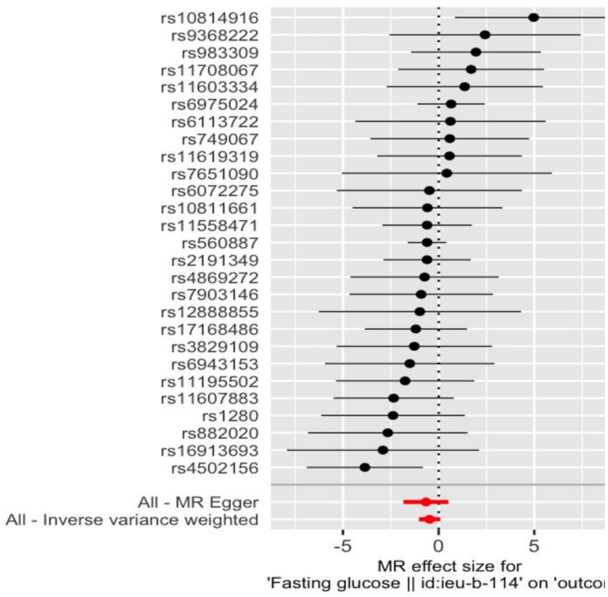

B. Forest plot of causal effect of FBG on PTB

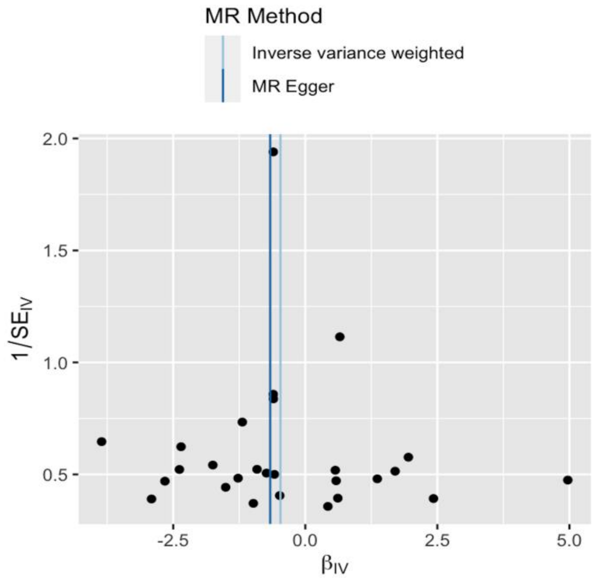

C. Funnel plot of causal effect of FBG on PTB

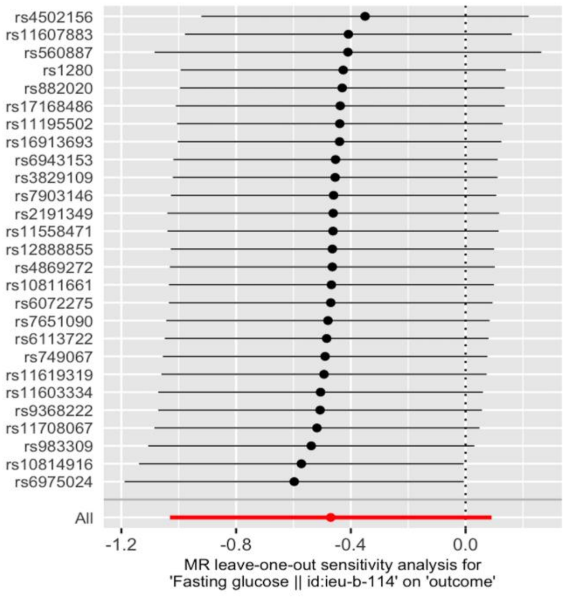

D. Leave-one-out analysis of FBG on PTB

Figure S4. HAb1c-associated SNPs with risk of PTB

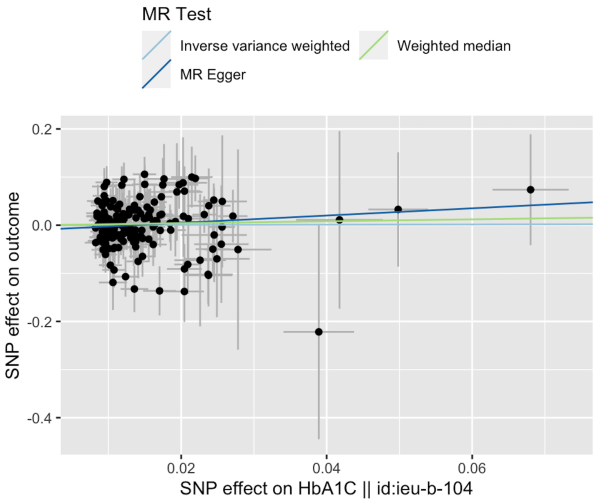

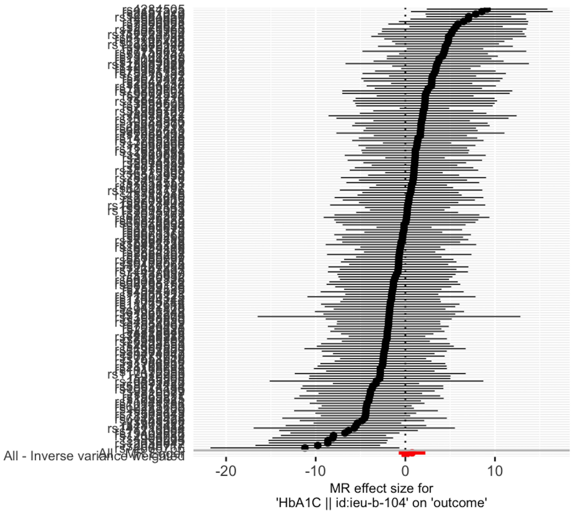

A. Scatter plot of causal effect of HAb1c on PTB

B. Forest plot of causal effect of HAb1c on PTB

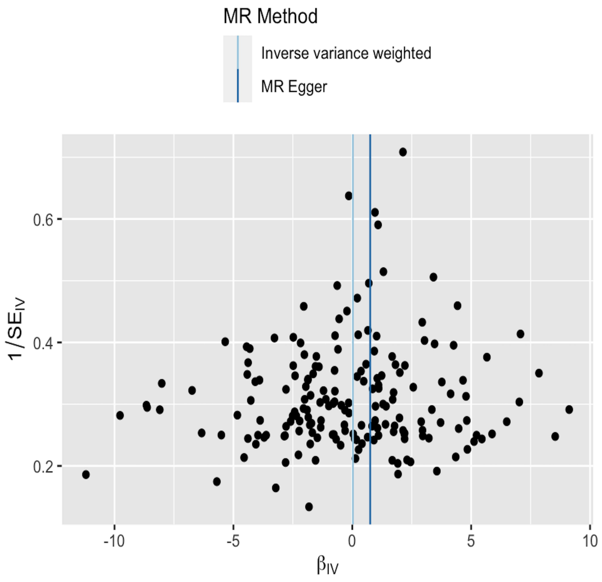

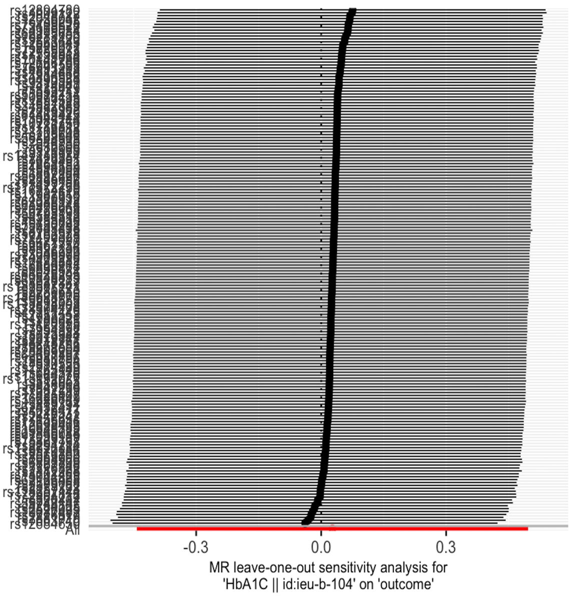

C. Funnel plot of causal effect of HAb1c on PTB

D. Leave-one-out analysis of HAb1c on PTB

Figure S5. FI-associated SNPs with risk of PTB

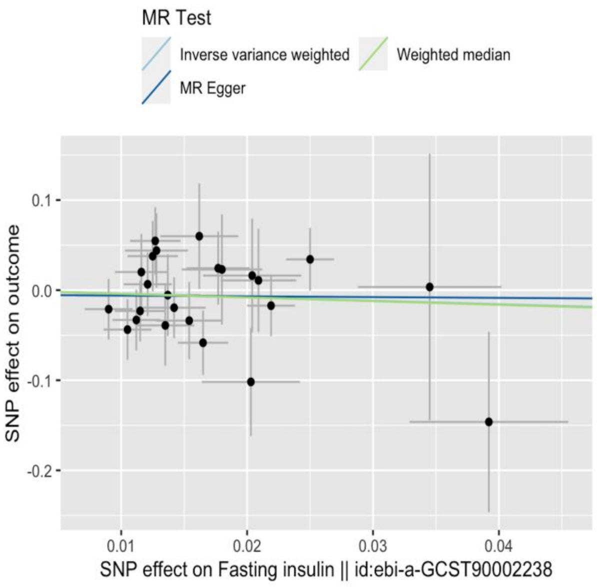

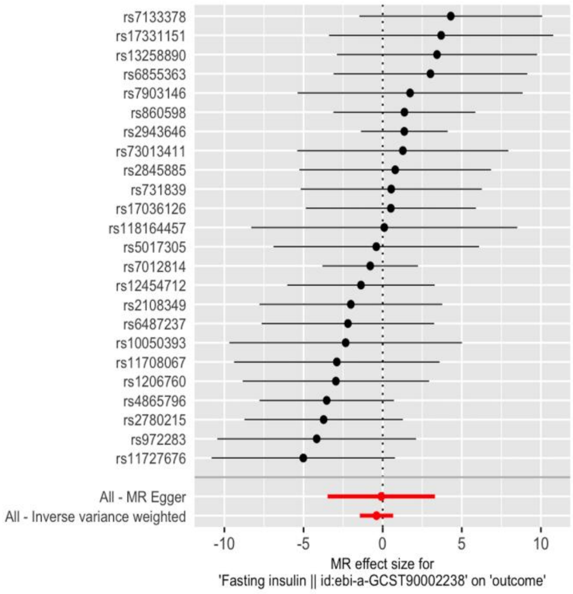

A. Scatter plot of causal effect of FI on PTB

B. Forest plot of causal effect of FI on PTB

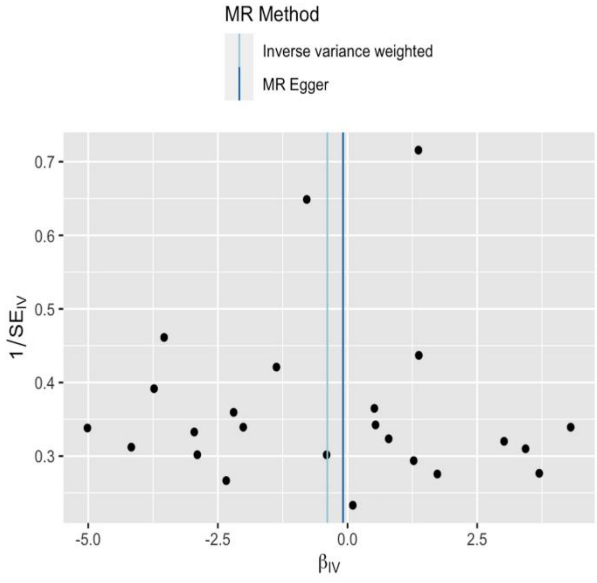

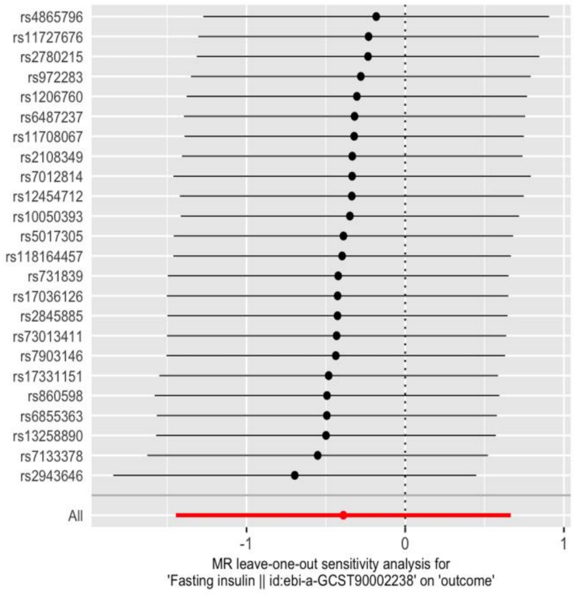

C. Funnel plot of causal effect of FI on PTB

D. Leave-one-out analysis of FI on PTB

Figure S6. LDL-associated SNPs with risk of PTB

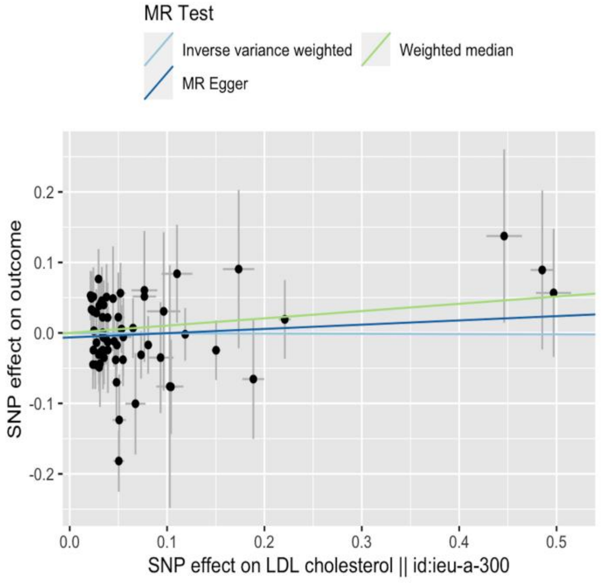

A. Scatter plot of causal effect of LDL on PTB

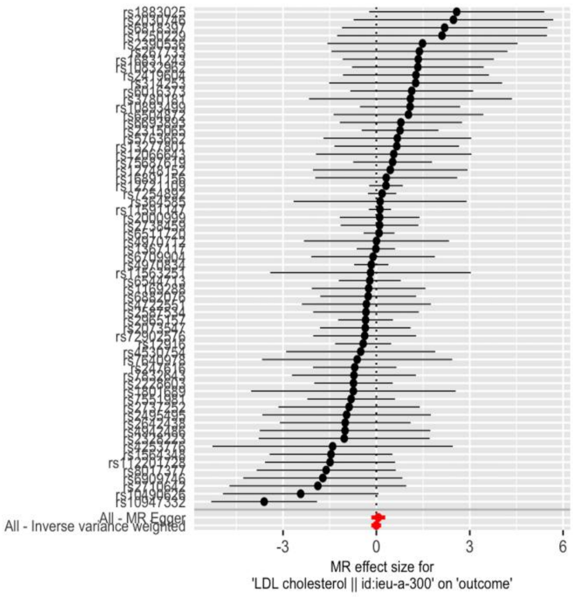

B. Forest plot of causal effect of LDL on PTB

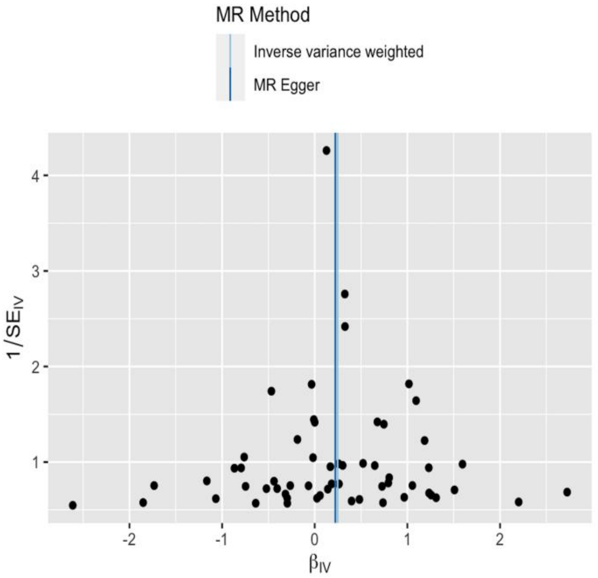

C. Funnel plot of causal effect of LDL on PTB

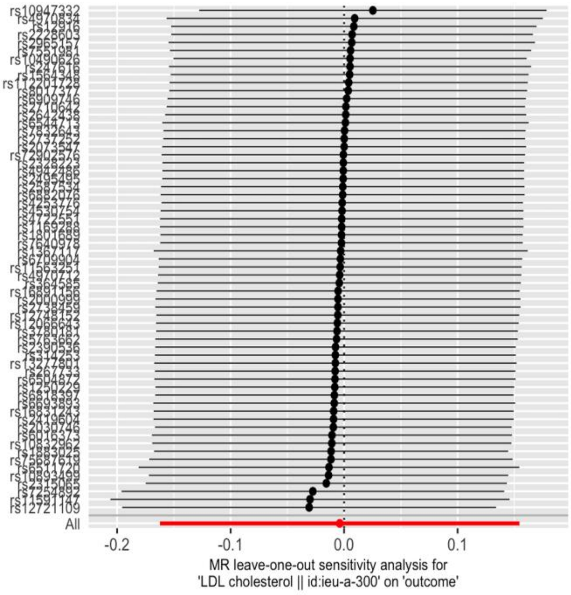

D. Leave-one-out analysis of LDL on PTB

Figure S7. HDL-associated SNPs with risk of PTB

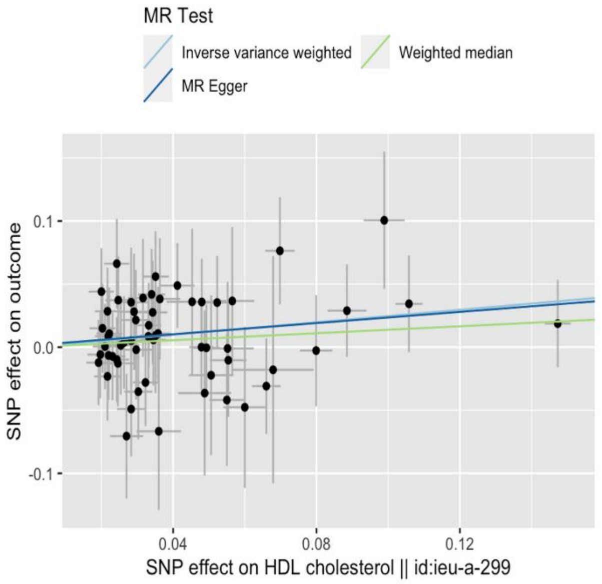

A. Scatter plot of causal effect of HDL on PTB

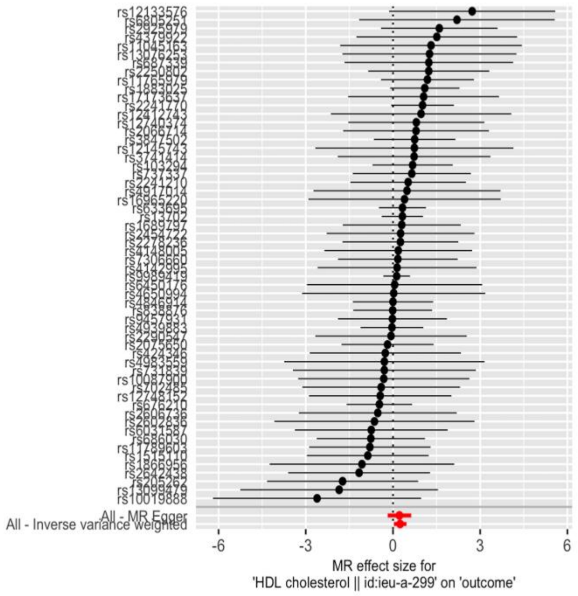

B. Forest plot of causal effect of HDL on PTB

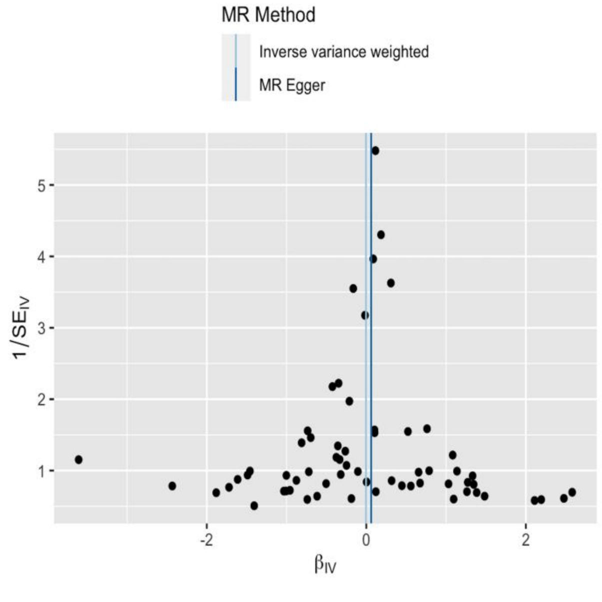

C. Funnel plot of causal effect of HDL on PTB

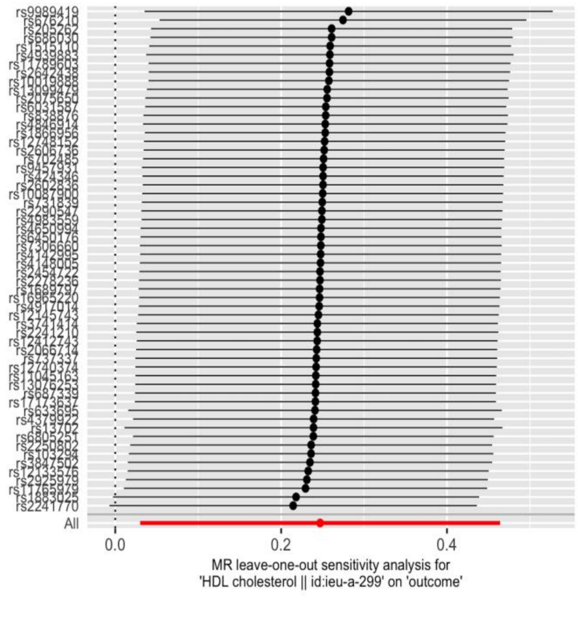

D. Leave-one-out analysis of HDL on PTB

Figure S8. TG-associated SNPs with risk of PTB

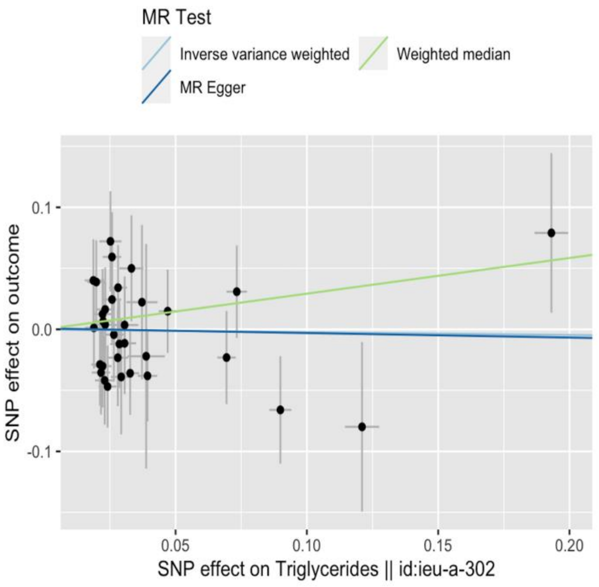

A. Scatter plot of causal effect of TG on PTB

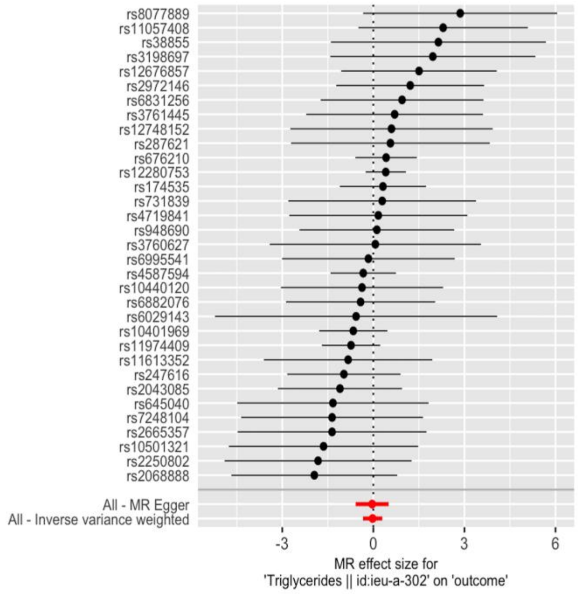

B. Forest plot of causal effect of TG on PTB

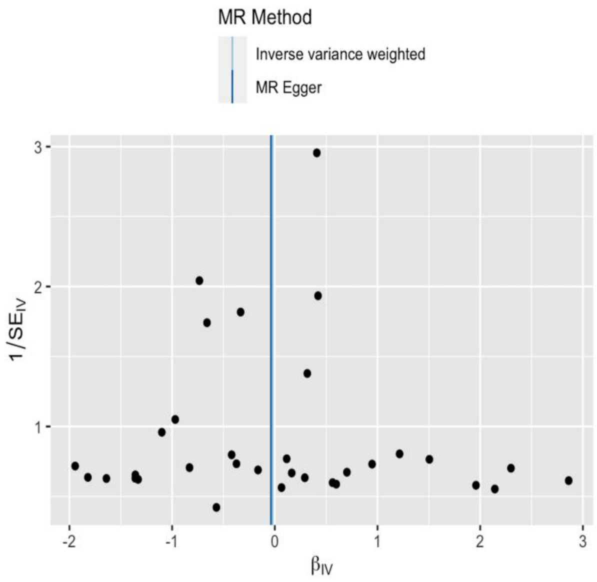

C. Funnel plot of causal effect of TG on PTB

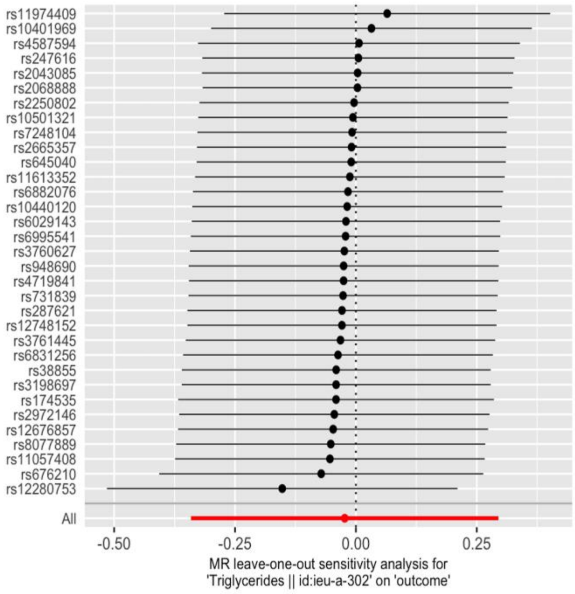

D. Leave-one-out analysis of TG on PTB

Figure S9. TC-associated SNPs with risk of PTB

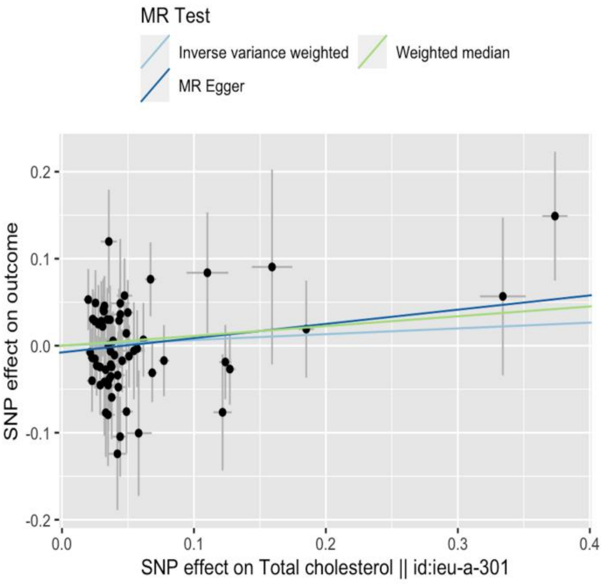

A. Scatter plot of causal effect of TC on PTB

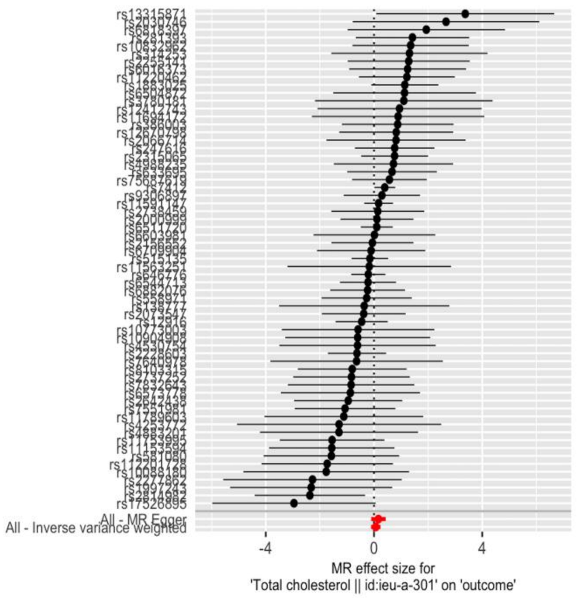

B. Forest plot of causal effect of TC on PTB

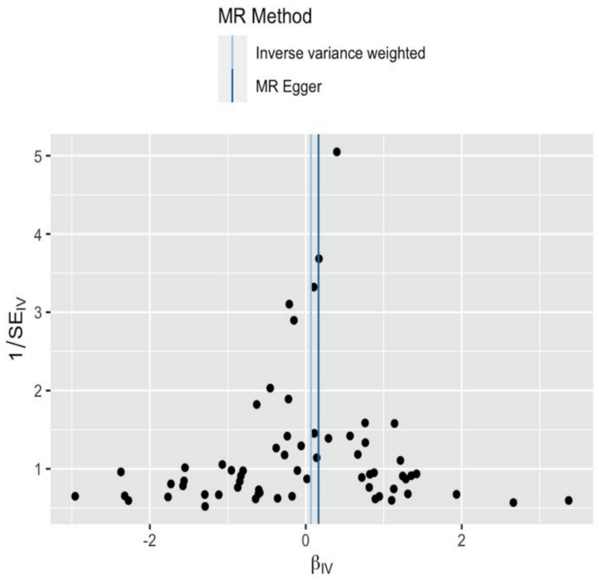

C. Funnel plot of causal effect of TC on PTB

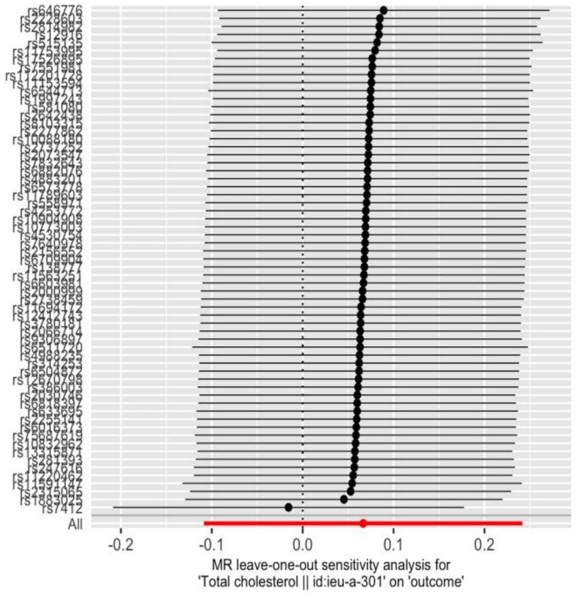

D. Leave-one-out analysis of TC on PTB

Figure S10. BMI-associated SNPs with risk of PTB

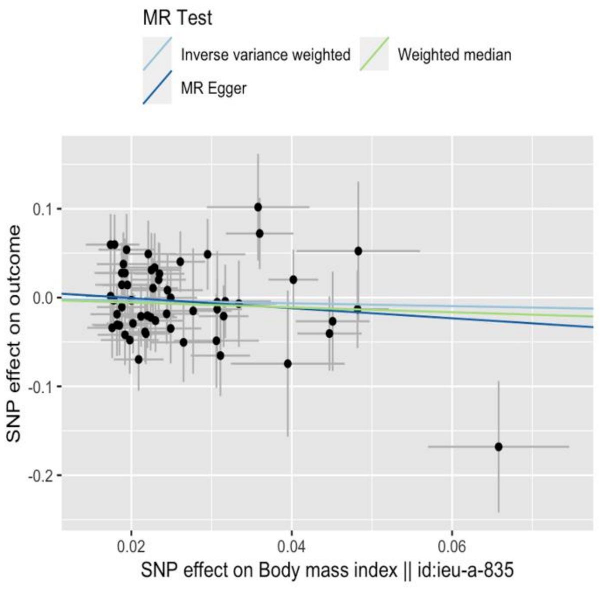

A. Scatter plot of causal effect of BMI on PTB

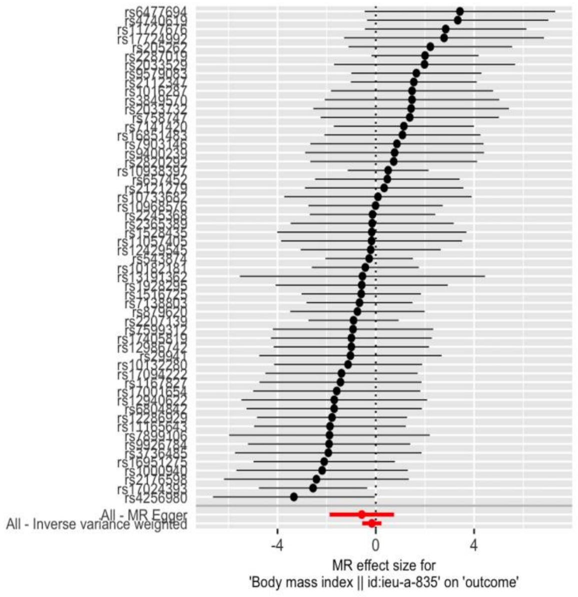

B. Forest plot of causal effect of BMI on PTB

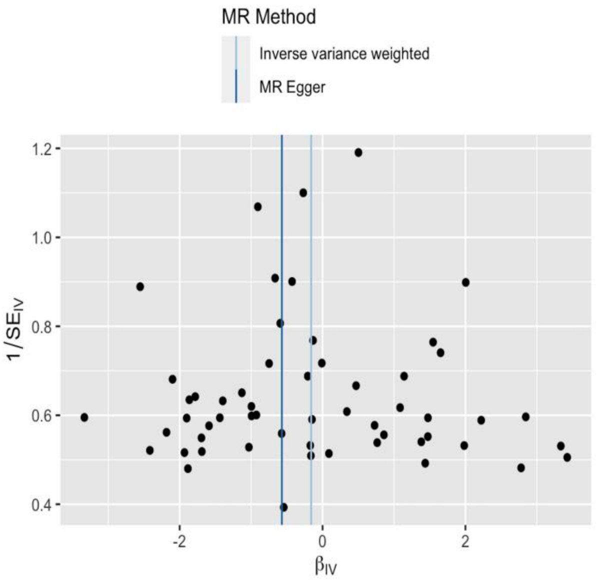

C. Funnel plot of causal effect of BMI on PTB

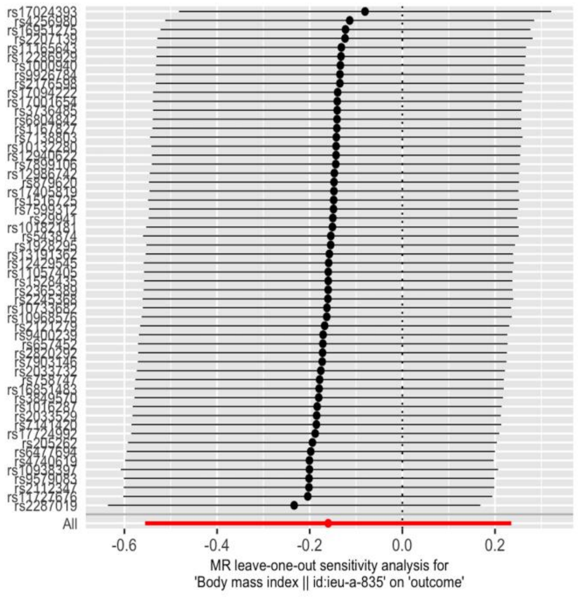

D. Leave-one-out analysis of BMI on PTB

Figure S11. WHR-associated SNPs with risk of PTB

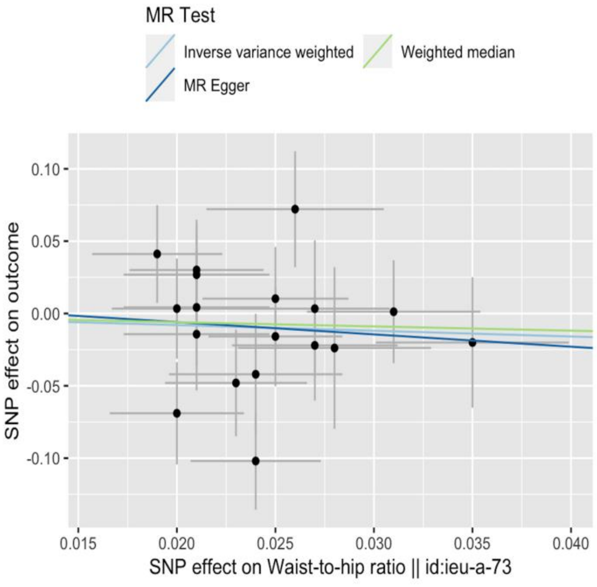

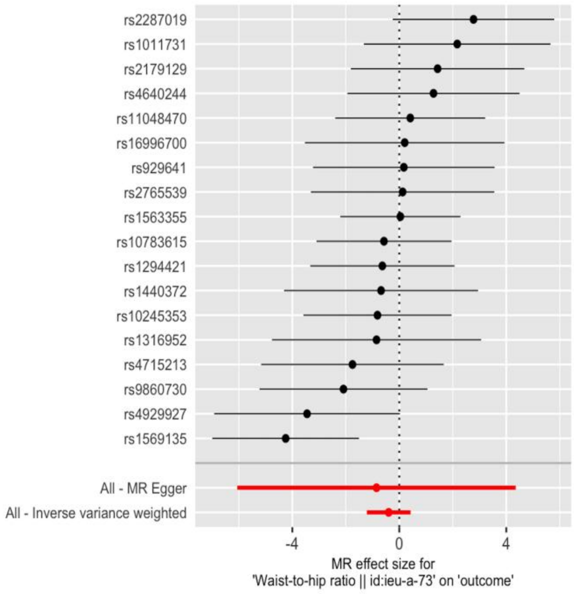

A. Scatter plot of causal effect of WHR on PTB

B. Forest plot of causal effect of WHR on PTB

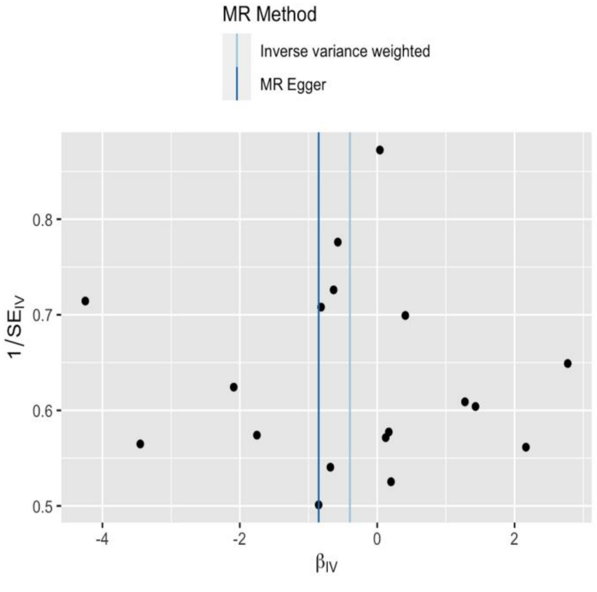

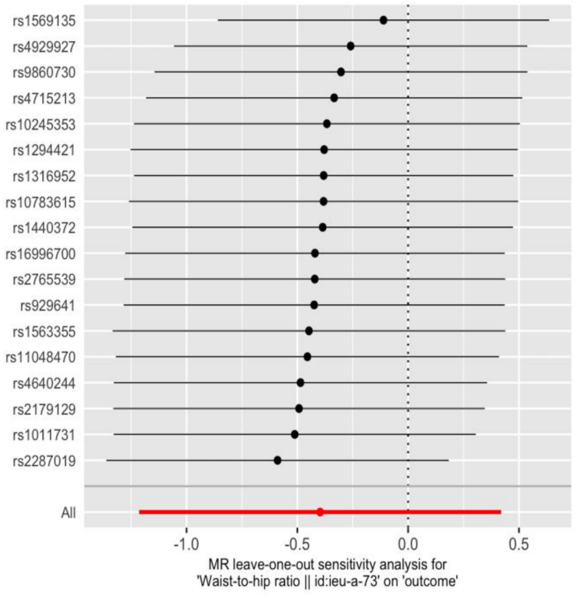

C. Funnel plot of causal effect of WHR on PTB

D. Leave-one-out analysis of WHR on PTB

Figure S12. PTB-associated SNPs with risk of HbA1c

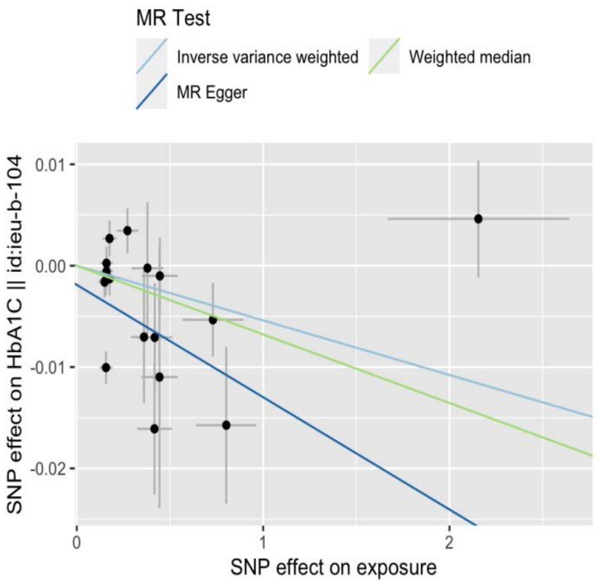

A. Scatter plot of causal effect of PTB on HbA1c

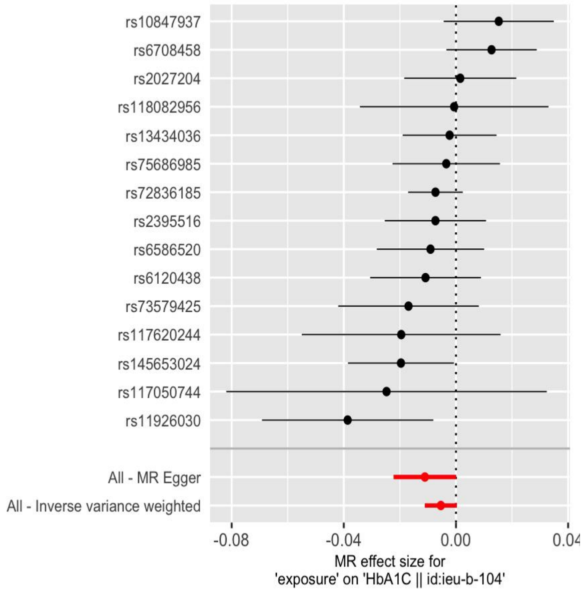

B. Forest plot of causal effect of PTB on HbA1c

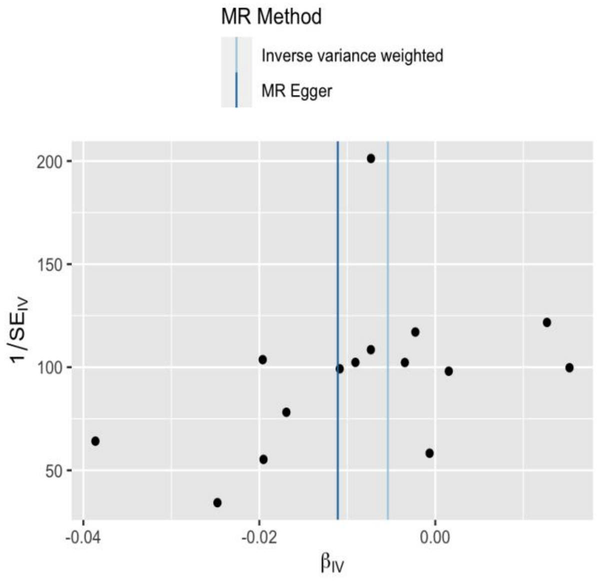

C. Funnel plot of causal effect of PTB on HbA1c

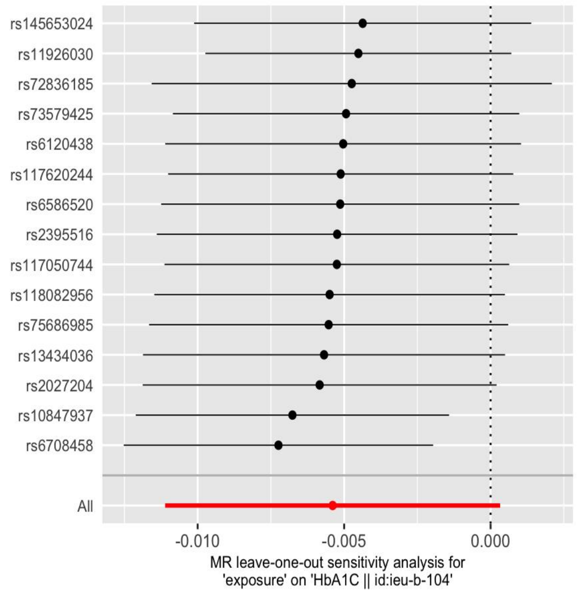

D. Leave-one-out analysis of PTB on HbA1c

Figure S13. PTB-associated SNPs with risk of FI

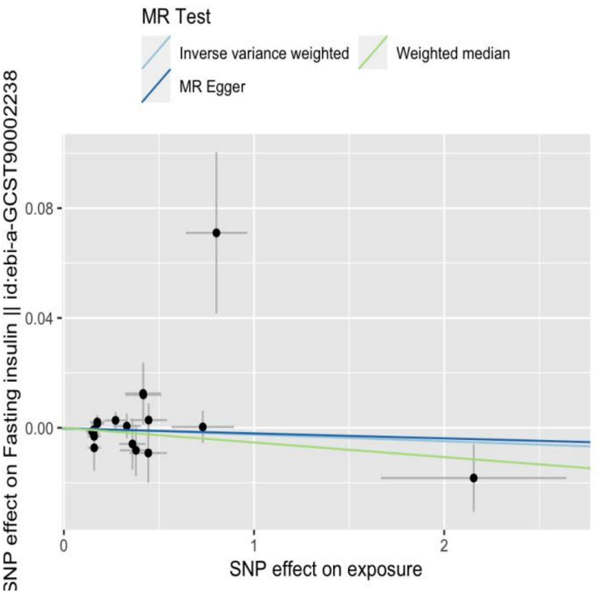

A. Scatter plot of causal effect of PTB on FI

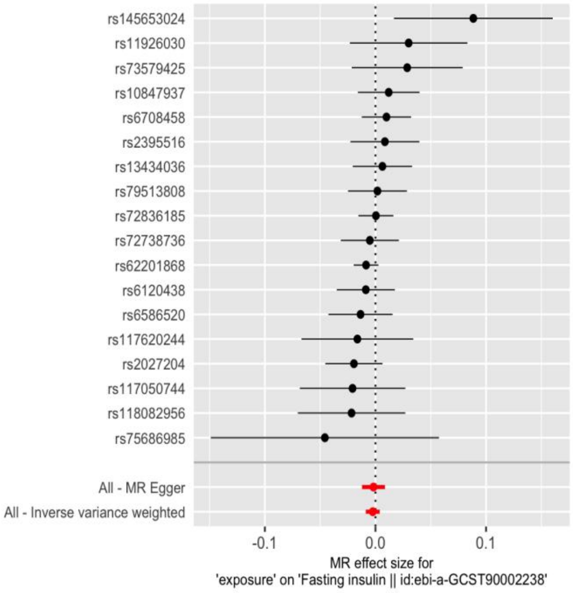

B. Forest plot of causal effect of PTB on FI

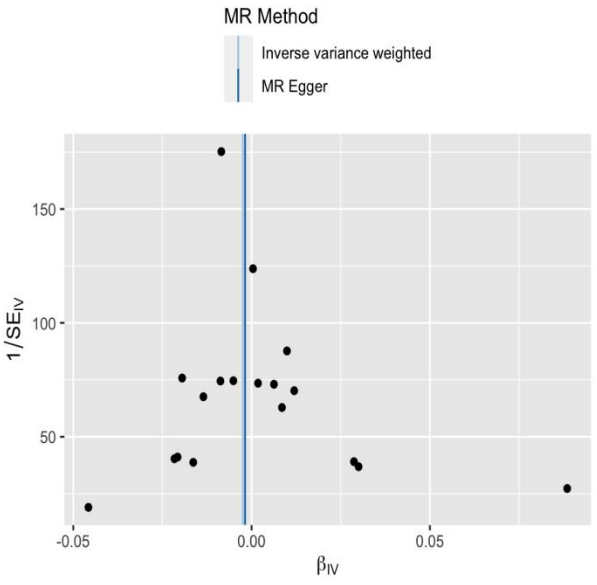

C. Funnel plot of causal effect of PTB on FI

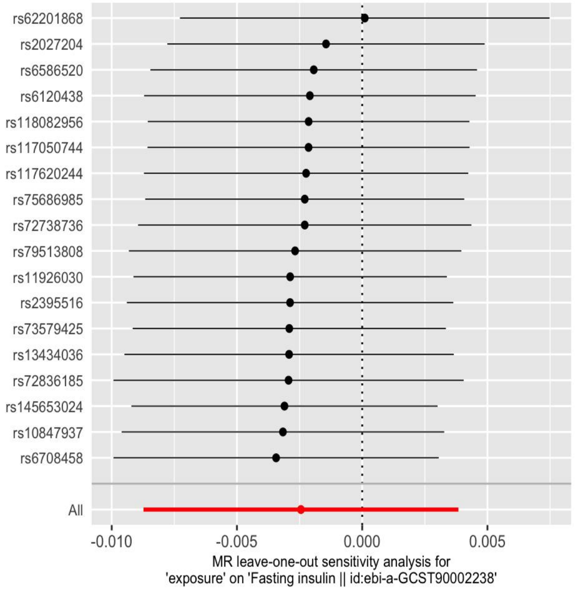

D. Leave-one-out analysis of PTB on FI

Figure S14. PTB-associated SNPs with risk of LDL-C

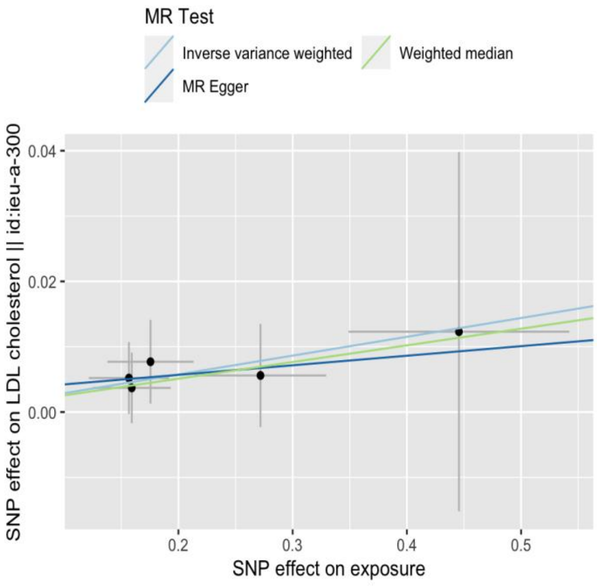

A. Scatter plot of causal effect of PTB on LDL-C

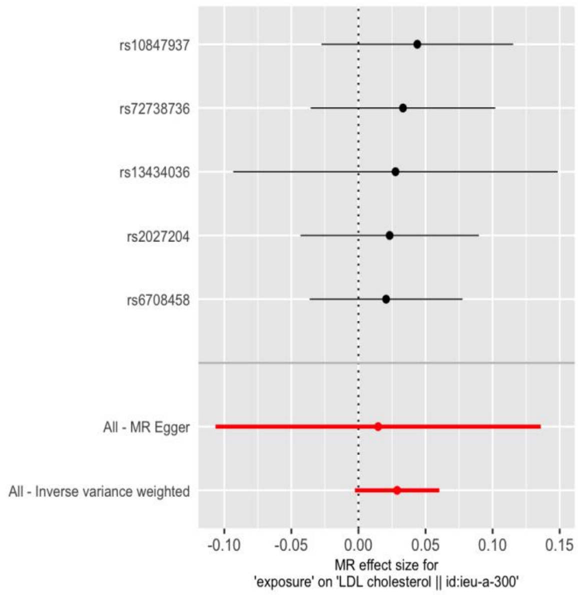

B. Forest plot of causal effect of PTB on LDL-C

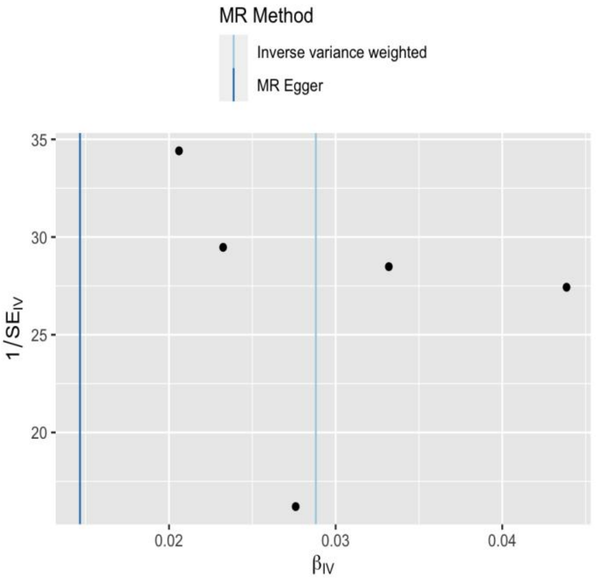

C. Funnel plot of causal effect of PTB on LDL-C

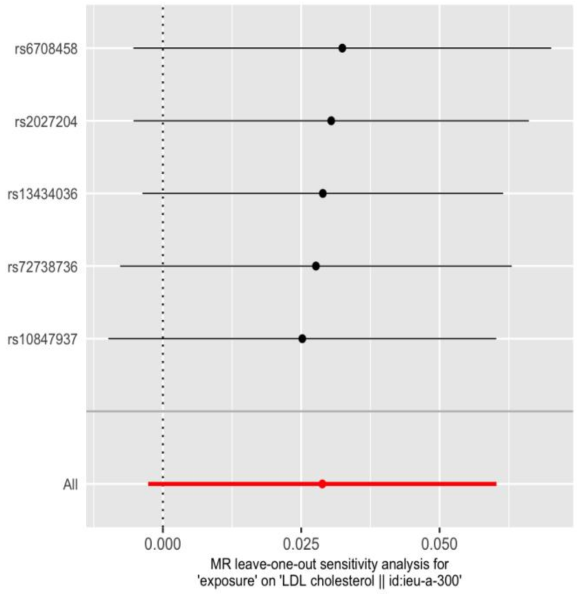

D. Leave-one-out analysis of PTB on LDL-C

Figure S15. PTB-associated SNPs with risk of HDL-C

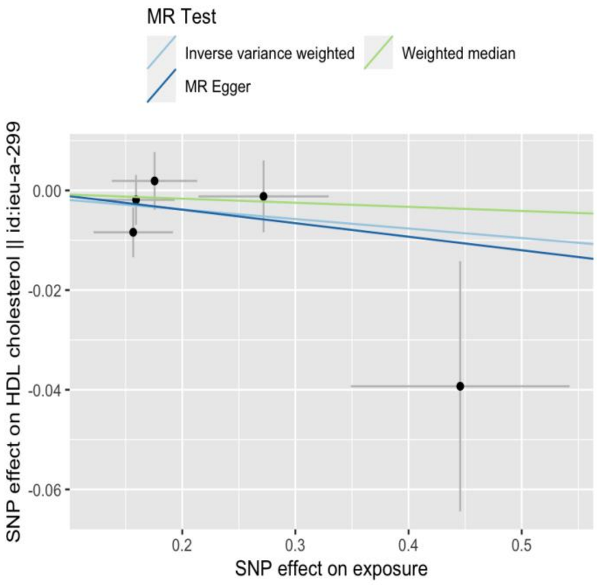

A. Scatter plot of causal effect of PTB on HDL-C

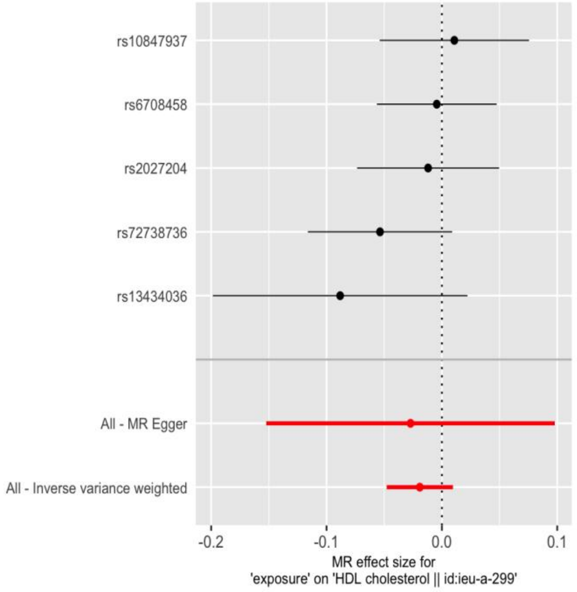

B. Forest plot of causal effect of PTB on HDL-C

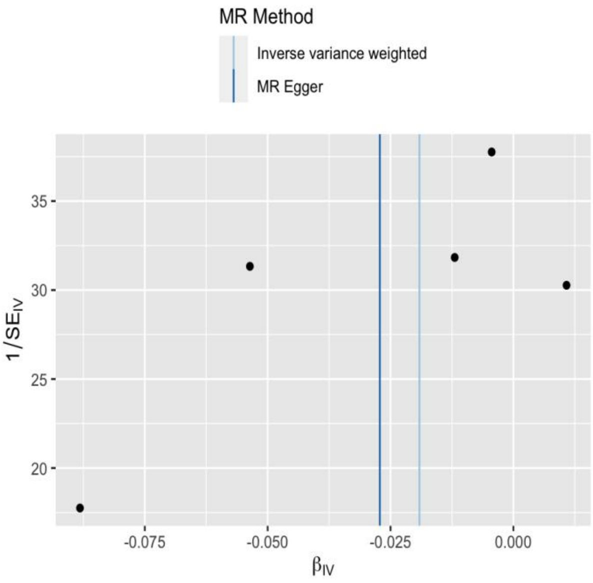

C. Funnel plot of causal effect of PTB on HDL-C

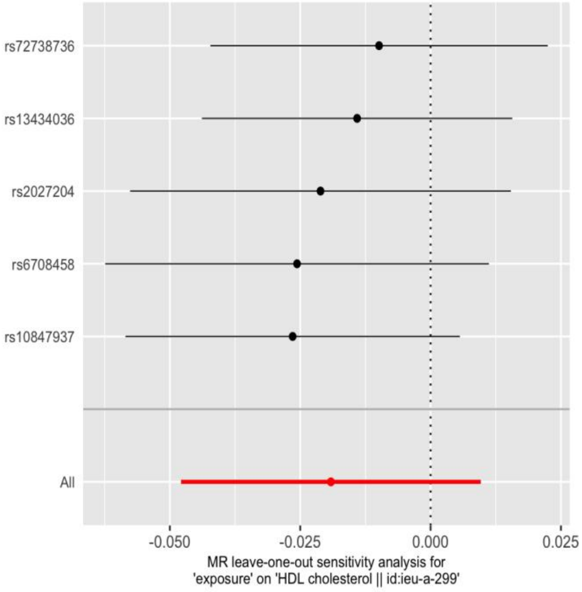

D. Leave-one-out analysis of PTB on HDL-C

Figure S16. PTB-associated SNPs with risk of TG

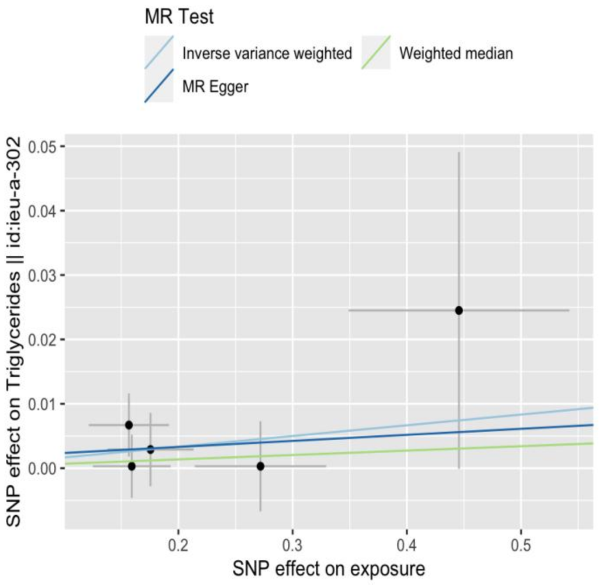

A. Scatter plot of causal effect of PTB on TG

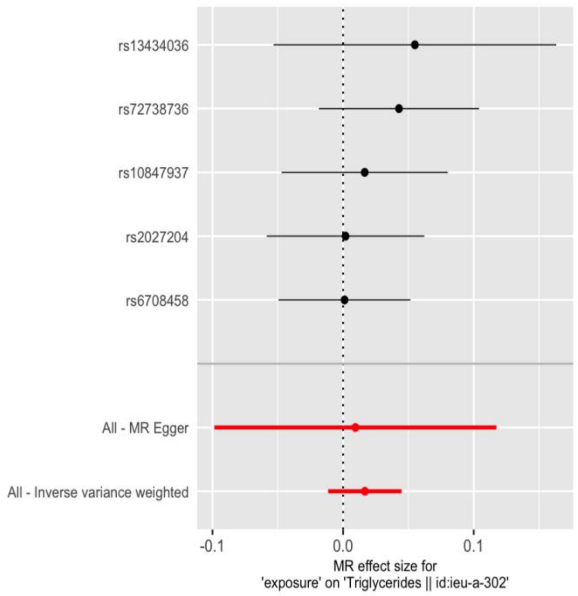

B. Forest plot of causal effect of PTB on TG

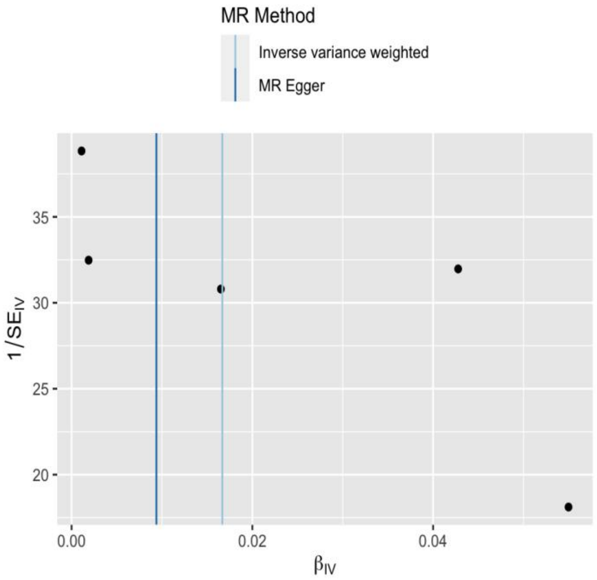

C. Funnel plot of causal effect of PTB on TG

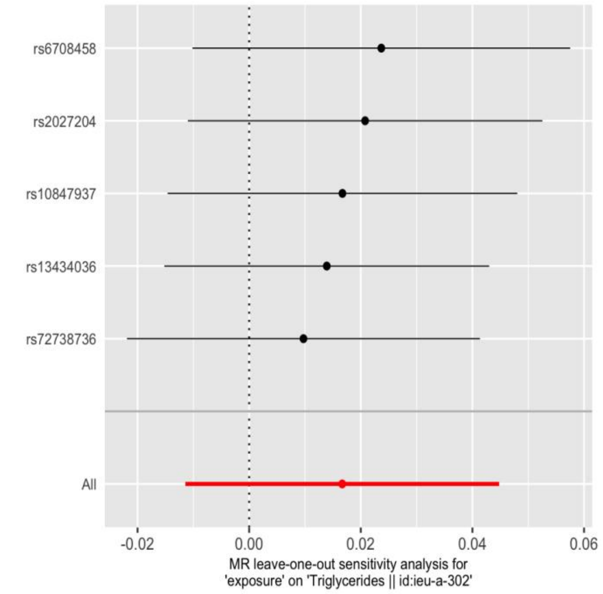

D. Leave-one-out analysis of PTB on TG

Figure S17. PTB-associated SNPs with risk of TC

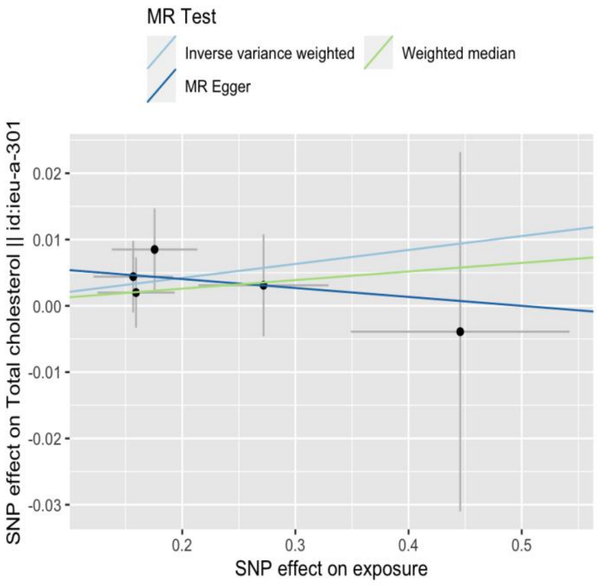

A. Scatter plot of causal effect of PTB on TC

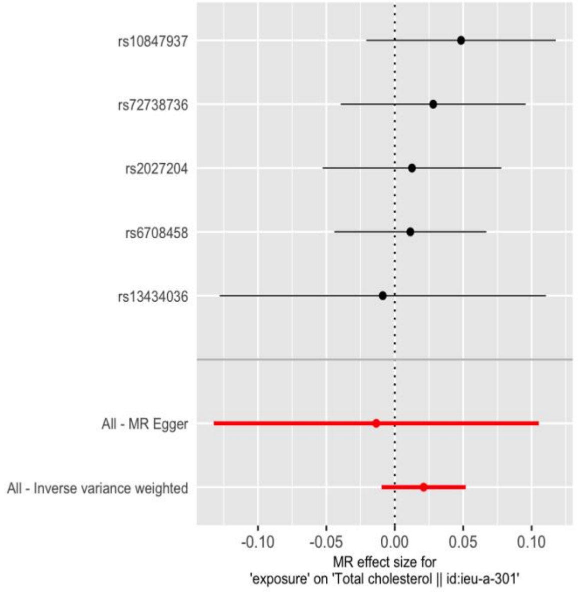

B. Forest plot of causal effect of PTB on TC

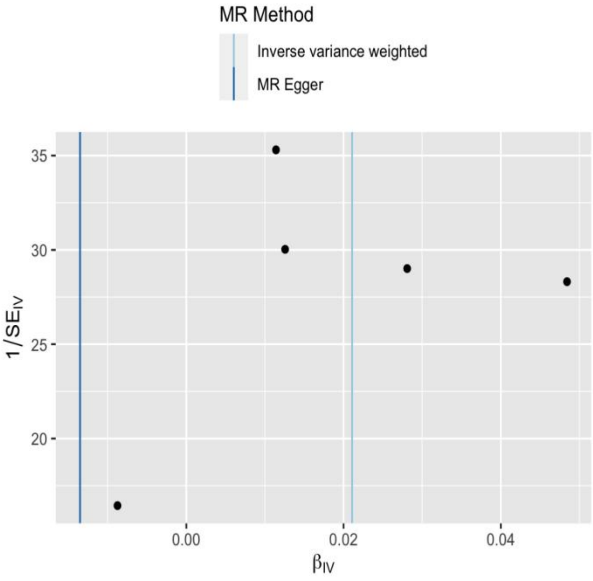

C. Funnel plot of causal effect of PTB on TC

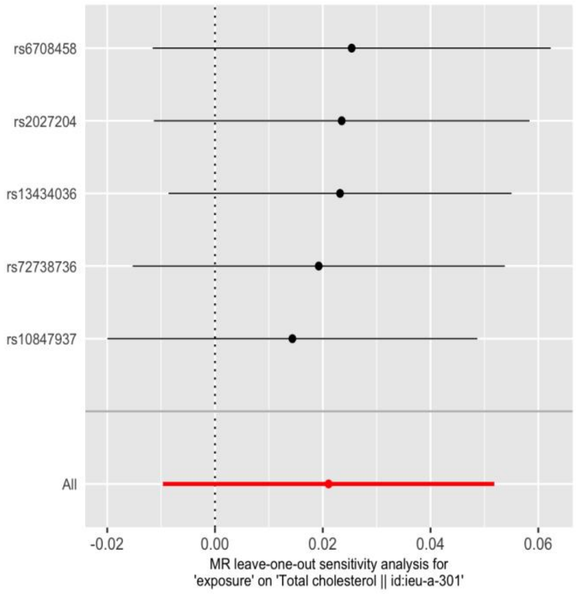

D. Leave-one-out analysis of PTB on TC

Figure S18. PTB-associated SNPs with risk of BMI

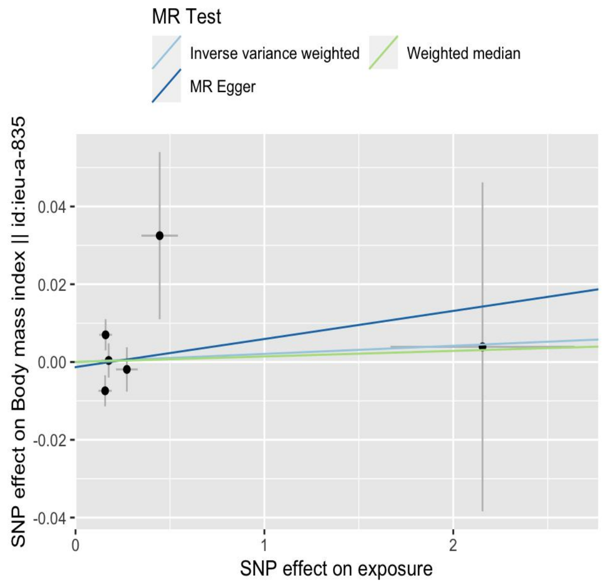

A. Scatter plot of causal effect of PTB on BMI

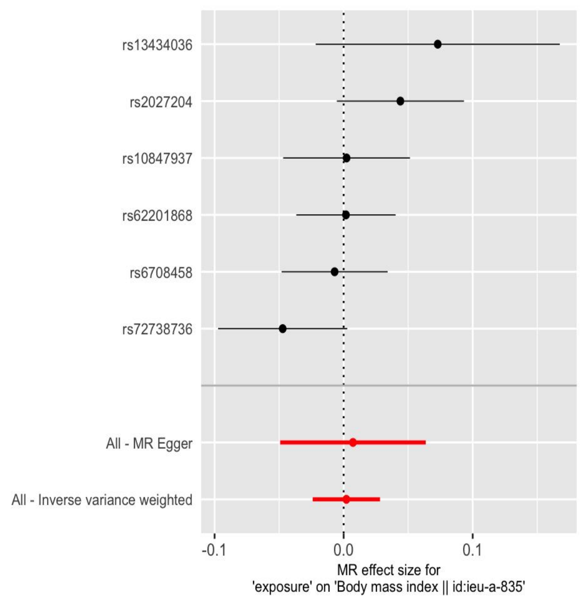

B. Forest plot of causal effect of PTB on BMI

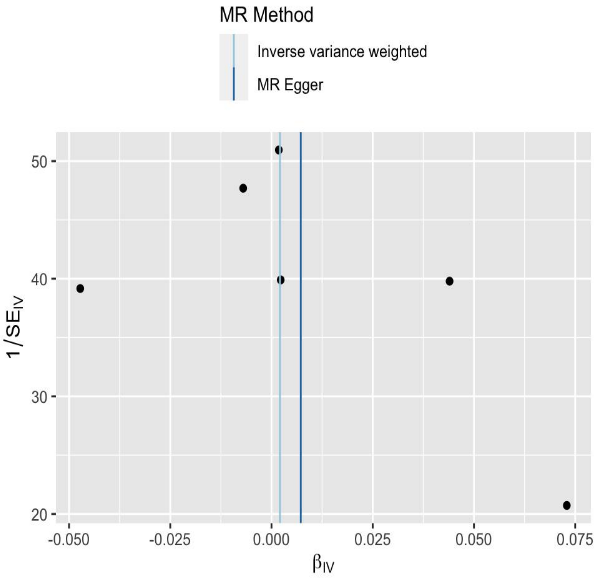

C. Funnel plot of causal effect of PTB on BMI

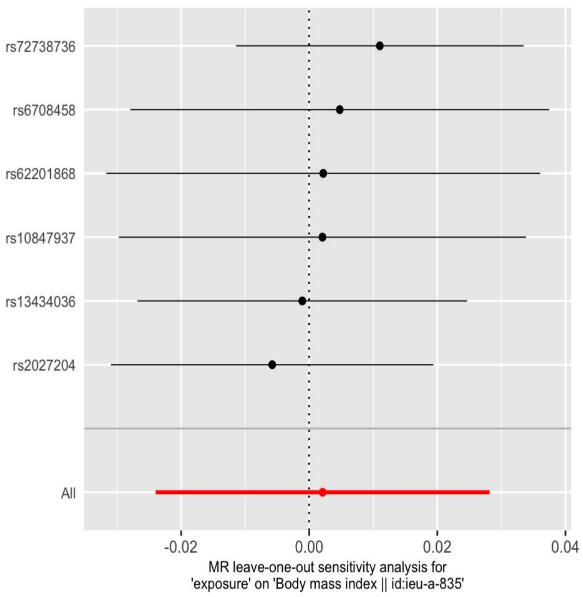

D. Leave-one-out analysis of PTB on BMI

Figure S19. PTB-associated SNPs with risk of WHR

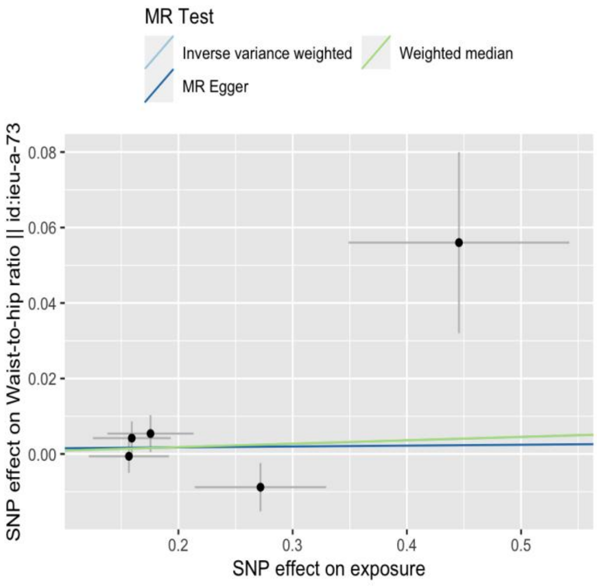

A. Scatter plot of causal effect of PTB on WHR

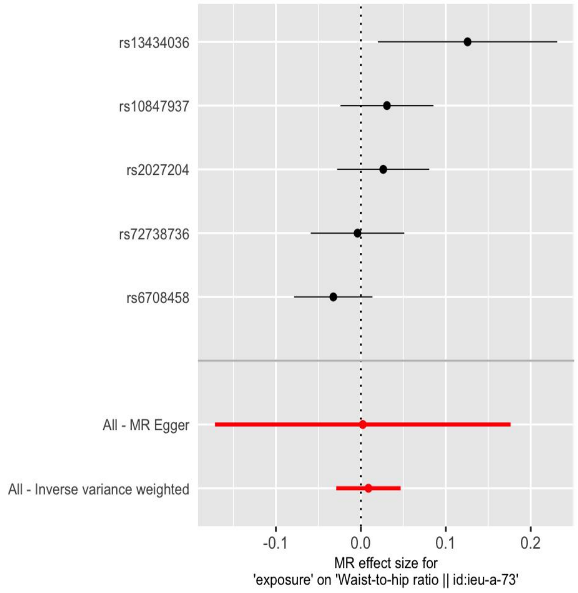

B. Forest plot of causal effect of PTB on WHR

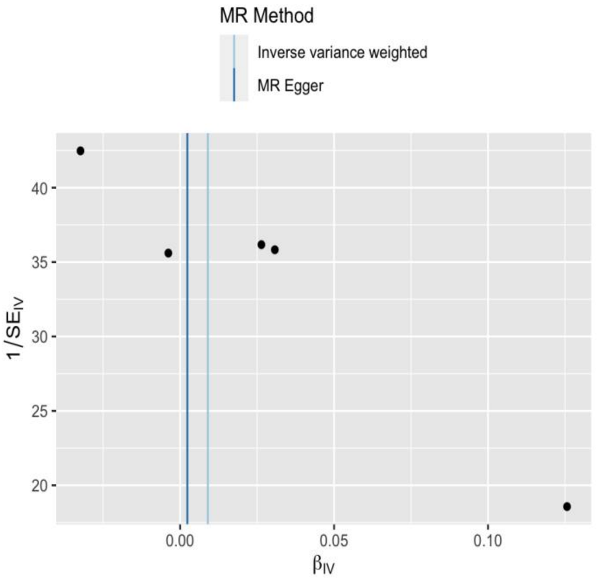

C. Funnel plot of causal effect of PTB on WHR

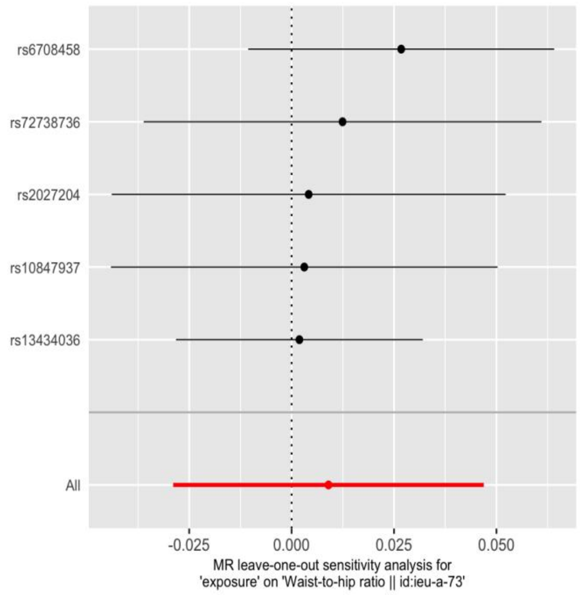

D. Leave-one-out analysis of PTB on WHR
